# Pharmacological Significance, Medicinal Use, and Toxicity of Extracted and Isolated Compounds from *Euphorbia* Species Found in Southern Africa: A Review

**DOI:** 10.3390/plants14030469

**Published:** 2025-02-05

**Authors:** Ipeleng Kopano Rosinah Kgosiemang, Relebohile Lefojane, Ayodeji Mathias Adegoke, Oludare Ogunyemi, Samson Sitheni Mashele, Mamello Patience Sekhoacha

**Affiliations:** 1 Unit for Drug Discovery Research, Department of Health Sciences, Faculty of Health and Environmental Sciences, Central University of Technology, Bloemfontein 9301, South Africa; kopanokgosiemang@gmail.com (I.K.R.K.); lefojanerelebohile@outlook.com (R.L.); smashele@cut.ac.za (S.S.M.); 2 Department of Pharmacology, Faculty of Health Sciences, University of the Free State, Bloemfontein 9301, South Africa; adegoke.am@ufs.ac.za; 3 Cancer Research and Molecular Biology Laboratories, College of Medicine, University of Ibadan, Ibadan 200005, Nigeria; 4 Nutritional and Industrial Biochemistry Research Unit, Department of Biochemistry, College of Medicine, University of Ibadan, Ibadan 200005, Nigeria; om.ogunyemi@mail1.ui.edu.ng

**Keywords:** *Euphorbia* species, toxicity, secondary metabolites, pharmacological properties, medicinal plants, cancer

## Abstract

This study documents the Euphorbiaceae family of plants in Southern Africa, with a focus on their traditional medicinal applications, pharmacological properties, toxicity, and active secondary metabolites. A review of the literature from scientific journals, books, dissertations, and conference papers spanning from 1962 to 2023 was conducted for 15 *Euphorbia* species. Recent findings indicate that specific compounds found in *Euphorbia* plants exhibit significant biological and pharmacological properties. However, the white sticky latex sap they contain is highly toxic, although it may also have medicinal applications. Phytochemical analyses have demonstrated that these plants exhibit beneficial effects, including antibacterial, antioxidant, antiproliferative, anticancer, anti-inflammatory, antiviral, antifungal, and anti-HIV activities. Key phytochemicals such as euphol, cycloartenol, tirucallol, and triterpenoids contribute to their therapeutic efficacy, along with various proteins like lectin and lysozyme. Despite some Euphorbiaceae species undergoing screening for medicinal compounds, many remain insufficiently examined, highlighting a critical gap in the research literature. Given their historical usage, further investigations are essential to evaluate the medicinal significance of *Euphorbia* species through detailed studies of isolated compounds and their pharmacokinetics and pharmacodynamics. This research will serve as a valuable resource for future inquiries into the benefits of lesser-studied *Euphorbia* species.

## 1. Introduction

The Euphorbiaceae is a highly varied group of flowering plants, having over 300 genera and 8000 species [1]. Within this family, the *Euphorbia* genus stands out as one of the largest, with more than 2000 species [2]. These species can take the form of herbs, shrubs, and trees, sometimes resembling succulents or cacti. They are mainly found in tropical and subtropical regions of Africa and America, as noted by Adedapo et al. [3]. Many species in this family produce a toxic white sap, but this sap can also have medicinal properties [4]. The genus contains economically significant species, making it a vital genus with high research potential.

South Africa has a rich collection of *Euphorbia* species, with at least 188 indigenous to the country (SANBI). The *Euphorbia* genus has attracted the attention of many researchers due to its diverse chemical compositions, which include euphol, triterpenoids, diterpene ester, and tirucallol. These compositions have been found to have remarkable therapeutic properties such as antimicrobial, antidiabetic, antiviral, and anticancer properties, as noted by Betancur-Galvis et al. [5] and also Mwine and Damme [6]. However, there is a lack of scientific documentation on the anticancer properties of many *Euphorbia* species in Southern Africa. This is due to a lack of research on isolated compounds, as well as a lack of testing and discovery of new compounds. Furthermore, there is limited research on unexplored *Euphorbia* species that may have potential anticancer properties. Additionally, the majority of published articles on *Euphorbia* focus on already explored species, with little to no information on the unexplored ones [7,8]. It is worth noting that there is currently only one comprehensive review available on the *Euphorbia* species found in Southern Africa. This review highlights the limited scientific documentation available on these plants and emphasizes the need for further research to unlock their full potential. This review, however, does not focus on the compounds found in the selected plants or their anticancer properties [9]. Therefore, the current study focuses on gathering more data on the use of isolated compounds as anticancer lead agents in traditional medicine to advance the research in the field of discovery and development of anticancer agents.

For this reason, the purpose of this review is to provide a comprehensive overview of the pharmacological activities of the isolated compounds from various *Euphorbia* species found in Southern Africa. This review also aims to highlight the diversity of the Euphorbiaceae family and its potential for further research. This study focuses on several plants, including *Euphorbia trigona* (*E. trigona*)*, Euphorbia tirucalli* (*E. tirucalli*), *Euphorbia ammak* (*E. ammak*), *Euphorbia bupleurifolia* (*E. bupleurifolia*), *Euphorbia enopla* (*E. enopla*), *Euphorbia polygona* (*E. polygona*), *Euphorbia cooperi* (*E. cooperi*), *Euphorbia stellata* (*E. stellata*), *Euphorbia ferox* (*E. ferox*), *Euphorbia clavariodes* (*E. clavarioides*), *Euphorbia gorgonis* (*E. gorgonis*), *Euphorbia coerulescens* (*E. coerulescens*), *Euphorbia horrida* (*E. horrida*), *Euphorbia arabica* (*E. arabica*), and *Euphorbia ledienii* (*E. ledienii*). These plants were selected for their pharmacological activities and toxicological evaluation on breast cancer cells as part of the authors’ recent research project. This review serves as a valuable resource for other researchers to explore the remaining *Euphorbia* species for their potentially active compounds and anticancer therapeutic properties.

## 2. Data Collection

To conduct this review, original articles from journals indexed in PubMed Central, Springer Link, Scopus, Science Direct, Hindawi, and Google scholars on *Euphorbia* species, their medicinal uses, toxicities, and pharmacological properties, were analyzed. For this review, only texts published in English between 1962 and 2023 were considered. The years of publication were selected due to the limited data available on *Euphorbia* plants. Throughout the search process, 881 reports were found across various databases. After reviewing the titles and abstracts, the search was refined and narrowed down to 323 articles, which were fully examined and discussed in this study.

The research criteria for inclusion were based on the guidelines set by Lu et al. [10] with a few modifications. The criteria included (1) studies on the isolated compounds of *Euphorbia* species conducted in vitro and in vivo; (2) evaluation of their biological activities; (3) studies published in peer-reviewed journals written in English; and (4) studies that provided full-text papers. There were no limitations on the study location. This study excluded (1) human studies, (2) a combination of isolated compounds, and (3) non-original articles.

The retrieved data were carefully examined to identify possible medical applications, biological activities, isolated compounds, toxicities, and pharmacological properties. Additional confirmation of the correct plant names was verified from the plantlist.org website. After analyzing all the data collected, conclusive results were obtained.

The cytotoxic tendency of selected compounds on various cancer cell lines and human tumor types was computed using the Cell Line Cytotoxicity Predictor (CLCPred) webserver (https://www.way2drug.com/Cell-line/) (accessed on 15 November 2024), a free online service for in silico evaluation of human cell line cytotoxicity tendency of bioactive compounds [11]. This prediction was based on Prediction of Activity Spectra for Substances (PASS) technology (https://www.way2drug.com/PASSonline) (accessed on 15 November 2024), where the training set was generated based on cytotoxicity data derived from ChEMBLdb (version 23) (https://www.ebi.ac.uk/chembldb/) (accessed on 15 November 2024). Compounds with robust anticancer potential were selected and subjected to further analyses to extract physicochemical and pharmacokinetics parameters using the SwissADME (http://www.swissadme.ch/) (accessed on 18 January 2025) [12] and pKCSM (http://biosig.unimelb.edu.au/pkcsm/) (accessed on 15 November 2024) [13] webservers, as demonstrated earlier [14,15].

## 3. Results

The current study reports on 15 *Euphorbia* species, their traditional and pharmacological properties, and also isolated compounds. *Euphorbia* species have been used in folk medicine for various ailments, which is why scientists have taken an interest in investigating this genus and fully documenting the secondary metabolites responsible for these properties; see Table 1.

Out of the 15 plant species examined, 8 have been traditionally used as medicine for treating seven distinct diseases. The most commonly used plants are for treating cancer (seven), followed by warts and wounds (five) and other ailments; see Table 1.

An investigation into the chemical composition of various *Euphorbia* species unveiled distinctive profiles of isolated compounds. *E. tirucalli* exhibited the highest diversity with a total of 30 isolated compounds, spanning triterpenoids (Lupane, Oleanane, Tirucallane, Phorbol-type), phenolic compounds (Gallic Acid Derivatives, Ellagic Acid Derivatives, flavonoids), phytosterols (sterols), glycosides (triterpene glycosides, Other Glycosides), and diterpene esters [9,37]. Following closely, *E. cooperi* presented 18 compounds, including triterpenoids (Lupane, Oleanane, Phorbol-type), phytosterols (sterols), and glycosides (triterpene glycosides) [24]. *E. trigona* showcased 16 compounds, including triterpenoids (Lupane, Oleanane, Phorbol-type), phenolic compounds (Gallic Acid Derivatives, Ellagic Acid Derivatives, flavonoids), phytosterols (sterols), and glycosides (Other Glycosides) [17,70]. *E. coerulescens* and *E. ledienii* exhibited nine and eight compounds, respectively, encompassing triterpenoids, phenolic compounds, and phytosterols [66,71], see Table 2 and Table 3. Notably, certain plants demonstrated fewer or no isolated compounds.

The breakdown of these compounds into subclasses revealed diverse chemical categories, including Euphane-type Triterpenoids (euphol, euphorbol), Cycloartane-type Triterpenoids (cycloartenol, cycloartanol, 24-ethylene cycloartanol), Lupane-type Triterpenoids (lupeol), Oleanane-type Triterpenoids (α-amyrin, β-amyrin), Pentacyclic Triterpenes (betulinic acid, Glut-5-en-3-β-ol), Taraxarane-type Triterpenoids (taraxerol, taraxerol acetate), Phorbol-type Diterpenoids (12-Deoxyphorbol-13-isobutyrate-20-acetate, Phorbol, 12-Deoxy phorbol, 12-Deoxy-16-hydroxy phorbol, 12-Deoxyphorbol esters, 12, 20-Dideoxyphorbol-13-isobutyrate), Tirucallane-type Triterpenoids (tirucalicine, tirucallol, tirucallin A, tirucallin B), Steroidal Triterpenoids (terpenic alcohol), Ingenane-type Triterpenoids (Ingol-7,8,12-acetate, ditiglate, ingenol, ingenol triacetate, Angelate acetate isobutyrate), flavonoids (tri-methyl ellagic acid, 17-Hydroxyingenol-17-benzoate-20-angelate, Kampferol-3-O-ß-D-rutinoside), sterols (β-sitosterol, stigmasterol, taraxasterol), Ellagic Acid Derivatives (ellagic acid, 3,3′-Di-O-methylellagic acid), Medium-chain Fatty Acids (laurate), and no specific subclass records (rhoiptlenone, 2-Methylbutyric acid, euphorbin A, euphorbin B, euphorbol hexacosonate, 12-Deoxy-4β-hydroxyphorbol-13-phenyl acetate-20-acetate, 20-Acetoxy-16-angeloyloxy13α-isobutanoyloxy-4β,9α,20-tetrahydroxytiglia-1,5-diene-3-one, resin, arachiside A, 3,4,4′ Trimethoxyellagic acid, 12-Deoxyphorbol-13-(2-methylbutyrate)-20-acetate, 2-Methylbutyric acid) (see Table 4).

*E. tirucalli* as well as *E. ammak* are among the *Euphorbia* species that received substantial research attention. These findings are in agreement with studies conducted by Mavundza et al. [9], which stated that *E. tirucalli* was among the most studied species.

The investigation into the chemical composition of various *Euphorbia* species is presented in Table 4, which revealed a diverse array of isolated compounds, each showcasing unique pharmacological and biological activities. Euphol, classified within the Euphane subclass, demonstrates a diverse profile, including anticancer, cytotoxicity, anti-nociceptive, anti-inflammatory, and HIV-1 reverse transcriptase inhibitor activities [76,102,103]. The cycloartenol and cycloartanol compounds, falling under the Cycloartane subclass, exhibit a wide range of effects such as anti-inflammatory, antitumor, antioxidant, antibiosis, anti-Alzheimer’s disease, apoptotic, analgesic, and antifungal activities [104,105,106,107,108,109,110]. Lupeol, belonging to the Lupane subclass, displays diverse properties like anticancer, anti-inflammatory, antimicrobial, antiprotozoal, antiproliferative, antiangiogenic, and cholesterol-lowering effects [40,111,112,113,114,115,116,117]. Oleanane-type triterpenoids, represented by α-amyrin and β-amyrin, demonstrate activities such as cytotoxicity, antifungal, anti-inflammatory, nitric oxide inhibition, reactive oxygen species activation, and anticancer effects [118,119,120,121,122,123,124,125]. Pentacyclic triterpenes, including betulinic acid, exhibit antitumor, antidiabetic, anti-inflammatory, HIV-1 reverse transcriptase inhibition, antiviral, and hepatoprotective activities [126,127,128,129,130,131,132,133,134,135]. The Taraxarane subclass, represented by compounds like taraxerol and taraxerol acetate, showcases properties like anticancer, anti-inflammatory, apoptotic, antioxidative, antimicrobial, antifungal, and antidiabetic effects [136,137,138,139,140,141]. Sterols, represented by β-sitosterol, display a spectrum of activities, including anti-inflammatory, anticancer, antiproliferative, analgesic, and antimicrobial effects [125,142,143,144,145]. Tirucallane-type triterpenoids, such as tirucalicine and tirucallol, currently have no specific records of biological activity [146,147]. Terpenic alcohol, a steroidal triterpenoid, demonstrates antibacterial and irritant effects [148,149]. Ellagic Acid Derivatives, including tri-methyl ellagic acid, exhibit anticancer properties [148,150,151]. Ingenane-type triterpenoids, represented by ingenol and its derivatives, showcase cytotoxicity and HIV-1 reverse transcriptase inhibition [102,152]. Various subclasses such as Taraxarane, Euphane-type, sterol, Tirucallane, and Phorbol-type exhibit diverse pharmacological activities, including anti-inflammatory, antifungal, antibacterial, hepatoprotective, antioxidant, antiproliferative, and anti-HIV effects; see Table 5.

*E. trigona* has the most isolated anticancer compounds (14), followed by *E. tirucalli* (13) and *E. cooperi* (8), and the others have fewer or none. The *Euphorbia* species found in Southern Africa are rich sources of various types of bioactive compounds, including triterpenoids, phorbol esters, alkaloids, flavonoids, phytosterols, glycosides, and saponins [9,60,65]. Furthermore, minor classes that were isolated from *Euphorbia* species from Southern Africa include anthraquinone, polyphenols, and tannins [9,60,65].

It has further been reported that most spurges contain an acidic and burning vesicant juice, as well as cyanoglycosides, which can be toxic [203]. Reports have shown that the ingestion of a large quantity of the latex may cause gastro-intestinal hemorrhage and even result in death [41]. Although incidents of poisoning in children and animals are rare, it is important to handle these plants with great caution [69]. The latex from these plants can also cause blisters on the skin and temporary blindness [34]. Furthermore, they have been used as fish poison and bird-lime; see Table 6.

**Table 6:** Type of toxicity caused by some *Euphorbia* species.

Type of Toxicity	*Euphorbia* Species	References
Fish poisoning	*Euphorbia scheffleri* Pax, *Euphorbia tirucalli* L., and *Euphorbia inaequilatera* Sond	[69]
Human poisoning	*Euphorbia ledienii* A. Berger, *Euphorbia heterophylla* L., *Euphorbia cooperi* N.E.Br. ex A. Berger, *Euphorbia candelabrum* Kotschy, *Euphorbia virosa* Willd., *Euphorbia poissonii* Pax, *Euphorbia unispina* N.E.Br., and *Euphorbia venenifica* Tremaux ex Kotschy	[69]
Domestic animals poisoning	*Euphorbia caput-medusae* L., *Euphorbia silenifolia* (Haworth) Sweet, *Euphorbia ingens* E. Mey. Ex Boiss; as well as irritating ones: *E. tirucalli*, *Euphorbia poissonii*, *Euphorbia unispina*, and *E. venenifica*. I	[69]
Carcinogen/promotor of cell division	*E. tirucalli*, *Euphorbia leuconeura*, and *J. Curcas*	[167,204,205]
Conjunctivitis	*E. tirucalli* and *Euphorbia royleana*	[39,206]

The following taxonomic classification of all *Euphorbia* plants is discussed in this review:

Domain: Eukaryote, Kingdom: Plantae, Order: Malpighiales, family: Euphorbiaceae, and genus: *Euphorbia* (https://www.mindat.org/taxon-4691.html) (accessed on 3 March 2024).

## 4. Discussion

### 4.1. Ethnopharmacological Use, Phytochemistry, and Toxicity

#### 4.1.1. *Euphorbia trigona*

*E. trigona*, a plant native to Central Africa, tropical Africa, and India, has been traditionally utilized in Ayurvedic medicine to treat respiratory and urinary tract infections, as well as gonorrhea (see Figure S1A) [18]. Research conducted by Nashikkar et al. [17] demonstrated that *E. trigona* is effective in addressing various ailments, including tumors, warts, intestinal parasites, rheumatoid arthritis, hepatitis, and inflammation. A combination of its roots and ginger is recommended for consumption in the morning to alleviate piles [19]. Additionally, some individuals utilize latex drops in palm wine for the relief of severe constipation or during epileptic seizures [20].

Phytochemistry

The plant *E. trigona* was analyzed for its phytochemical composition and was found to contain saponins, alkaloids, flavonoids, glycosides, sterols, and triterpenoids [99]. Additional studies conducted by Nashikkar et al. [17] also identified the presence of sterols, alkaloids, flavonoids, and saponins, as well as tannins, which were not detected by [99]. The absence of tannins in the earlier study may be attributed to environmental factors. Nielsen et al. [71] reported that the latex of *E. trigona* contains a high level of sterols, with the primary components being euphol and cycloartenol. Anjaneyulu and Rao [70] isolated several triterpenoids from the latex, including euphol, cycloartanol, cycloartenol, lupeol, α-amyrin, and β-amyrin. They also identified five diterpene esters known to be skin irritants. Furthermore, *E. trigona* is recognized as a good source of lectin, which has demonstrated potency in human erythrocyte agglutination [21].

Toxicity

A study conducted by EL-Hawary et al. [98] evaluated the cytotoxic effects of a methanolic extract of *E. trigona* against the HEPG2, MCF-7, and CACO2 cell lines. The findings revealed a pronounced cytotoxic effect on MCF-7 and CACO2 cell lines, with IC50 values of 16.1 and 15.6 µg/mL, respectively. In 2022, Anju and Rameshkumar assessed the cytotoxic effects of a methanol extract on the HeLa and H9C2 cell lines but found no significant cytotoxic effect on either cell line [156]. Another study examined the cytotoxicity of Hex, DCM, MeOH, and EtoAc extracts against Vero cell lines [65]. The results indicated that none of the four extracts from *E. trigona* exhibited cytotoxicity, as they did not inhibit 50% of the cell growth at concentrations of 10 µg/mL and below.

#### 4.1.2. *Euphorbia ledienii*

*E. ledienii* is a plant species indigenous to the Western Cape of South Africa (see Figure S1B). Currently, there is no available traditional or pharmacological data regarding this plant.

Phytochemistry

Despite the absence of traditional or pharmacological studies, several compounds have been identified in *E. ledienii*. These include 12-Deoxyphorbol-13-isobutyrate-20-acetate and 12-Deoxyphorbol-13-(2-methylbutyrate)-20-acetate [72]. Evans and Kinghorn [74] reported three variants: phorbol, 12-Deoxyphorbol, and 12-Deoxy-16-hydroxy phorbol. Additionally, *E. ledienii* has been found to contain Ingol-7,8,12-acetate and ditiglate [160]. Redei et al. [73] identified isobutyric and 2-methylbutyric acids in the plant. Other studies have revealed several hydrolytic proteins in *E. ledienii*, including N-acetyl-β-glucosamidase, chitobiosidase, endochitinase, and lysozyme activity [207]. According to Domsalla et al. [208], *E. ledienii* exhibits high proteolytic activity.

Toxicity

It is essential to note that *E. ledienii* is highly toxic to humans, with the potential to cause skin irritation [69]. Moreover, none of the isolated compounds have undergone toxicity assessments to date.

#### 4.1.3. *Euphorbia horrida*

*E. horrida* is a plant species native to Wittepoort/Karoo, South Africa (see Figure S1C). Currently, there is no known traditional or pharmacological information available regarding this plant.

Phytochemistry

In a study by El-Hawary et al. [98], 17-Hydroxyingenol-17-benzoate-20-angelate was isolated from the plant. *E. horrida* also contains diterpene esters, which are known to cause skin irritation. Additionally, Mampa et al. [65] identified various other classes of compounds in the plant, including phytosterols, pentoses, tannins, glycosides, triterpenoids, anthraquinones, saponins, flavonoids, and alkaloids.

Toxicity

The cytotoxic effects of a dichloromethane (DCM) extract derived from *E. horrida* var. were evaluated using a Vero cell line. The results demonstrated significant cytotoxicity, with an IC50 value of 10 µg/mL, indicating its potential cytotoxic effects [65]. Conversely, a study conducted by El-Hawary et al. [98] assessed the cytotoxicity of a methanol extract of *E. horrida* against the HeLa and H9C2 cell lines, revealing no significant effects.

#### 4.1.4. *Euphorbia enopla*

*E. enopla,* a plant native to the Eastern Cape and the semiarid Karoo regions of South Africa, has no documented traditional or pharmacological data available (see Figure S1D).

Phytochemistry

The plant contains euphol and tirucallol, which were isolated by Ponsinet and Ourisson [93]. Moreover, Mampa et al. [65] extracted phytosterols, glycosides, triterpenoids, flavonoids, alkaloids, tannins, and anthraquinones. Sytwala et al. [207] isolated hydrolytic proteins, including N-acetyl-β-glucosamidase, chitobiosidase, endochitinase, and lysozyme.

Toxicity

A study conducted by Mampa et al. [65] assessed the toxicity of the hexane extract of *E. enopla* using the Vero cell line. The results indicated that the extract had a significant inhibitory effect on cell growth, particularly at a concentration of 10 µg/mL. Notably, the highly non-polar hexane fraction exhibited the most potent effects [65].

#### 4.1.5. *Euphorbia coerulescens*

*E. coerulescens*, native to the Cape Province of South Africa, lacks documented traditional or pharmacological data (see Figure S1E). However, several compounds have been isolated from this species.

Phytochemistry

Studies conducted by Evans [66] led to the isolation of various compounds, including angelate acetate isobutyrate, acetate α-methyl butyrate, acetate laurate, α-methyl butyrate, heptanoate, and laurate. Additional research has resulted in the isolation of euphol, tirucallol, and euphorbol [89]. Furthermore, Sytwala et al. [207] isolated several hydrolytic proteins, such as N-acetyl-β-glucosamidase, chitobiosidase, endochitinase, and proteins exhibiting lysozyme activity. Lynn and Clevette-Radford [209] also identified homogeneous lectins.

Toxicity

In an irritancy test performed by Evans [66], it was observed that the latex of *E. coerulescens* induced ear inflammation in mice. Moreover, this latex may cause skin irritation, and ingestion can lead to a burning sensation in the throat. Direct contact with the eyes poses severe risks, including the potential for blindness. It is important to note that none of the isolated compounds have undergone toxicity evaluations to date.

#### 4.1.6. *Euphorbia cooperi*

*E. cooperi* is a plant species native to KwaZulu-Natal and Limpopo Province, South Africa (see Figure S1F). Research indicates its potential for treating various health conditions. The liquid extracted from the soaked roots and stems has been utilized as an enema for alleviating stomach pain and bloating [25]. In South Africa, the Venda tribe employs this plant for the treatment of paralysis and for application to infected wounds [25]. Historically, farmers have utilized *E. cooperi* to treat various bacterial infections in livestock [22,210]. Moreover, the latex of this plant is used for poisoning fish in Limpopo Province, South Africa [34,38].

Phytochemistry

Phytochemical analysis of *E. cooperi* has revealed that its latex contains numerous diesters and triesters [97]. The chloroform fraction of the plant’s latex was found to contain three previously unisolated compounds, i.e., euphol, obtusifoliol, and 12-deoxyphorbol-13-isobutyrate-16-angelate-20-acetate, which belong to the triterpene, steroid, and diterpenoid families, respectively [24]. Additionally, a study conducted by El-Toumy et al. [96] isolated 7-galloyl catechin, kaempferol 3-O-β-(6″-O-galloyl)-glucopyranoside, and triester 16-hydroxy-12-desoxy-phorbol from the flower of *E. cooperi*.

Examination of the aerial parts of *E. cooperi* has yielded interesting findings. Hlengwa [95] identified a unique norsesquiterpenoid called euphorbilactone, along with its glycoside, arachiside A. The researcher also identified a triterpenoid, glutinol; a known phorbol ester, 16-angeloyloxy-13α-isobutanoyloxy-4β,9α,20-trihydroxytiglia-1,5-diene-3,7-dione; and a new phorbol ester, 20-acetoxy-16-angeloyloxy-13α-isobutanoyloxy-4β,9α,20-tetrahydroxytiglia-1,5-diene-3-one. A comprehensive review of the existing literature has unveiled that several compounds such as euphol, obtusifoliol, 12-deoxyphorbol-13-isobutyrate-16-angelate-20-acetate, euphorbilactone, norsesquiterpenoid, arachiside A, glutinol, 16-angeloyloxy-13α-isobutanoyloxy-4β,9α,20-trihydroxytiglia-1,5-diene-3,7-dione, 20-acetoxy-16-angeloyloxy-13α-isobutanoyloxy-4β,9α,20-tetrahydroxytiglia-1,5-diene-3-one, bervifolin, carboxylic acid, kaempferol-3-O-β-D-rutinoside, 1-O-galloyl-3,6-hexahydroxydiphenyl-β-D-glucopyranoside, 3,3′-dimethoxy ellagic acid, and 3,4,4′-trimethoxyellagic acid have not been previously discussed.

Toxicity

A study conducted by El-Sherei et al. [24] evaluated the cytotoxic effects of the chloroform extract of *E. cooperi*. Their findings revealed that the chloroform extract exhibited significant cytotoxic effects against the MCF-7, HepG2, and HeLa cell lines. The IC50 values for these cell lines were 4.23, 10.80, and 26.6, respectively. These results align with those of Mavundza et al. [9], as no further research has been conducted to date.

#### 4.1.7. *Euphorbia tirucalli*

*E. tirucalli*, commonly referred to as milkbush, is a plant native to Eastern tropical Africa, South Africa, and the Indian Ocean Islands (see Figure S1G). It has demonstrated efficacy in treating a variety of medical conditions. Hargreaves [38] noted that *E. tirucalli* is employed to induce emesis in the context of snakebite treatment. Additional studies indicate that its latex has therapeutic applications for conditions such as sexual impotence, skin disorders, swollen glands, edema, hemorrhoids, rheumatoid arthritis, epilepsy, and both dental and otic pain, as well as for tumor management [39,40,211]. Moreover, the latex exhibits significant pharmacological properties, including antibacterial, molluscicidal, antiherpetic, and antimutagenic effects [5,40,44,45,46,47,48,49,177]. Research has demonstrated that extracts from *E. tirucalli* possess myelomodulatory activity and inhibit colony formation [50]. Its latex is also known to contain compounds that exhibit antitumor effects across various cell lines [51,52]. Notably, *E. tirucalli* has been patented as a potential therapeutic agent for prostate cancer [6].

Phytochemistry

Extensive studies have analyzed the chemical compounds of *E. tirucalli*, and diterpenes have been identified as the primary isolated compound in all parts of the plant [9]. The latex of *E. tirucalli* contains several phytoconstituents, including triterpenes euphol, diterpene esters of phorbol, 12-Deoxyphorbol esters and ingenol, β-sitosterol, euphorbol hexacosonate, 12-Deoxy-4β-hydroxyphorbol-13-phenylacetate-20-acetate, 12, 20-Dideoxyphorbol-13-isobutyrate, glut-5-en-3-β- and euphol, 12-O-2Z-4E-octadienoy1-4-deoxyphorbo1-13-acetate, cycloart-23-ene-3-β-, 25-diol, Euphorcinol, 4-Deoxyphorbol di-ester, cycloeuphordenol, cyclotirucanenol, diterpene ester, serine proteases, euphol, steroids, tirucalicine, tri-methyl ellagic acid, terpenic alcohol, isoeuphorol, taraxasterol, tirucallol (fresh latex), ketone euphorone, and resin [8,21,75,76,78,80,81,82,83,84,86,87,88,89,90,91]. Researchers have identified several compounds in the stem of *E. tirucalli*, including ellagic acid, taraxerol, 3,3′-Di-O-methylellagic acid, β-sitosterol, euphorbin A (a type of polyphenol), euphorbin F (dimers), tirucallin A (a type of tannin), and tirucallin B. [79,80,92]. Euphorbiane which is a triterpenoid was isolated from the stem [77]. Rasool et al. [85] isolated euphorginol from the stem bark of *E. tirucalli*. The bark, on the other hand, was found to contain phorbol, β-sitosterol, cycloartenol, 24-Methylene cycloartenol, and ingenol triacetate [35]. Other studies have revealed that β-amyrin is present in the leaves of this plant, as reported by Kajikawa et al. [36]. In addition, Shivkumar [37] found various compounds such as phenols, flavonoids, tannins, alkaloids, saponins, glycosides, triterpenes, and steroids. However, Kgosiemang et al. [100] isolated only tannins, glycosides, triterpenoids, and saponins from the same plant. Upon review, it was noted that several compounds, such as diterpene esters, 12-Deoxyphorbol esters, ingenol, hexacosonate, 12-Deoxy-4β hydroxyphorbol-13- phenyl acetate -20- acetate, 12, 20 Dideoxyphorbol-13 isobutyrate, tirucalicine, tri-methyl ellagic acid, terpenic alcohol, isoeuphorol, taraxasterol, tirucallol, ketone euphorone, resin, ellagic acid, 3,3′-Di-O-methylellagic acid, euphorbin A, euphorbin B, tirucallin A, tirucallin B, cycloartenol, 24-Methylenecycloartenol, ingenol triacetate, rhoiptlenone, 3β-friedelinol, epi-friedelinyl acetate, 24-ethylene cycloartanol, friedelan 3 α- and 3 βα-ols, taraxerol acetate, betulinic acid, α-amyrin, lupeol, cycloartanol, and β-amyrone were not discussed in other reviews compared to the current review [9].

Toxicity

Silva et al. [212] assessed the antitumor effects of euphol derived from *E. tirucalli* against a diverse range of human cancer cell lines. The study demonstrated that euphol exhibits cytotoxic properties, with IC50 values ranging from 1.41 to 38.89 µM. The highest efficacy was observed in esophageal squamous cell lines (11.08 µM) and pancreatic carcinoma cells (6.84 µM), with notable effects also recorded in prostate, melanoma, and colon cancer cells. Letícia et al. [213] evaluated the antiproliferative efficacy of *E. tirucalli* extracts against leukemia (HL-60), lymphoma (Daudi), and melanoma (B16F10) cell lines using the MTT assay at concentrations of 62, 125, 250, and 500 μg/mL. The results indicated a significant regional variation in extract cytotoxicity, demonstrating a dose-dependent pattern. The extracts exhibited comparable effectiveness against the leukemia cell line HL-60, reducing cell viability to approximately 60–70%. In a separate study, researchers investigated the antiproliferative effects of highly diluted latex and *E. tirucalli* homeopathic remedies on melanoma cells in vitro. Solutions of 0.5% and 5% concentrations in 70°GL ethanol were prepared for use. The findings revealed that the 0.5% latex solution at 30cH reduced melanoma cell growth by 19.7%, while the 0.5% *E. tirucalli* solution at 30cH exhibited a 32.1% reduction [214]. Additionally, Waczuk et al. [211] evaluated the cytotoxic effects of an aqueous extract derived from *E. tirucalli* on human leukocytes, with results indicating that exposure to high concentrations of the extract significantly reduced cell viability. A study by Abdel-Aty et al. [215] assessed the cytotoxicity of phenolic compounds from *E. tirucalli* against various cancer cell lines (HepG2, MCF-7, A549, HL-60, HCT116) and the normal human melanocyte HFB4. The results indicated that low concentrations of the phenolic content exhibited significant cytotoxicity against HL-60, with an IC50 value of 22.76 ± 2.85 μg/mL. Furthermore, the extract showed moderate cytotoxicity against MCF-7 and A549 cells, with IC50 values of 31.65 ± 3.67 and 35.36 ± 3.82 μg/mL, respectively. A thorough evaluation of the antiproliferative potential of the methanolic extract on the MiaPaCa-2 cancer cell line demonstrated a remarkable ability to significantly inhibit the growth of MiaPaCa-2 cancer cells [216]. The presented toxicity findings align with those reported by Mavundza et al. [9].

#### 4.1.8. *Euphorbia ammak*

*E. ammak* is a plant native to Saudi Arabia and the Yemen Peninsula (see Figure S1H) [29].

Phytochemistry

Abdel-Sattar et al. [94] conducted a screening of the leaves of *E. ammak* and identified three primary compounds: euphol, α-glutinous, and stigmasterol. Additionally, Ponsinet and Ourisson [93] isolated euphol and euphorbol from the plant. Al-Hajj et al. [31] detected the presence of alkaloids, saponins, and glycosides. These studies suggest that *E. ammak* possesses significant antileishmanial activity against cutaneous leishmaniasis.

Toxicity

Research has demonstrated that the methanolic extract of *E. ammak* may inhibit the H1N1 influenza virus and exhibit considerable cytotoxic activity against MDCK cells [32]. Abdel-Sattar et al. [33] reported that the methanolic extract displays antiparasitic properties, with the euphol compound showing substantial cytotoxic effects against various human cancer cell lines in vitro. Mampa et al. [65] assessed the effects of four distinct extracts of *E. ammak* on cell proliferation. These extracts were obtained using several solvents, including hexane, dichloromethane (DCM), methanol (MeOH), and ethyl acetate (EtOAc). The findings indicated that the DCM extract exhibited the most significant inhibition of cell growth at concentrations as low as 1 µg/mL. Almehdar et al. [30] assessed the cytotoxic effects of *E. ammak* latex on MCF-7 breast cancer cells and found that the latex exhibited significant toxicity, with an IC50 value of 14.3.

#### 4.1.9. *Euphorbia clavarioides*

*E. clavarioides* is a plant species native to South Africa and Lesotho (see Figure S1I). Traditionally, it has been employed to treat various dermatological conditions, including rashes in children, acne, sores, bruises, burns, eczema, ulcers, cracked heels, and wounds [53,54,217]. A study conducted by Mbhele [218] validated its efficacy in wound healing, thereby supporting its traditional applications. In Lesotho, *E. clavarioides* is also used for bathing swollen feet and, when combined with Berkheya onopordifolia, it serves as a treatment for leprosy [218]. Additional studies suggest potential applications in managing herpes, HIV-related infections, hypertension, and diabetes [55,56,57].

Phytochemistry

Moteetee et al. (2019) performed a phytochemical analysis of *E. clavarioides* and identified the presence of alkaloids, flavonoids, saponins, terpenoids, and tannins [53]. In a subsequent study conducted in 2020, Mampa et al. isolated phytosterols, glycosides, triterpenoids, anthraquinones, flavonoids, and alkaloids from the same species. Despite these findings, there remains a notable gap in comprehensive research on this species [65].

Toxicity

In the study conducted by Mampa et al. [65], the effects of hexane (Hex), dichloromethane (DCM), methanol (MeOH), and ethyl acetate (EtOAc) extracts of *E. clavarioides* on cell proliferation were evaluated using the Vero cell line. The Hex and DCM extracts demonstrated the highest inhibition of cell growth in Vero cells.

#### 4.1.10. *Euphorbia gorgonis*

*E. gorgonis* is a plant species that is native to the Eastern Cape of South Africa (see Figure S1J). Studies have shown that it has medicinal properties and is effective in treating cancer, wounds, swelling, and various skin conditions [58,59]. Furthermore, it has been reported to exhibit significant antibacterial and antimicrobial activity [60].

Phytochemistry

Studies identified its phytochemical constituents to include phytosterols, glycosides, triterpenoids, flavonoids, and alkaloids [65]. In addition, Tiwani [60] highlighted the presence of tannins, saponins, alkaloids, and flavonoids.

Toxicity

An evaluation of *E. gorgonis* extracts on human intestinal cancer cells indicated that aqueous extracts caused a reduction in cell numbers, whereas acetone and ethanol extracts showed cytotoxic effects and led to a decrease in cell viability. In another study, the acetone extract was shown to lower cell viability in rat hepatoma cells, while the aqueous extract maintained a high percentage of cell viability [60]. Mampa et al. [65] assessed the cytotoxic effects of hexane, DCM, MeOH, and EtOAc extracts on a Vero cell line and reported that none of the extracts were toxic to the cell line.

#### 4.1.11. *Euphorbia bupleurifolia*

*E. bupleurifolia* is a plant indigenous to South Africa, primarily located in the Eastern Cape Province and Natal (see Figure S1K). The milky latex produced by this plant has been historically utilized in the treatment of various ailments, including cancerous sores, painful cracked feet, eczema, pimples, rashes, and wounds [61]. Moreover, the twigs of *E. bupleurifolia* have been employed as a teeth-cleansing agent [61]. Furthermore, the plant has been reported to alleviate swelling in the lower limbs and to serve as a treatment for cancer [62,63]. Some anecdotal evidence suggests its use in managing retained placenta [64].

Phytochemistry

Previous research has identified a range of secondary metabolites present in *E. bupleurifolia*, including phytosterols, tannins, glycosides, triterpenoids, saponins, flavonoids, and alkaloids [65]. Van Wyk et al. [62] specifically documented the presence of triterpenes within this species.

Toxicity

A study conducted by Mampa et al. [65] assessed the potential cytotoxic effects of *E. bupleurifolia* extracts on the Vero cell line. The results indicated that both hexane and dichloromethane extracts exhibited antiproliferative effects on the Vero cell line, showing significant efficacy at concentrations of 1 and 10 µg/mL, respectively.

#### 4.1.12. *Euphorbia polygona*

*E. polygona* is a plant species native to the Eastern Cape region of South Africa (see Figure S1L). Currently, there is a lack of documented information regarding its traditional uses and pharmacological properties.

Phytochemistry

A study conducted by Mampa et al. [65] identified several phytochemical constituents in the plant, including phytosterols, tannins, glycosides, triterpenoids, flavonoids, and alkaloids.

Toxicity

Mampa et al. [65] also investigated the antiproliferative effects of hexane (Hex), dichloromethane (DCM), methanol (MeOH), and ethyl acetate (EtOAc) extracts of *E. polygona* on the Vero cell line. The results demonstrated that the Hex and DCM extracts exhibited the most significant antiproliferative effects compared to the MeOH and EtOAc extracts.

#### 4.1.13. *Euphorbia arabica*

*E. arabica* is distributed across several regions, including Botswana, southern Mozambique, Zimbabwe, and South Africa (refer to Figure S1M). Historically, this plant has been employed as an antibacterial agent, as documented by El-Shanwani [68]. Moreover, it has been utilized in the treatment of various ailments, such as warts and stomachaches. Furthermore, the juice derived from *E. arabica* has been applied for the management of skin infections [67,68].

Phytochemistry

A recent study conducted by Mampa et al. [65] identified the presence of phytosterols, tannins, glycosides, triterpenoids, anthraquinones, and flavonoids within *E. arabica*.

Toxicity

The cytotoxic effect of Hex, DCM, MeOH, and EtOAc extracts of *E. arabica* were evaluated against a Vero cell line. The findings of the study indicated that the hexane extract of *E. arabica* inhibited cell growth of the Vero cell line, achieving IC50 at all concentrations tested. It is worth noting that the DCM extract showed IC50 at a concentration of 10 µg/mL.

#### 4.1.14. *Euphorbia ferox*

*E. ferox* is a plant species native to the Western Cape region of South Africa (see Figure S1N). At present, there is a lack of available information regarding its traditional uses, pharmacological activities, and the isolated compounds associated with *E. ferox*.

#### 4.1.15. *Euphorbia stellata*

*E. stellata* is a plant species found in the Eastern Cape region of South Africa (see Figure S1O). Currently, there is no documented information on the traditional uses, pharmacological applications, or isolated compounds derived from *E. stellata*.

### 4.2. Pharmacological Activities

The utilization of medicinal plants has garnered significant attention due to their efficacy in treating a variety of ailments, many of which are supported by scientific evidence. Natural products hold substantial importance owing to their diverse biological activities and drug-like properties, facilitating the development of new lead compounds, natural drugs, pharmacological tools, and herbal remedies [219]. The therapeutic attributes of these plants are largely ascribed to their unique bioactive compounds.

Numerous species of the genus *Euphorbia* are recognized for their medicinal properties, with over 5% employed in the treatment of ailments such as warts, wounds, skin disorders, tumors, respiratory issues, sexually transmitted infections, urinary tract infections, and intestinal parasites worldwide [17,24,25,28]. This prevalence may be attributed to the presence of distinctive secondary metabolites and isolated compounds [220]. However, it is critical to note that the latex produced by most *Euphorbia* species is toxic and can induce severe skin irritation and potentially lead to blindness. Despite its toxicity, this latex contains biologically active compounds, including terpenes, diterpenoids, and triterpenes [221].

Several *Euphorbia* species have demonstrated notable medicinal properties, with their extracts patented as prescription drugs. For instance, the extract of *E. lathyrism* (US 5707631) is utilized in the treatment of arthritis, hyperlipidemia, Alzheimer’s disease, and hypertension. Extracts from *E. peplus*, *E. hirta*, and *E. drummondii* (US 6844013) have exhibited selective cytotoxicity against various cancer cell lines, and their compounds are employed in the treatment of malignant melanomas and squamous cell carcinomas. Further, *E. aaron-rossii*, *E. tirucalli*, *E. tomentella*, and *E. tomentose* (US 2003/0171334 A1) are indicated in the management of prostate cancer. Additionally, the latex of *E. tirucalli* (US 2009/0142421 A1) has demonstrated potency in treating conditions related to cell proliferation or angiogenesis [222]. The extract from *E. obesa* (US 6923993) has been shown to stimulate apoptosis and inhibit cancer cell proliferation. *E. hirta* (US 2007/0248694 A1) is utilized for its anti-inflammatory properties, while *E. antiquorum* (US 2003/0165579 A1) has been identified to inhibit tumor growth.

This study analyzed 15 different species of *Euphorbia*, revealing a diverse array of secondary metabolites that hold significance in biomedical sciences. The analysis identified several phytochemicals, including diterpenoids, triterpenes, sesquiterpenoids, phloracetophenones, cerebrosides, glycerols, flavonoids, and steroids, alongside various isolated compounds (detailed in Table 5).

#### 4.2.1. Flavonoids

Flavonoids, a class of polyphenolic compounds, have been identified in nine species of Euphorbia, including *E. horrida*, *E. trigona*, *E. clavarioides*, *E. enopla*, *E. gorgonis*, *E. bupleurifolia*, *E. polygona*, *E. arabica*, and *E. tirucalli*. Moreover, flavonoids have also been observed in other members of the *Euphorbiaceae* family, such as *E. microsciadia*, *E. heterophylla*, *E. hirta*, *E. neriifolia*, *E. paralysis*, *E. lunulata*, and *E. larica*, indicating their widespread occurrence within this family. The extensive presence of flavonoids underscores their critical role in the therapeutic potential of Euphorbia species, significantly contributing to their pharmacological relevance.

Research has demonstrated that flavonoids possess medicinal properties beneficial for addressing various health conditions. These properties include anti-inflammatory effects, enzyme inhibition, antimicrobial activity, estrogenic effects, antiallergic responses, and antioxidant activity [223,224,225]. Moreover, *E. helioscopia* has been found to contain a substantial amount of flavonoids, exhibiting cytotoxic effects on triple-negative breast cancer cells [226]. Further studies indicate a reduction in the differentiation of 3T3-L1 preadipocytes, a decrease in triglyceride accumulation in mature adipocytes, and a reduction in nitric oxide production in RAW 264.7 cells [227]. It has been reported that flavonoids prevent the degradation of cAMP by phosphodiesterases, thereby prolonging cAMP signaling, which contributes to their anti-inflammatory properties [228]. Moreover, methanol extracts from *E. trigona* have been shown to induce cell death in MCF-7 and Caco-2 cells with IC50 values of 16.1 and 15.6 µg/mL, respectively [98]. Previous research has shown that the butanol extract derived from *E. tirucalli* demonstrated effective cytotoxicity against MCF-7 cells and MDA-MB231, with IC50 values of 15 and 30 µg/mL [229]. Consequently, there is a growing interest among researchers in utilizing these secondary metabolites for pharmaceutical applications.

#### 4.2.2. Alkaloids

Alkaloids are naturally occurring organic compounds characterized by the presence of at least one nitrogen atom [230]. They have been detected in nine plant species, including *E. horrida, E. trigona, E. clavarioides, E. gorgonis, E. enopla, E. bupleurifolia, E. ammak, E. polygona*, and *E. tirucalli*. The widespread identification of alkaloids in these selected species is unsurprising, given previous studies that have documented their presence in various species of the Euphorbiaceae family [231,232,233]. Prescription medicines derived from alkaloid-containing plants have been employed for many years, with their potent effects attributed to the presence of these compounds. Notable examples of alkaloids include morphine, utilized as an analgesic, and various other alkaloids such as vinblastine, quinine, atropine, nicotine, caffeine, ephedrine, and strychnine, all of which have medicinal applications [234]. Alkaloids exhibit several physiological effects in humans, including antibacterial, antimitotic, anti-inflammatory, local anesthetic, hypnotic, and antitumor activities [235]. The prevalence of alkaloids across these species highlights their shared pharmacological significance and their crucial role in medicinal applications.

#### 4.2.3. Saponins

Saponins, which are a class of glycosides, have been identified in seven plant species: *E. horrida*, *E. trigona*, *E. clavarioides*, *E. gorgonis*, *E. bupleurifolia*, *E. polygona*, and *E. ammak*. According to a report by Bigoniya and Rana [236], additional occurrences of saponins were noted in *E. neriifolia*, *E. paralias*, and *E. terracina* [237]. These compounds exhibit a range of pharmacological and medicinal properties, including modulation of cell membrane permeability, hemolytic activity, and antiviral, antifungal, anti-inflammatory, and antiallergic effects [238,239,240]. Moreover, saponins have shown promise in cancer therapeutics by diminishing cell invasiveness, inducing cell cycle arrest and apoptosis, and suppressing angiogenesis [241]. In conjunction with other antitumor agents, saponins have been utilized to enhance cytotoxicity in tumor treatment [242,243]. The consistent presence of saponins across these species underscores the genus’s shared chemical traits and its relevance in biomedical research.

A study by Xiao et al. [244] documented the inhibitory effects of triterpene saponins extracted from the root bark of *Aralia dasyphylla* Miq. on cancer cells in two different cell lines: KB and HeLa-S3. Another investigation identified eight steroidal saponins from *Allium porrum* L. capable of inhibiting WEHI 164 and J774 cells [245]. In 2001, Tran et al. assessed the antiproliferative activity of spirostanol- and furostanol-type saponins extracted from the roots and rhizomes of *Dracaena angustifolia* Roxb. against murine colon 26-L5 carcinoma, human HT-1080 fibrosarcoma, and B-16 BL6 melanoma cells [246]. Their study revealed that three of the tested compounds were highly effective in inhibiting the growth of HT-1080 fibrosarcoma cells. Furthermore, research conducted by Yokosuka et al. [247] demonstrated that two types of saponins, namely, ruscogenin glycoside and 26-glycosyloxyfurostanol saponin, exhibited cytostatic activity against HL-60 human leukemia cells. Moreover, researchers identified two novel triterpenoid saponins, glycosides A and B, in the aerial parts of *Glinus oppositifolius* L., which displayed efficacy against *Plasmodium falciparum*, a protozoan pathogen [248]. In another study by Iorizzi et al. [249], three new furostanol saponins and seven previously known saponins were extracted from the seeds of *Capsicum annuum* L. var. *acuminatum* Fingerh. However, the analysis indicated that these saponins had minimal to no effect on the growth of Gram-positive and Gram-negative bacteria.

#### 4.2.4. Tannins

Tannins, which are a class of polyphenols, have been identified in nine plant species: *E. horrida, E. trigona, E. clavarioides, E. gorgonis, E. bupleurifolia, E. enopla, E. polygona, E. arabica*, and *E. tirucalli*. These findings align with observations made by Aleksandrov et al. [2], who also reported elevated levels of tannins in *E. hirta*. According to Fraga-Corral et al. [250], topical application of tannins may aid in the removal of skin irritants, reduce inflammation, and demonstrate efficacy in the treatment of burns and wounds due to their anti-hemorrhagic and antiseptic properties. Furthermore, tannins have been extensively studied for their therapeutic potential across various diseases, attributed to their high antioxidant content, free radical scavenging ability, and antimicrobial and antiviral properties. Research has indicated their effectiveness in cancer chemotherapy [251,252,253,254,255]. Additionally, tannins have been reported to inhibit several strains of coronaviruses [256]. Their widespread presence across *Euphorbia* species points to a common defense and medicinal mechanism within the genus, illustrating how these plants share key chemical traits despite their varied uses.

#### 4.2.5. Glycosides

Glycosides, which are acetal derivatives of monosaccharides, were identified in 10 species of Euphorbia, including *E. horrida*, *E. trigona*, *E. enopla*, *E. clavarioides*, *E. gorgonis*, *E. bupleurifolia*, *E. polygona*, *E. arabica*, *E. tirucalli*, and *E. ammak*. Research conducted by Mshvildadze et al. [257] demonstrated that glycosides extracted from the bark of *Betula papyrifera* exhibited significant cytotoxic effects against lung carcinoma, colorectal adenocarcinoma, and normal skin fibroblasts. Additionally, Liu et al. [258] reported the cytotoxic activity of glycosides derived from *Antiaris toxicaria* against human lung cancer cells. The occurrence of glycosides reflects the genus’ shared capability to produce bioactive chemicals with significant therapeutic potential.

#### 4.2.6. Anthraquinones

Anthraquinones, which are phenolic compounds, were identified in four species of the *Euphorbia* genus, namely, *E. horrida*, *E. enopla*, *E. clavarioides*, and *E. Arabica* [259]. These anthraquinones are potent bioactive constituents found in various plant-based remedies and are known for their diverse health benefits, which include laxative, diuretic, estrogenic, and immunomodulatory properties. Furthermore, these compounds are utilized in cancer therapies and exhibit antibacterial, antiparasitic, insecticidal, fungicidal, and antiviral activities [259]. Although their presence is less widespread compared to other phytochemicals, their occurrence across multiple species underscores the diverse yet overlapping chemical profiles within the genus.

According to studies by Hanson [260] and Berdy [261], several *Euphorbia* species, including *E. bupleurifolia*, *E. gorgonis*, *E. horrida*, *E. polygona*, and *E. coerulescens*, contain alkaloids, saponins, and terpenoids. These compounds are believed to possess pharmacological properties, such as anticancer and antibacterial effects [262]. The findings indicate that a single species can be effective against multiple ailments, and many documented *Euphorbia* plants share similar secondary metabolites. Specifically, seven *Euphorbia* species have been reported to have cancer-fighting properties, including *E. trigona, E. tirucalli*, *E. clavarioides*, *E. gorgonis*, *E. bupleurifolia*, and *E. cooperi*. Five species, namely, *E. trigona*, *E. tirucalli*, *E. clavarioides*, *E. arabica*, and *E. bupleurifolia*, were noted for their use in wound healing and the treatment of warts. Additionally, *E. trigona*, *E. bupleurifolia*, and *E. gorgonis* were found to be effective in alleviating inflammation, while *E. arabica* and *E. cooperi* were utilized for stomach ailments, skin infections, and cracked heels. Notably, the seven species with anticancer properties were found to contain common secondary metabolites, including phytosterols, tannins, glycosides, triterpenoids, saponins, upholds, flavonoids, and alkaloids. It has been reported that the latex from *Euphorbia* species can exhibit high toxicity when ingested and may cause severe skin irritation [34,41].

#### 4.2.7. Terpenoids

Triterpenoids

Triterpenoids, which are a subclass of triterpenes, are found in most *Euphorbia* species. This study identified triterpenoids in 10 species, including *E. horrida*, *E. enopla*, *E. trigona*, *E. clavarioides*, *E. gorgonis*, *E. bupleurifolia*, *E. polygona*, *E. cooperi*, *E. arabica*, and *E. tirucalli*. These secondary metabolites have demonstrated various medicinal properties, including anticarcinogenic, antimalarial, antiulcer, anti-inflammatory, cytotoxic, anti-HIV, antiangiogenic, hepaticidal, antimicrobial, and antiviral effects [263,264,265]. Munro et al. [216] reported that the tetracyclic triterpene euphol, found in the latex of E. tirucalli, exhibits anticancer properties. Furthermore, Yasukawa et al. [103] revealed that *E. kansui* contains lanostane-type triterpenes capable of inhibiting inflammation induced by TPA, with euphol being the principal triterpene responsible for effectively preventing tumor promotion associated with TPA.

Euphol, a tetracyclic triterpene prevalent in the Euphorbiaceae family, is commonly found in *E. trigona, E. enopla*, *E. tirucalli*, *E. coerulescens*, and *E. ammak*. Popplewell et al. [72] discovered moderate activity of this compound against HepG2 cells. Additionally, Ahmed et al. [181] reported a substantial cytotoxic effect of euphol derived from *E. bothae* against MCF-7 cells. Silva et al. [102] evaluated the antitumor effects of euphol across various human cancer cell lines, finding it cytotoxic to several types, including esophageal squamous and pancreatic carcinoma cells. Moreover, Abdel-Sattar et al. [94] noted that euphol from *E. ammak* exhibited significant cytotoxicity against HeLa cells. In other investigations, euphol from *E. umbellata* demonstrated notable cytotoxic effects against HL-60, K-562, and B16F10 cells. Yasukawa et al. [103] also found that the topical application of euphol from *E. kansui* reduced the cancer-promoting effects of TPA by 90% in mouse skin. Akihisa et al. [147] identified euphol as a potent inhibitor of HIV-1 reverse transcriptase. Moreover, euphol has shown potential in mitigating inflammation and pain by inhibiting PGE2 and protein C kinase epsilon mediators [168]. However, data regarding the euphol content in *E. trigona, E. enopla, E. tirucalli, E. coerulescens,* and *E. ammak* remain undocumented.

Cycloartanol is a sterol lipid identified in several plant species, including *E. trigona*, *E. glareosa* Pall. Ex M. Bieb., *E. amygdaloides* L., and *E. palustris* L. [105]. A recent study by Salome-Abarca et al. [105] reported that cycloartenol extracted from these plants exhibited antifungal activity against *Botrytis cinerea*. Additionally, Barla et al. [110] identified cycloartanol in *E. helioscopia*, although they noted the absence of vasodepressor activity. Furthermore, Heliawati et al. [104] investigated the cytotoxic effects of cycloartanol extracted from the bark of *Corypha utan* Lamk on leukemia cells, revealing that cycloartanol was capable of inhibiting the growth of these cells. However, there is currently a lack of information regarding the effects of cycloartanol derived from *E. trigona*.

Lupeol, a triterpenoid, is found in various plants including *E. trigona*, *Tamarindus indica*, *Celastrus paniculatus*, *Zanthoxylum riedelianum*, *Allanblackia monticola*, *Himatanthus sucuuba*, *Leptadenia hastata*, *Crataeva nurvala*, *Bombax ceiba*, *Sebastiania adenophora*, *Aegle marmelos*, and *Emblica officinalis* [114,266,267,268,269]. You et al. [116] evaluated the effects of lupeol extracted from Bombax ceiba on various cell types, including HUVEC and SK-MEL-2, as well as B16-F10 melanoma cells. Their findings demonstrated that lupeol effectively inhibited the formation of HUVEC tubes by more than 80% at a concentration of 50 µg/mL, with no significant cytotoxic effects observed in the three cancer cell lines studied. Nguemfo et al. [270] researched the anti-inflammatory properties of lupeol from *Allanblackia monticola*, revealing that it significantly reduced paw edema by approximately 57.14% within just 30 min. Zhang et al. [111] investigated lupeol’s potential to inhibit the growth of liver cancer cells (HCCLM3) and discovered that it effectively impeded cell proliferation by suppressing the secretion of Brain-Derived Neurotrophic Factor, phosphatidylinositol 3-kinase, and Wnt signaling pathways. Borgati et al. [113] assessed the efficacy of lupeol from *Parahancornia fasciculata* against chloroquine-resistant W2 clones to treat malaria, finding only limited activity. Additional investigations have examined the antidiabetic properties of lupeol derived from *Solanum xanthocarpum* [271], which demonstrated its capability to impede the progression of diabetes by reducing glucose levels, lowering nitric oxide, increasing serum insulin, and enhancing antioxidant levels. Sudhakar et al. [117] explored the effects of lupeol obtained from *Crataeva nurvala* on cholesterol levels, concluding that this compound can diminish oxidative stress and inflammatory cytokines, leading to decreased nitric oxide production and ultimately resulting in lower cholesterol levels. However, data concerning the lupeol composition of *E. trigona* remains undocumented.

α-amyrin, a triterpenoid, is present in several plant species, including *E. trigona*, *E. tirucalli*, *E. aphylla* Brouss., *E. schimperi* C. Presl, and *E. hirta* [123,125,272]. Abdel-Monem et al. [123] reported that α-amyrin extracted from *E. schimperi* C. Presl exhibited moderate cytotoxicity, inducing a 40–50% reduction in viability of U251 and MCF-7 cell lines at a concentration of 10 µg/mL. In contrast, other studies indicated negligible cytotoxicity against the HCT 116 cell line [272]. Jabeen et al. [120] investigated the antifungal properties of α-amyrin derived from *Melia azedarach* L. in vitro, finding it effective against *Ascochyta rabiei*, with a minimum inhibitory concentration (MIC) of 0.0156 mg/mL. However, there is currently no available data regarding the effects of α-amyrin from *E. trigona* and *E. tirucalli*.

β-amyrin, another triterpenoid, has been identified in *E. trigona* and *E. hirta*, demonstrating anticancer properties against Hep-G2 cancer cells [118]. This compound also exhibited a weak cytotoxic effect against the NTUB1, A549, and HL-60 cell lines [119]. Ragasa and Cornelio [272] reported insignificant cytotoxicity against the HCT 116 cell line. Additionally, Vazquez et al. [125] showed that β-amyrin exhibited anti-inflammatory activity in a murine ear model. Shih et al. [121] found that β-amyrin from *E. hirta* could ameliorate arthritis inflammation by inhibiting nitric oxide pathways. Lin et al. [119] reported that the combination of β-amyrin with cisplatin resulted in the generation of reactive oxygen species, triggering cell cycle arrest and apoptosis in NTUB1 cells. Nonetheless, there is currently no documented information on the β-amyrin present in *E. trigona*.

Betulinic acid, a triterpenoid, has been identified in several plant species, including *E. trigona, Tetracarpidium conophorum* seeds, *Uapaca paludosa*, *Manniophyton fulvum* (Euphorbiaceae), and *Agathosma betulina* [273,274,275,276]. A study by Zhang et al. [111] demonstrated that betulinic acid has the potential to inhibit the growth of over 20 different cancer cell lines. Research conducted by Damle et al. [128] revealed that betulinic acid exhibited cytotoxic effects on MCF-7 breast cancer cells, with an IC50 value of 13.5 mg/mL. Furthermore, this compound was shown to affect the specificity of protein transcription factors that are typically overexpressed in cancer cells relative to normal cells [130].

In another study by Mbeunkeu et al. [274], it was found that betulinic acid derived from *Manniophyton fulvum* had a significant impact on HeLa cells, resulting in an extremely low cell viability rate of just 4%. Research conducted by Foo et al. [127] discovered that the dichloromethane (DCM) fraction of *Dillenia suffruticosa* induces cell cycle arrest and apoptosis in MCF-7 cells through the p35/p21 signaling pathway. Additionally, it was shown to inhibit the growth and colony formation capabilities of all tested human melanoma cell lines [135]. According to Oriakhi et al. [273], betulinic acid demonstrated a 54% inhibition of HepG2 liver cancer cells and exhibited hepatoprotective activity, with a calculated binding energy of −11.2 kcal/mol to Hepatitis B virus DNA.

Other studies have reported the antidiabetic effects of betulinic acid through the inhibition of pancreatic α-amylase [131,277]. Bernard et al. [134] also noted that betulinic acid possesses anti-inflammatory properties, specifically by inhibiting phospholipase A2. Mukherjee et al. [278] reported that betulinic acid significantly reduced rat paw edema induced by carrageenan and serotonin. Additionally, it was found that *Hypericum hircinum* L. contains betulinic acid, which can inhibit the replication of HIV-1 by interfering with reverse transcriptase-associated DNA polymerase; this is accomplished by either hindering HIV fusion or disrupting a specific stage of its maturation process [129,133]. However, as of now, data on the betulinic acid content of *E. trigona* remains undocumented.

Taraxerol, a triterpenoid, has been previously isolated from various plant sources, including *E. trigona*, *E. neriifolia* Linn, *Artemisia roxburghiana*, *Taraxacum japonicum*, and the fruits of *Dregea volubilis* [136,139,141,279]. Taraxerol isolated from *Vepris punctate* showed limited activity against A2780 cells [280]. Studies by Cao et al. [281] also showed minimal inhibition of cell growth of A2780 cells at the highest tested concentration. Taraxerol from *Conyza canadensis* was found to have a maximum antiproliferative effect against A431. However, taraxerol displayed no activity against HeLa, MCF-7, and MRC-5 [282]. In addition, it was found that taraxerol effectively hindered the growth of AGS cells by causing G(2)/M arrest and stimulating cell apoptosis [138]. According to Takasaki et al. [141], taraxerol is highly efficacious in preventing tumors in mice during two-stage carcinogenesis tests. In addition, Singh et al. [140] reported that taraxerol has anti-inflammatory properties, reducing paw edema by 48.61%. Furthermore, it was also observed that taraxerol exhibited moderate antimicrobial activity against certain Gram-negative and Gram-positive bacteria [140]. According to a study conducted by Min et al. [283], taraxerol found in *Styrax japonica* showed low effectiveness in scavenging free radicals based on the DPPH assay. Sangeetha et al. [284] investigated the use of taraxerol from *Mangifera indica* as an antidiabetic agent. According to the findings, the compound has dual activity as a glucose activator and stimulator of glycogen, making it a potential treatment for type 2 diabetes. However, there is currently no documented information available on the taraxerol of *E. trigona*.

Taraxerol acetate, a triterpenoid, has been previously isolated from *E. trigona*, *E. pubescens*, and *Artemisia roxburghiana* [285]. Studies indicate that taraxerol acetate from *A. roxburghiana* can significantly reduce edema in mice induced by carrageenan [136]. Moreover, Rehman et al. [137] demonstrated that taraxerol acetate can inhibit cyclooxygenase enzymes 1 and 2. However, to date, there is no documented data on the presence and effects of taraxerol acetate derived from *E. trigona*.

Friedelin, another triterpenoid, has been identified in species such as *E. trigona*, *E. tortilis* Rottler, *Mangifera indica*, and *Lentinus edodes* [153,286,287]. Previous research has revealed that, when evaluated for antibacterial properties against various Gram-positive and Gram-negative bacteria, friedelin displayed weak activity, with a minimum inhibitory concentration (MIC) exceeding 250 μg/mL [153]. However, it was found that friedelin from *Mangifera indica* exhibited anti-colorectal cancer activity [154]. Despite the lack of studies on friedelin sourced from *E. trigona*, it remains a promising subject for future research.

Friedelan-3-β-ol, a triterpenoid, has also been isolated from *E. trigona* and *Mangifera indica*. This compound has demonstrated significant inhibition of thymidylate synthase, thereby indicating its potential as an anti-colorectal agent [154]. Nonetheless, documented data on friedelan-3-β-ol derived from *E. trigona* is currently unavailable.

3β-Friedelinol, another triterpenoid, has been identified in *E. trigona, E. vajravelui*, *E. kamerunica*, and *Maytenus robusta* [157,158]. Sousa et al. [158] found that 3β-friedelinol from *Maytenus robusta* exhibited cytotoxic effects against 4T1 cells. Simultaneously, Ogunnusi et al. [157] investigated the antibacterial activity of 3β-friedelinol from *E. kamerunica*, discovering its inhibitory capacity against certain bacteria. However, current data concerning 3β-friedelinol from *E. trigona* remains lacking.

#### 4.2.8. Phytosterols

Phytosterols, which are a class of sterols, have been identified in nine plant species, namely, *E. horrida*, *E. trigona*, *E. clavarioides*, *E. gorgonis*, *E. enopla*, *E. bupleurifolia*, *E. cooperi*, *E. polygona,* and *E. arabica*. Research indicates that phytosterols possess bioactive properties that confer several health benefits, including reducing inflammation, preventing oxidative stress, anti-cancer effects, and lowering cholesterol levels [288,289]. A combination of phytosterols has been shown to inhibit tumor development in various cancers, including cholangiocarcinoma and breast cancer, at physiological doses [290,291].

Cycloartenol is a specific phytosterol compound found in certain species, including *E. trigona, E. nicaeensis*, *E. broteri*, *E. macrosteigia*, and *E. boetica* (NHI). It serves as a precursor to various sterol compounds and exhibits numerous pharmacological benefits, such as anti-inflammatory, antitumor, antioxidant, antibacterial, and anti-Alzheimer’s activities [108,109]. A study conducted by Niu et al. [107] explored the potential anticancer properties of cycloartenol in glioma U87 cells. The findings revealed that cycloartenol effectively inhibited both the growth and colony-forming ability of glioma U87 cells, likely through the induction of Sub-G1 cell cycle arrest and apoptosis, which contributed to its antiproliferative effects. Additionally, Zare et al. [109] identified cycloartenol as possessing anticancer, analgesic, and bactericidal properties. Furthermore, Sawale et al. [106] demonstrated that cycloartenol extracted from *E. neriifolia* exhibited antioxidant activity ranging from 34.56% to 72.87% at concentrations of 10, 20, 40, 60, 80, and 100 μg/mL. However, data regarding the cycloartenol content of *E. trigona* remains undocumented.

β-sitosterol, a phytosterol, has been previously isolated from species such as *E. trigona*, *E. abyssinica*, *Pinellia ternate*, and *Nyctanthes arbortristis* [142,143,145]. Investigations into its effects on the proliferation of Caski and HeLa cells have assessed its potential antiproliferative and anticancer properties. The results indicated that β-sitosterol significantly reduced the expression of antigens responsible for cell proliferation in both cervical carcinoma cell lines [142]. In a 2015 study, Cheng et al. reported that increased levels of P53, alongside decreased levels of HPVE6 viral oncogenes, were associated with the anticancer activity of β-sitosterol in Caski and HeLa cells [142,153]. Previous research has also demonstrated that β-sitosterol derived from *Nyctanthes arbortristis* leaves exhibits considerable anti-inflammatory effects, as evidenced by its impact on paw edema in rats [143]. Moreover, β-sitosterol isolated from *E. abyssinica* has shown significant antimicrobial activity against Candida albicans [145]. Additionally, β-sitosterol sourced from *E. hirta* has been reported to prevent inflammation induced by TPA [125]. However, there is currently no documented data on the β-sitosterol of *E. trigona*.

24-Methylenecycloartanol, another sterol, has been previously isolated from *E. hirta*, *E. heteradena*, *E. trigona*, *E. broteri*, *E. palustris*, *Moroccan propolis*, and *E. aleppica* [155,292,293,294]. According to the findings of Krstić et al. [155], 24-Methylenecycloartanol from *E. palustris* demonstrated a stronger antifungal effect against *Fusarium sporotrichioides* and *Alternaria alternata*. Previous studies indicated that 24-Methylenecycloartanol derived from *E. hirta* can mitigate inflammation triggered by TPA [125]. However, there is currently no available data on the effects of 24-Methylenecycloartanol from *E. trigona*.

Tirucallol, a tetracyclic triterpene, has been identified in the latex of *E. enopla* and *E. lacteal*. This compound exhibits anti-inflammatory properties. Research has shown that in murine models, tirucallol led to a reduction in ear edema and inhibited nitrate production in stimulated macrophages [146,295]. Furthermore, it has been found to inhibit HIV-1 reverse transcriptase [147]. Nevertheless, there is currently no available data regarding the presence or effects of tirucallol from *E. enopla*.

Obtusifoliol, another type of sterol, is present in several plant species, including *E. cooperi*, *E. bothae*, *E. chamaesyce*, and *E. sogdiana* [72,296]. A study conducted by Aghaei et al. [195] demonstrated that obtusifoliol derived from *E. sogdiana* exhibited reduced cytotoxicity against MCF-7 cells, with an IC50 value of 29.33 ± 1.52 μM. Similarly, Ahmed [28] reported weak cytotoxicity of obtusifoliol from *E. cooperi* against MCF-7 cells.

Glutinol, a triterpenoid, has been isolated from *E. cooperi*, *E. ammak*, *E. chamaesyce*, and *Scoparia dulcis* [94,297]. This compound has demonstrated a notable cytotoxic effect against HeLa cells [94]. A study conducted by Ding et al. [192] investigated the potential toxicity of glutinol derived from *Acer mandshuricum* on various cell types, including leukemia, ovarian, lung, and human colon cells. The findings revealed significant inhibition of the cell lines, with GI_50_ values ranging from 11.6 to 16.0. Another research study by Chen and Li [191] also reported the antiproliferative effects of glutinol on ovarian cells. However, to date, there is insufficient data on the effects of glutinol from *E. cooperi* and *E. ammak*.

Triterpene euphol, a steroidal alcohol, is present in plants such as *E. tirucalli*, *E. umbellata*, and *Synadenium grantii* [298,299]. Research indicates that triterpene euphol, the primary compound found in *E. umbellata* and *E. tirucalli*, shows potential as a complementary cancer treatment [52,298]. Lin et al. [76] reported that triterpene euphol exhibited moderate cytotoxic activity against gastric adenocarcinoma cells. Silva et al. [102] also noted its cytotoxic effects on glioblastoma cells. A study by de Oliveira et al. [299] examined the antitumor effects of triterpene euphol from *Synadenium grantii*, finding that the compound did not demonstrate antitumor activity against B16F10 melanoma cells. Furthermore, a study by Dutra et al. [168] explored the potential of triterpene euphol from *E. tirucalli* in preventing and treating inflammation in murine colons. Their findings indicated that this compound was highly effective in reducing pro-inflammatory mediators in vitro.

24-Methylene cycloartenol, a sterol, has previously been extracted from *E. tirucalli* and *E. neriifolia*. The pharmacological activities of the entire plant were assessed, rather than focusing specifically on the compound itself. 24-Methylene, isolated from *E. hirta*, has been demonstrated to possess significant anti-inflammatory properties when used to treat ear inflammation induced by acetate anti-inflammatory agents [125]. Currently, information regarding the anticancer properties of 24-methylene cycloartenol from *E. tirucalli* is lacking.

Ingenol triacetate, a diterpene, has demonstrated antimicrobial activity against various pathogens responsible for infectious diseases [188]. Moreover, ingenol triacetate has been identified as a nontumor promoter [188]. However, a separate study by Tilabi and Upadhyay [189] revealed that the topical application of ingenol triacetate to female NMRI mice resulted in a significant occurrence of lung adenoma. Currently, there is a lack of information regarding the anticancer properties of ingenol triacetate derived from *E. tirucalli.*

Terpenic alcohol, a terpene isolated from *E. tirucalli*, has shown antibacterial properties against *Staphylococcus aureus*, attributed to its capacity to disrupt cellular membranes [148,300]. However, the study did not directly investigate terpenic alcohol [149]. Further research is warranted to explore this compound, particularly its isolated form from *E. tirucalli*.

Taraxasterol, a phytosterol extracted from *E. tirucalli*, *Carthamus tinctorius*, *Chrysanthemum morifolium*, and *Helianthus annuus* [178,301], has been shown to effectively reduce ear inflammation induced by TPA in mice and prevent tumor growth in mouse skin. In two-stage carcinogenesis tests, taraxasterol exhibited robust antitumor activity when applied to mouse skin. Furthermore, research indicated that taraxasterol has the potential to suppress spontaneous mammary tumors in C3H/OuJ mice. It should be noted that the taraxasterol tested was not derived from *Euphorbia* species [178]. Additional pharmacological investigations are needed on *Euphorbia* species, with a specific focus on *E. tirucalli*, as recommended by Ovesnâ et al. [178].

Stigmasterol, a member of the sterol class, has been extracted from *E. ammak* and *Butea monosperma* [193]. In a separate study conducted by Abdel-Sattar et al. [94], the cytotoxic activity of stigmasterol derived from *E. ammak* was investigated. The study demonstrated that the compound effectively targets HeLa cells. In 2009, Panda and colleagues examined the effects of stigmasterol from *B. monosperma* [193], revealing that stigmasterol possesses the ability to inhibit thyroid function and reduce blood glucose levels. Additionally, their research indicated that stigmasterol has the potential to mitigate liver damage caused by oxidative stress by decreasing harmful lipid peroxidation levels and enhancing the activity of protective enzymes. Further studies have confirmed that this compound exhibits cytotoxicity against MCF-7 breast cancer cells [24,28]. Although some diterpenoids from *Euphorbia* (such as phorbol esters) are known to be toxic, recent research highlights that certain compounds isolated from this plant exhibit significant bioactivity [26,27].

#### 4.2.9. Other Terpenoids

12-Deoxyphorbol-13-isobutyrate-20-acetate is a phorbol ester found in various plants, including *E. ledienii, E. coerulescens*, *E. tirucalli*, *E*. *triangularis*, *E*. *resinifera*, and *E*. *bothae.* Research conducted by Ourhzif et al. [161] demonstrated that 12-Deoxyphorbol-13-isobutyrate-20-acetate extracted from *E. resinifera* latex exhibited cytotoxic properties, inhibiting the growth of *Aspergillus carbonarius*. However, there is currently a lack of data regarding the occurrence and effects of 12-Deoxyphorbol-13-isobutyrate-20-acetate found in *E. ledienii, E. coerulescens*, and *E. tirucalli*.

Phorbol, a diterpenoid, has been extracted from *E. ledienii* and *Croton tiglium*, both of which belong to the Euphorbiaceae family. It has also been identified in *E. tirucalli*, as reported by Fürstenberger and Hecker [7]. Some studies indicate that phorbol may act as an irritant and promote tumorigenesis; conversely, other research suggests that it can induce apoptosis in tumor cells via the activation of protein kinase C [162]. Nonetheless, information regarding the presence and effects of phorbol in *E. ledienii* and *E. tirucalli* remains limited.

12-Deoxyphorbol ester, another diterpenoid, has been isolated from *E. ledienii* and *E. grandicornis*. A study by Zayed et al. [163] reported that this compound activates protein kinase C and does not promote tumor proliferation. Previous investigations have shown that this activation may exhibit antiproliferative effects against certain cancer cell types. For instance, research by Shen et al. [164] indicated that 12-Deoxyphorbol ester inhibited the proliferation of myeloid leukemia cells. Additional studies demonstrated that 12-Deoxyphorbol ester caused increased cell death in breast cancer cells relative to normal breast epithelial cells [165]. Further, Tsai et al. [166] reported that 12-Deoxyphorbol ester has the ability to suppress growth and induce apoptosis in human lung cancer cells through the activation of the PKC-δ/PKD/ERK signaling pathway. However, there is currently no information available on the effects of this compound derived from *E. ledienii*.

12-Deoxy-16-hydroxy-phorbol is a constituent of DHPB that has been identified in the latex of several plant species, including *E. ledienii*, *E*. *poisonii* Pax, *E. cooperi*, *Jatropha curcas*, and *Jatropha gossypiifolia* [302,303]. This compound has been shown to stimulate ornithine decarboxylase activity in mouse skin, inhibit the binding of [3H]-12-O-tetradecanoylphorbol-13-acetate to phorbol ester receptors, and activate protein kinase C in vitro [167]. However, experimental data regarding the effects of 12-Deoxy-16-hydroxy-phorbol specifically from *E. ledienii* and *E. cooperi* remain unreported.

16-Hydroxy-12-desoxyphorbol, a diterpenoid, has been isolated from *E. cooperi*, *E. bothae*, *E. triangularis, E. ingens*, *Croton rhanmmifolius*, and *E. rowlandii* [194,304]. Dinala et al. [194] documented that this compound exhibited activity against the HCC70 and MCF-7 cell lines, with EC50 values of 0.592 µM and 1.003 µM, respectively. In contrast, Gschwendt and Hecker [97] classified the compound as a tumor promoter. Nevertheless, further investigation into the properties of 16-Hydroxy-12-desoxyphorbol from *E. cooperi* is still warranted.

12-Deoxyphorbol-13-isobutyrate-16-angelate-20-acetate, a member of the diterpene class, has been identified in *E. cooperi* and *E. bothae* [72]. Morsi [28] reported that 12-Deoxyphorbol-13-isobutyrate-16-angelate-20-acetate derived from *E. cooperi* demonstrated significant cytotoxicity against MCF-7 cells.

12-Deoxy phorbol esters, belonging to the phorbol ester class, have been previously isolated from *E. tirucalli*, *E. triangular*, *E. resinifera*, and *Excoecaria bicolor* [97,163,305]. These compounds have been reported to be carcinogenic and act as tumor promoters [97,175]. Research conducted by Driedger and Blumberg [174] indicated that exposure to 12-Deoxy phorbol esters led to inflammation in the ears of mice.

Ingenol, a diterpenoid, has been identified in both *E. tirucalli* and *E. sikkimensis*. In 2019, Silva and colleagues investigated the effects of ingenol, derived from *E. tirucalli*, on various human cancer cell lines [102]. The results demonstrated that the compound exhibited a range of effectiveness, from weak to potent, against different cell lines. Additionally, other studies have shown that ingenol effectively inhibited the replication of HIV-1 subtypes B and C in both MT-4 cells and human peripheral blood mononuclear cells (PBMCs) [152].

Glut-5-en-3-β-ol, a triterpenoid, has been identified in *E. tirucalli* and *E. pseudocactus* Berger [176]. Abdel-Monem and Abdelrahman [176] conducted a study to evaluate the antimicrobial activity of Glut-5-en-3-β-ol against various microorganisms. However, the results indicated that Glut-5-en-3-β-ol did not demonstrate any activity against the tested bacterial strain.

#### 4.2.10. Phenolic Compounds

Gallic acid, a phenolic compound derived from *E. cooperi*, demonstrates significant hepatoprotective capabilities, particularly in safeguarding the liver from damage induced by paracetamol. It also has antioxidant properties that can improve the levels of serum alanine aminotransferase, aspartate aminotransferase, alkaline phosphatase, and bilirubin antioxidant [181].

Bervifolin carboxylic acid, a tannin extracted from *E. cooperi*, exhibits a robust hepatoprotective effect. This compound also serves as an antioxidant, effectively decreasing serum levels of alanine aminotransferase, aspartate aminotransferase, alkaline phosphatase, and bilirubin in rats subjected to paracetamol-induced hepatotoxicity [181].

3, 3′-Dimethoxy ellagic acid, another tannin obtained from *E. cooperi*, reveals significant hepatoprotective and antioxidant properties. Notably, it reduces serum levels of alanine aminotransferase, aspartate aminotransferase, alkaline phosphatase, and bilirubin in rats experiencing paracetamol-induced hepatotoxicity. Furthermore, this compound exhibits moderate cytotoxic activity against HepG2 cells [181].

Ellagic acid, a tannin sourced from *E. cooperi*, has been shown to confer multiple benefits for liver health in rodent models. In various studies, it has demonstrated protective effects on the liver, inhibited cellular proliferation, and exhibited antioxidant activity [28,181]. Moreover, it reduces the levels of certain enzymes and bilirubin in the blood, which are commonly elevated in cases of paracetamol-induced liver damage [28,181].

Kaempferol and Kampferol-3-O-ß-D-rutinoside, both flavonoids derived from *E. cooperi*, have been identified as effective agents in liver protection. These compounds inhibit excessive cell proliferation and lower the serum levels of specific enzymes and bilirubin, all of which are associated with paracetamol-induced liver damage [28,181].

1-O-Galloyl-3,6-hexahydroxydiphenyl-β-D-glucopyranoside (commonly known as corilagin) is a tannin extracted from several plant species, including *E. cooperi*, *E. prostrata*, *Phyllanthus amarus* L., *Phyllanthus niruri* L., and *E. longana* Lam (family Euphorbiaceae) [197,200,202,306,307]. In 2013, Ming and colleagues investigated the potential antitumor effects of corilagin derived from *Phyllanthus niruri* L. on three cell types: Chang liver cells, SMMC7721, and Bel7402 cells [198]. Their findings demonstrated that corilagin exhibited moderate efficacy against SMMC7721 and Bel7402 cells, but it showed only weak activity against Chang liver cells. Research conducted by Bai et al. [199] revealed that corilagin extracted from *Dimocarpus longan* Lour had cytotoxic effects on A549 cells. Additionally, Tong et al. [196] reported that corilagin from *Phyllanthus urinaria* induced apoptosis through the activation of reactive oxygen species and autophagy. This process involved the suppression of the Akt/mTOR/p70S6K signaling pathway, resulting in an increased formation of autophagic vacuoles and the conversion of LC3-I to LC3-II. A report by Morsi [28] indicated that corilagin exhibited significant toxicity against MCF-7 cells. Kolodziej et al. [200] investigated the effects of corilagin from *Phyllanthus amarus* L. on various inflammation-related genes, including iNOS. Their results indicated that corilagin increased the mRNA expression levels of iNOS and cytokines in parasitized cells. In a separate study in 2013, Jin and associates evaluated the impact of corilagin on liver protection and inflammation reduction using animal models [201]. Their findings suggested that corilagin inhibited the NF-κB signaling pathway, which led to an increase in superoxide dismutase levels, a critical defense mechanism against superoxide radical toxicity, and nitric oxide, a key player in liver metabolism. Previous studies have shown that corilagin sourced from *E. longana* Lam can lower blood pressure by reducing plasma noradrenaline release and inducing direct vasorelaxation [202]. Yang et al. [308] explored the effects of corilagin from *Terminalia bellerica* Roxb on HepG2 cells, specifically assessing its potential for diabetes treatment. Their findings revealed that corilagin enhanced PPARγ signaling, resulting in increased adipogenesis. According to research conducted by Latté and Kolodziej [309], published in *Zeitschrift für Naturforschung*, corilagin demonstrated a strong antifungal effect against *Candida glabrata*. Notka et al. [310] reported that corilagin from *Phyllanthus amarus* inhibited HIV-1 replication.

Tri-methyl ellagic acid, another tannin, has been previously isolated from *E. tirucalli* and *E. sorori*. In 2008, Zhang et al. [151] assessed the antibacterial effect of tri-methyl ellagic acid on various bacterial strains. The results indicated that this compound exhibited a moderate inhibitory effect on *Bacillus subtilis* and *Staphylococcus aureus*, but it showed no activity against *Escherichia coli*. Currently, there is no available data on the anticancer properties of the tri-methyl ellagic acid derived from *E. tirucalli*.

3′-Di-O-methylellagic acid, a phenolic compound, has been isolated from *E. tirucalli*, *E. lunulata*, and *E. schimperiana* [311]. Research has evaluated the cytotoxicity of this compound extracted from *E. schimperiana* against four human cancer cell lines. The results demonstrated that 3,3′-Di-O-methylellagic acid exhibited promising cytotoxicity against PC3 cells [184,312], with an IC50 value of 5.5 μg/mL, indicating potential anticancer activity [184]. Additionally, it has been found that 3′-Di-O-methylellagic acid from *E. schimperiana* displayed notable antibacterial properties, as confirmed by the same study. Aljubiri et al. [184] also validated the anti-inflammatory activity of 3′-Di-O-methylellagic acid isolated from *E. lunulata*. Guo et al. [185] discovered that *E. hylonoma* contains 3′-Di-O-methylellagic acid, which possesses strong antioxidant properties. Furthermore, the isolate from *E. thymifolia* showed significant potential as an antimicrobial agent.

#### 4.2.11. Fatty Acids

Laurate, a fatty acid, has been identified in *E. coerulescens*. It exhibits limited antimicrobial activity against beneficial lactic acid bacteria while demonstrating strong antimicrobial effects against harmful *Bacteroides* and *Clostridium* species [190,313]. However, there is currently a lack of available data regarding the effects of laurate derived from *E. coerulescens*.

#### 4.2.12. Miscellaneous

Diterpene esters, a class of terpenes, have been extracted from the latex of several *Euphorbia* species, including *E. horrida*, *E. periplus*, *E. tirucalli*, and *E. cauducifolia* [7,314]. These compounds are known to be skin irritants and tumor promoters [7,314]. In contrast, studies by Kedei et al. [315] reported that diterpene esters possess anticancer properties by activating protein kinase C, a mechanism previously shown to induce an anticancer effect on cells. Research conducted by Ogbourne et al. [316] indicated that the topical application of high doses of diterpene esters successfully cured skin cancer in mice via this protein kinase C pathway. Another study by Hampson et al. [317] found that administering a significantly lower dose of diterpene esters resulted in cell death through a protein kinase C-dependent mechanism in leukemia cells. Furthermore, Nothias-Scaglia et al. [318] reported that diterpene esters can inhibit HIV replication at the nanomolar level. Nevertheless, there is currently no available data on the effects of diterpene esters derived from *E. horrida* and *E. tirucalli*.

Among the 15 studied *Euphorbia* species, several compounds remain unexplored. These include Ingol-7,8,12-acetate and ditiglate from *E. ledienii*, as well as 12-Deoxyphorbol-13-(2-methyl butyrate)-20-acetate. One compound identified from *E. horrida* was characterized as 17-Hydroxyingenol-17-benzoate-20-angelate. Five compounds were identified from *E. coerulescens*: Angelate acetate isobutyrate, fatty acids, acetate laurate, α-methyl butyrate, and heptanoate. Additionally, five compounds were identified from *E. cooperi*: euphorbilactone, norsesquiterpenoid, arachiside A, 16-angeloyloxy-13α-isobutanoyloxy-4β,9α,20-trihydroxytiglia-1,5-diene-3,7-dione, and 20-acetoxy-16-angeloyloxy-13α-isobutanoyloxy-4β,9α,20-tetrahydroxytiglia-1,5-diene-3-one. Finally, ten compounds were identified from *E. tirucalli*: euphorbol hexacosonate, 12-deoxy-4β-hydroxyphorbol-13-phenyl acetate-20-acetate, 12,20-dideoxyphorbol-13-isobutyrate, tirucalicine, isoeuphorol, ketone euphorone, euphorbin A, euphorbin B, tirucallin A, and tirucallin B.

### 4.3. In Silico Evaluation of Selected Phytochemicals

*Euphorbia* species have been extensively documented for their various biological activities and pharmacological potential, highlighting their promise as anticancer agents. Certain extracts and isolates derived from these plants have been reported to exhibit cytotoxic effects, as outlined in this study. However, the specific phytochemical constituents responsible for these cytotoxic effects and other biological activities remain largely unexplored. In silico techniques, which facilitate resource maximization and search space minimization, may provide valuable insights into the biological activities, pharmacokinetics, pharmacodynamics, and pharmacotherapeutics of phytochemicals sourced from *Euphorbia* species. This could serve as a fundamental resource for researchers aiming to design more targeted experiments and manage future studies concerning the medicinal benefits of underexplored and underutilized *Euphorbia* species.

#### 4.3.1. Cell Line Cytotoxicity Tendency of Selected Compounds

Promising compounds derived from *Euphorbia*, as detailed in Table S1, were evaluated in silico for their cytotoxicity and anticancer potential using the Cell Line Cytotoxicity Predictor (CLCPred) web server [11]. Among the 74 compounds subjected to evaluation, 40 were prioritized based on the availability of pertinent information and their high cytotoxic activity. The compounds predicted to demonstrate substantial cytotoxic activity exhibited significant anticancer potential when the probability cutoff was set such that P(a)ctive > P(i)nactive and Pa > 0.50. These compounds displayed notable cytotoxic tendencies against multiple human cancer cell lines and tumor types. Among these, several *Euphorbia*-derived compounds (EDCs) such as euphol, cycloartenol, lupeol, α-amyrin, betulinic acid, 24-Methylene-cycloartanol, 12-Deoxyphorbol 13-tiglate 20-acetate, Ingol 7,8,12-acetate, diterpene glycoside, tirucallol, Isobutyl angelate, and Kaempferol-3-O-rutinoside were found to possess high cytotoxic probabilities against three or more cell lines (refer to Table S1). Given the vast potential of plant-based natural products in drug discovery and the role of terpenoids in the development of anticancer compounds, the terpenoids derived from *Euphorbia* species can be further explored to yield reliable, economically viable, and environmentally safe bioactive molecules. Collectively, these terpenoids exhibit cytotoxic tendencies against a variety of cancer cell lines, including stomach carcinoma, kidney carcinoma, skin melanoma, liver hepatoblastoma, lung carcinoma, brain glioma, colon adenocarcinoma, and thyroid carcinoma (see Table S1).

#### 4.3.2. Physicochemical and Drug-like Properties

The physicochemical parameters and drug-likeness of the endocrine-disrupting chemicals (EDCs) were computed based on their chemical structures using the SwissADME platform, as detailed in Table S2 (physicochemical properties; drug-like properties). As shown in Tables S1 and S2, most of the compounds exhibit favorable physicochemical characteristics. All EDCs, with the exception of C8, C9, and C12, possess molecular weights of less than 500 Daltons. Additional desirable physicochemical attributes observed in several compounds include lipophilicity (LogP), solubility, Polar Surface Area (PSA), and the number of Hydrogen Bond Donors and Acceptors. Moderate molecular weight is favorable for drug-likeness, as excessively large or small molecules may encounter challenges related to absorption, distribution, metabolism, and excretion (ADME) properties.

Drug-likeness refers to the set of characteristics that enhance a molecule’s potential to become an effective and safe drug candidate. Understanding these physicochemical properties is crucial for predicting a molecule’s behavior in biological systems and its viability as a drug. Many of these properties are fundamental to drug development, and any violation may lead to delays or failures in bringing a drug candidate to the market. Notably, all compounds, except for C9 and C12, demonstrate desirable Lipinski and Veber criteria, as well as favorable synthetic accessibility. Lipinski’s Rule of Five (RO5) serves as a guideline for assessing drug-likeness based on physicochemical properties. It suggests that the following criteria must be met for a compound to exhibit good oral bioavailability: molecular weight < 500 Daltons, LogP < 5, hydrogen bond donors < 5, and hydrogen bond acceptors < 10 [319,320]. Although adherence to the RO5 is not a strict prerequisite for a compound to be classified as a drug, it is a valuable guideline in early-stage drug discovery for prioritizing compounds with favorable characteristics.

#### 4.3.3. ADMET Properties of Top Compounds

The fate and behavior of a drug or chemical compound within the human body are significantly influenced by absorption, distribution, metabolism, excretion, and toxicity (ADMET) factors. Understanding these properties is crucial in drug development to ensure both efficacy and safety. Computational tools play a pivotal role in predicting these properties, and in this study, we utilized the pKCSM webserver [13] to predict the ADMET properties of the top compounds, as detailed in Table S2.

The predictions regarding the intestinal absorption of *Euphorbia*-derived terpenoids in humans, based on computed molecular descriptors, indicate that all the compounds exhibit good human intestinal absorption (%Absorbed). Additionally, most of these compounds are predicted as P-glycoprotein inhibitors (PGIs). PGIs interfere with glycoprotein activity, preventing the pumping of drug compounds out of cells. This inhibition can lead to the maintenance of high intracellular drug concentration, potentially improving therapeutic efficacy. Notably, several FDA-approved drugs and natural compounds are recognized as PGIs [321].

The human volume of distribution (VDss) is a pivotal parameter for determining the human dose. While some *Euphorbia*-derived compounds feature low VDss, the majority exhibit a low plasma binding tendency. A drug with strong plasma protein binding may struggle to dissociate from plasma proteins and bind to target receptors, even if present in the bloodstream. Thus, a candidate with very high plasma levels due to very low VDss may appear promising, but it is essential to examine the magnitude of the pharmacological effect to avoid potential misinterpretations [322].

The computed interactions of the compounds with liver phase I drug metabolism, using various cytochrome P450 descriptors, reveal that most of the top compounds do not exhibit inhibitory tendencies against various cytochrome P450s. This suggests that these compounds may not adversely affect phase I drug metabolism in the liver.

Organic cation transporter 2 (OCT2) mediates the initial step in the renal secretion of organic cations. However, several compounds exhibit a high clearance rate tendency, and all are predicted to be non-substrates of OCT2.

Computed molecular descriptors assessing the toxicity tendency of the compounds indicate that the majority may not be toxic, as indicated by AMES toxicity and maximum dose (human) predictions. hERG I, which blocks the hERG channel, may cause cardiotoxicity and oral rat acute toxicity. However, none of the six phytocompounds in this study exhibit the potential to be hERG channel blockers, suggesting that they may not cause hERG channel-related cardiotoxicity [319,323].

#### 4.3.4. Biosynthesis of Tepernoid Class

**Figure 1:**
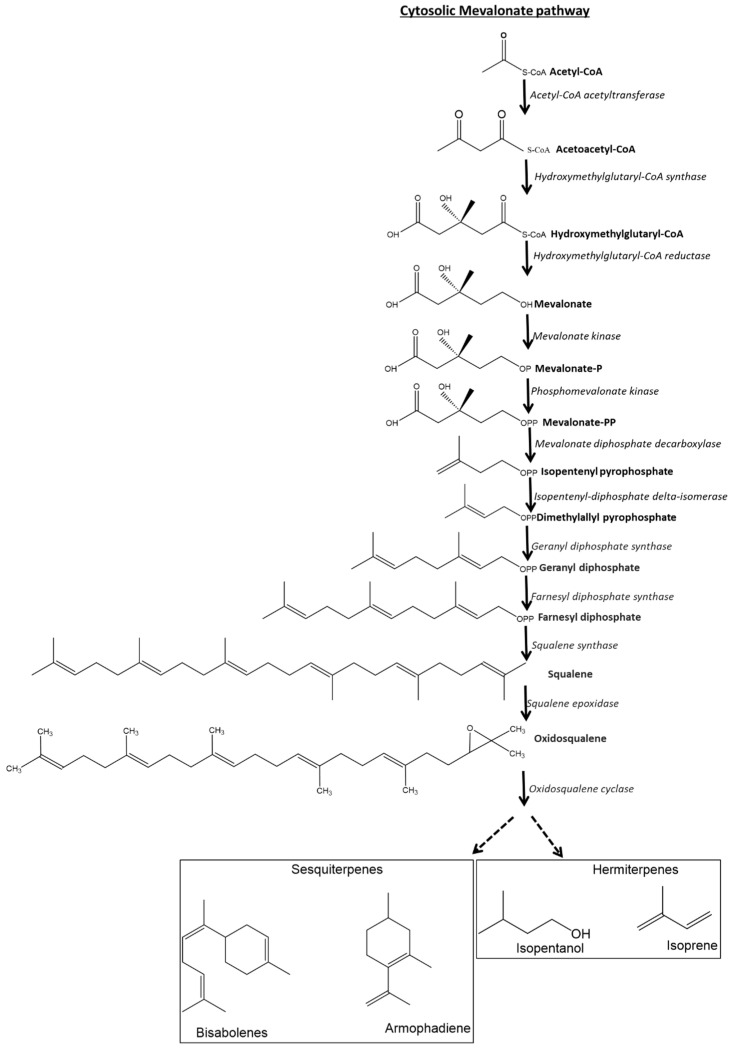
Terpene biosynthesis in the cytosol through the mevalonate pathway. Starting with acetyl-CoA, this process produces terpene precursors (isopentenyl pyrophosphate and dimethylallyl pyrophosphate), serving as building blocks for various terpenes with diverse biological functions.

The *Euphorbia* species contains a diverse array of compounds, including terpenoids, steroids, fatty acids, and phenolic compounds. Each compound has its own specific biosynthetic pathway. However, the current study only focuses on the biosynthesis pathway of terpenoids, as it is the main class of compounds isolated from *Euphorbia* species. A schematic diagram of the biosynthesis is presented in Figure 1.

## 5. Conclusions and Future Perspective

This review focuses on the medicinal properties of 15 species of *Euphorbia*, particularly their potential as alternative treatments for cancer. These plants contain unique compounds such as triterpenoids, tannins, diterpene esters, and sterols. Several studies have reported that the *Euphorbiacea* family contains phytochemicals that lead to the isolation of various classes of triterpenoids. Some of these triterpenoids include euphol (found in *E. trigona*, *E. tirucalli*, *E. enopla*, *E. coerulescens*, *E. ammak*, and *E. cooperi*), cycloartenol, lupeol, amyrins, taraxerols, friedelin, and tirucallol (found in *E. trigona*). Tannin classes include ellagic acid, 1-O-Galloyl-3,6-hexahydroxydiphenyl-β-D-, tri methyl ellagic acid, and unknown structures tirucallin A and tirucallin B. Sterol classes such as β-sitosterol, stigmasterol, and obtusifoliol are also present. Diterpernoid classes such as diterpene esters, 12-Deoxy phorbol, phorbol, ingenol, ingenol triacetate, 16-Hydroxy-12-desoxyphorbol, and 12-Deoxyphorbol-13-isobutyrate-16-angelate-20-acetate are also found. These compounds have shown cytotoxic effects through various mechanisms of action, including cell proliferation, differentiation, and also apoptosis. Out of all the *Euphorbia* species studied, *E. trigona* had the most anticancer isolated compounds, with a total of 14. *E. tirucalli* followed closely behind with 13, *E. cooperi* had 8, and the others had fewer or none at all. Other studies have reported promising results, although others have found no significant inhibitory activity against certain cell lines. The in silico results further revealed that several *Euphorbia*-derived top compounds show good cytotoxic potential against multiple cancer cell lines, indicating anticancer tendency against stomach carcinoma, kidney carcinoma, skin melanoma, liver hepatoblastoma, lung carcinoma, brain glioma, colon adenocarcinoma, thyroid carcinoma, etc. Most of these compounds are drug-like, as indicated by Lipinski screening and Veber parameters, which were derived from good physicochemical properties. Most of the EDCs were predicted to possess good pharmacokinetic tendencies, as indited by the ADMET properties.

Therefore, building upon the diverse compounds identified, such as triterpenoids, tannins, diterpene esters, and sterols, requires further in-depth pharmacological studies to unravel the precise mechanisms of action and potential synergies among these compounds. Subsequent clinical trials would be pivotal in assessing the safety and efficacy of these compounds in human subjects. Additionally, exploring combination therapies with existing cancer treatments, investigating the bioavailability of isolated compounds, and identifying new, yet-to-be-evaluated compounds like euphorbin A, euphorbin B and tirucallin A are crucial areas of focus. Development of optimized formulations, understanding the factors contributing to variability in study results, and fostering collaboration between diverse research fields are imperative for advancing the potential of *Euphorbia* species in cancer therapeutics. Furthermore, public awareness initiatives and the integration of ethnobotanical knowledge can contribute to a holistic understanding of the historical and contemporary significance of these plants in medicinal contexts. This comprehensive and multidisciplinary approach underscores the need for ongoing research to harness the full therapeutic potential of *Euphorbia* species for cancer treatment. Overall, this report aims to provide scientific credibility to the traditional use of *Euphorbia* species for medicinal purposes.

## Figures and Tables

**Table 1:** *Euphorbia* spp of Southern Africa and its neighboring countries, their toxicities, and traditional uses.

Botanical Name	Synonym	Vernacular Name	Location	Growing Condition	Traditional Use	Bioactive Property	Toxicity	Reference
*Euphorbia trigona*	*Euphorbia hermentiana Lem*	African Milk Tree (E)	West Africa, tropical Asia, India	Dry tropical forests; semiarid; direct sunlight	Respiratory infections, urinary tract infections, gonorrhea, tumors, warts, intestinal parasites, etc.	Anticancer	Skin irritation	[16,17,18,19,20,21]
*Euphorbia cooperi*	*E. cooperi var. cooperi, E. cooperi var. calidicola*	Candelabra *Euphorbia* (E), Umhlonhlo (N), Tshikondengala (V), Mokhoto (T), Mohlohlo (S)	South Africa, Mozambique, Zimbabwe, Botswana	Well-drained soil; direct sunlight	Sore stomach, bloatedness, paralysis, wound healing	Breast cancer inhibitor	Skin irritation, blindness, throat burning	[22,23,24,25,26,27,28]
*Euphorbia ammak*	*-*	African Candelabra/Desert Candelabra (E)	Saudi Arabia, Yemen Peninsula	No data reported	No data reported	Anticancer, antileishmanial activity, anti-H1N1 influenza virus	Skin and eye irritation	[29,30,31,32,33]
*Euphorbia tirucalli*	*Euphorbia laro Drake*	Rubber-Hedge *Euphorbia* (E), umSululu (Z), umHlonthlo (X), motsetse/setlharesetola (Ts), mahumbana (T), etc.	Eastern tropical Africa, South Africa, Indian Ocean	Rock garden; well-drained soil; mild to warm climate	Snakebites, sexual impotence, warts, wounds, swollen glands, edema, tumors, etc.	Antibacterial, molluscicidal, antiherpetic, antitumor effect on various cell lines	Skin irritation, blindness, fish poisoning, fatality	[5,6,34,35,36,37,38,39,40,41,42,43,44,45,46,47,48,49,50,51,52]
*Euphorbia clavarioides*	*Euphorbia basutica*	Lion’s Spoor (E), Melkpol (A), iSantilele/isihlekehleke (Z), sehlehle/sehloko (S)	South Africa, Lesotho	Direct sunlight; mineral soil; grassland gardens	Skin rash, acne, sores, burns, cracked heels, swollen feet, herpes, etc.	No data reported	Skin irritation, bird lime	[53,54,55,56,57]
*Euphorbia gorgonis*		Nkalimasane (Z), Melkbol (A)	South Africa (Eastern Cape)	Well-drained soil; direct sunlight	Wounds, swelling, skin problems	Antibacterial, antimicrobial activity, effect on cancer cell lines	Skin irritation	[58,59,60]
*Euphorbia bupleurifolia*	*Tithymalus bupleurifolius*	Pine Cone Plant (E), Melkbol (A), Intsele (X), Insema (Z)	South Africa (Eastern Cape, Transkei, Natal)	Warm, moist conditions; moderate sunlight	Cancerous sores, cracked feet, eczema, rashes, wounds, teeth cleaning, swellings	No data reported	Skin irritation	[61,62,63,64]
*Euphorbia enopla*	*Euphorbia enopla var. enopla*	Milk-Barrel/Pincushion *Euphorbia* (E)	South Africa (Eastern Cape, arid/semiarid Karoo)	Well-drained soil; direct sunlight	No data reported	No data reported	Skin irritation, toxic to Vero cell lines	[65]
*Euphorbia coerulescens*	*Euphorbia virosa*	Sweetnoors (A)	South Africa (Cape Province)	Sunny to half-shady; rocky, gritty–sandy soils	No data reported	No data reported	Skin irritation, blindness, throat irritation	[66]
*Euphorbia polygona*		African Milk Barrel (E)	South Africa (Eastern Cape)	Well-drained soil; high temperature	No data reported	No data reported	Eye irritation, skin irritation, paralysis	[65]
*Euphorbia horrida var.*	*Euphorbia horrida Boiss.*	African Milk Barrel (E)	South Africa (Wittepoort/Karoo)	Well-drained soil; dry, sunny conditions	No data reported	No data reported	Inhibition of Vero cell line	[65]
*Euphorbia arabica*	*Euphorbia neopolycnemoides*	Klein Bont *Euphorbia* (E), Umhlonhlo (Z)	Botswana, Mozambique, Zimbabwe, Limpopo, Mpumalanga, KwaZulu-Natal	Stony grassland	Warts, stomachache, skin infections	Antibacterial	Inhibition of Vero cell line	[67,68]
*Euphorbia ledienii*	*Euphorbia ledienii A. Berger var. ledienii*	Crested *Euphorbia* (E)	South Africa (Western Cape)	Well-drained soil; sunny conditions	No data reported	No data reported	Skin irritation	[69]
*Euphorbia ferox*	*Euphorbia capitosa*	Milkweed (E)	South Africa (Western Cape)	Drained sandy soil; rocky outcrops	No data reported	No data reported	Poisonous latex	
*Euphorbia stellata*	*Euphorbia squarrosa, E. radiata, E. scolopendrea, E. uncinata*	Spurge (E)	South Africa (Eastern Cape)	Well-drained soil; sunny environment	No data reported	No data reported	Skin and eye irritation	

English (E), Sotho (S), Zulu (Z); Xhosa (X); Afrikaans (A), Ndebele (N), Tsonga (T), Tswana (Ts), Venda (V).

**Table 2:** Comprehensive overview of isolated compounds from individual plants.

Plant	Compounds	Reference
*Euphorbia trigona*	Euphol, Cycloartenol, Cycloartanol, Lupeol, α-amyrin, β-amyrin, Betulinic acid, Taraxerol, β-sitosterol, Taraxerol acetate, Friedelin, Friedelan 3 α- and 3 βα-ols, 24-ethylene cycloartanol, Epi-friedelinyl acetate, 3β, Friedelinol, Rhoiptlenone	[70,71]
*Euphorbia ledienii*	Isobutyric, 2-Methylbutyric acid, 12-Deoxyphorbol-13-isobutyrate-20-acetate, 12-Deoxyphorbol-13-(2-methylbutyrate)-20-acetate, Phorbol, 12-Deoxy phorbol, 12-Deoxy-16-hydroxy phorbol, Ingol-7,8,12-acetate, ditiglate	[72,73,74]
*Euphorbia tirucalli*	Triterpenes, Euphol, Diterpene esters, Euphorbiane, 12-Deoxyphorbol esters, Ingenol, β-Sitosterol, Euphorbol, Hexacosonate, 12-Deoxy-4β-hydroxyphorbol-13-phenylacetate-20-acetate, 12, 20-Dideoxyphorbol-13-isobutyrate, Glut-5-en-3-β-ol, Tirucalicine, Tri-methyl ellagic acid, Terpenic alcohol, Isoeuphorol, Taraxasterol, Tirucallol, Ketone euphorone, Resin, Ellagic acid, Taraxerol, 3,3′-Di-O-methylellagic acid, Euphorbin A, Euphorbin B, Tirucallin A, Tirucallin B, Euphorbol, Cycloartenol, 24-Methylenecycloartenol, Ingenol triacetate, β-amyrin, Rhoiptlenone, 3β-friedelinol, Epi-friedelinyl acetate, 24-ethylene cycloartanol, Friedelan 3 α- and 3 βα-ols, Friedelin, Taraxerol acetate, Betulinic acid, β-amyrin, α-amyrin, Lupeol, Cycloartanol, *β*-amyrone, Glutinone, Taraxerone, Glut-5-en-3-*β*-ol and cycloart-23-ene-3-*β*, 25-diol, Euphorcinol, Euphorginol, 12,13,20-tri-*O*-acetylphorbol, 3,5,20-tri-*O*-acetlingenol 20-acetate	[8,9,21,35,36,75,76,77,78,79,80,81,82,83,84,85,86,87,88,89,90,91,92]
*Euphorbia enopla*	Euphol, Tirucallol	[93]
*Euphorbia coerulescens*	Angelate acetate isobutyrate, Fatty acids, Acetate laurate, α-Methyl butyrate, Heptanoate, Laurate, Euphol, Tirucallol, Euphorbol	[66,89]
*Euphorbia ammak*	α-glutinol, Stigmasterol, Euphol, Euphorbol	[93,94]
*Euphorbia cooperi*	16-Hydroxy-12-desoxyphorbol, Euphol, Obtusifoliol, 12-Deoxyphorbol-13-isobutyrate-16-angelate-20-acetate, Euphorbilactone, Norsesquiterpenoid, Arachiside A, Glutinol, 16-Angeloyloxy-13α-isobutanoyloxy-4β,9α,20-trihydroxytiglia-1,5-diene-3,7-dione, 20-Acetoxy-16-angeloyloxy13α-isobutanoyloxy-4β,9α,20-tetrahydroxytiglia-1,5-diene-3-one, Gallic acid, Bervifolin, Carboxylic acid, Kampferol-3-O-ß-D-rutinoside, 1-O-Galloyl-3,6-hexahydroxydiphenyl-β-D glucopyranoside, 3,3′ Dimethoxy ellagic acid 3,4,4′ Trimethoxyellagic acid, Ellagic acid, Kaempferol, 7-galloyl catechin, kaempferol 3-*O*-*β*-(6″-*O*-galloyl)-glucopyranoside, triesters-16-hydroxy-12-desoxy-phorbol	[9,24,95,96,97]
*Euphorbia horrida*	17-Hydroxyingenol-17-benzoate-20-angelate, Diterpene esters	[98]

**Table 3:** Phytochemicals extracted from the selected *Euphorbia* spp.

Plant	Phytochemical/s	Reference
*Euphorbia trigona*	Saponins, alkaloids, flavonoids, glycosides, sterols and triterpernoids, tannins	[17,71,99]
*Euphorbia cooperi*	Triterpernoid	[95]
*Euphorbia ammak*	Alkaloids, saponins, glycosides	[31]
*Euphorbia tirucalli*	Triterpernoid, phenols, flavonoids, tannins, alkaloids, saponins, glycosides, steroids	[37,100]
*Euphorbia clavarioides*	Alkaloids, flavonoids, saponins, tannins, terpenoids, phytosterols, glycosides, triterpenoids, anthraquinone	[65,101]
*Euphorbia gorgonis*	Phytosterols, glycosides, triterpenoids, flavonoids, alkaloids, saponins	[60,65]
*Euphorbia bupleurifolia*	Phytosterols, tannins, glycosides, triterpenoids, saponins, flavonoids and alkaloids	[62,65]
*Euphorbia enopla*	Phytosterols, glycosides, triterpenoids, flavonoids, alkaloids, tannins, anthraquinone	[65]
*Euphorbia polygona*	Phytosterols, tannins, glycoside, triterpenoids, flavonoids, alkaloids	[65]
*Euphorbia horrida Var*	Phytosterols, pentose, tannins, glycosides, triterpenoids, anthraquinones, saponins, flavonoids	[65]
*Euphorbia arabica*	Phytosterols, tannins, glycosides, triterpenoids, anthraquinones, flavonoids	[65]
*Euphorbia ledienii*	No data reported	
*Euphorbia ferox*	No data reported	
*Euphorbia stellata*	No data reported	
*Euphorbia coerulescens*	No data reported	

**Table 4:** Classification of crude and isolated compounds from 15 *Euphorbia* species.

Class	Subclass	Compounds
Terpenoids	Triterpenoids	Euphol, Cycloartanol, Lupeol, α-amyrin, β-amyrin, Betulinic acid, Taraxerol, Taraxerol acetate, Friedelin, Friedelan-3-β-ol, 3β-Friedelinol
	Phytosterols	Cycloartenol, β-sitosterol, 24-Methylenecycloartanol, Tirucallol, Obtusifoliol, Glutinol, Triterpene euphol, 24-Methylene cycloartenol, Ingenol triacetate, Terpenic alcohol, Taraxasterol, Stigmasterol
	Other Terpenoids	12-Deoxyphorbol-13-isobutyrate-20-acetate, Phorbol, 12-Deoxyphorbol ester, 12-Deoxy-16-hydroxy-phorbol, 16-Hydroxy-12-desoxyphorbol, 12-Deoxyphorbol-13-isobutyrate-16-angelate-20-acetate, Ingenol, Glut-5-en-3-β-ol
Phenolics	Phenolic Acids	Gallic acid
	Tannins	Bervifolin carboxylic acid, 3, 3′-Dimethoxy ellagic acid, Ellagic acid, 1-O-Galloyl-3,6-hexahydroxydiphenyl-β-D-glucopyranoside/corilagin, Tri-methyl ellagic acid
	Flavonoids	Kaempferol, Kampferol-3-O-ß-D-rutinoside
	Phenols	3′-Di-O-methylellagic acid
Fatty Acids	-	Laurate
Miscellaneous	Diterpene esters	Diterpene esters
Polyphenolic Compounds	Flavonoids	Flavonoids
Organic Compounds	Alkaloids	Alkaloids
Glycosides	-	Saponins
Polyphenols	Tannins	Tannins
Acetal Derivatives	Glycosides	Glycosides
Phenolics	Anthraquinones	Anthraquinones

**Table 5:** Bioactive compounds isolated from 15 *Euphorbia* species alongside their biological activities and subclasses.

Compounds	Subclasses	Pharmacological/Biological Activity	Reference
Euphol	Euphane	Anticancer, cytotoxicity, anti-nociceptive, anti-inflammatory, HIV-1 reverse transcriptase inhibitor	[76,102,103]
Cycloartenol	Cycloartane	Anti-inflammatory, antitumor, antioxidant, antibiosis, anti-Alzheimer’s disease, apoptotic, analgesic, bactericidal	[106,107,108,109]
Cycloartanol	Cycloartane	Antifungal, vasodepressor, antitumor	[104,105,110]
Lupeol	Lupane	Anticancer, anti-inflammatory, antimicrobial, antiprotozoal, antiproliferative, antiangiogenic, anti-invasive, cholesterol lowering	[40,111,112,113,114,115,116,117]
α-Amyrin	Oleanane	Cytotoxicity, antifungal, anti-inflammatory	[120,122,123,124]
β-Amyrin	Oleanane	Anti-inflammatory, nitric oxide inhibitor, reactive oxygen species activator, anticancer	[118,119,121,122,125]
Betulinic Acid	Pentacyclic Triterpenes	Antitumor, antidiabetic, anti-inflammatory, HIV-1 reverse transcriptase inhibitor, antiviral, hepatoprotective activity	[126,127,128,129,130,131,132,133,134,135]
Taraxerol	Taraxarane	Anticancer, anti-inflammatory, apoptotic, antioxidative, antimicrobial, antifungal, antidiabetic	[138,139,140,141]
β-Sitosterol	Sterols	Anti-inflammatory, anticancer, antiproliferative, analgesic, antimicrobial	[125,142,143,144,145]
Taraxerol Acetate	Taraxarane	Anti-inflammatory, cyclooxygenase inhibitor	[136,137]
Friedelin	Friedelane	Cytotoxicity, antibacterial	[153,154]
Friedelan-3α- and -3β-Ols	Friedelane	Anticancer	[154]
24-Ethylene Cycloartanol	Cycloartane	Anti-inflammatory, antifungal	[125,155]
Epi-Friedelinyl Acetate	Friedelane	No data reported	[156]
3β-Friedelinol	Friedelane	Antibacterial, cytotoxicity	[156,157,158,159]
Rhoiptlenone	No data reported	No data reported	[156]
Isobutyric Acid	Carboxylic Acid	Irritant	[73,160]
2-Methylbutyric Acid	No data reported	No data reported	[73]
12,20-Dideoxyphorbol-13-Isobutyrate	Phorbol	No data reported	[148]
12-Deoxyphorbol-13-Isobutyrate-20-Acetate	Phorbol	Antifungal	[161]
12-Deoxyphorbol-13-(2-Methylbutyrate)-20-Acetate	Phorbol	No data reported	[72]
Phorbol	Phorbol	Tumor promoter, apoptosis	[7,162]
12-Deoxyphorbol	Phorbol	Antitumor, apoptotic	[163,164,165,166]
12-Deoxy-16-Hydroxyphorbol	Phorbol	Irritant, tumor promoter	[167]
Ingol-7,8,12-Acetate, Ditiglate	Ingenane	No data reported	[160]
Triterpenes Euphol	Glycosides	Anticancer, anti-inflammatory	[52,88,168]
Diterpene Esters	Terpenoids	Anticancer, cytotoxicity, tumor promoter, irritant, pro-inflammatory	[7,80,169,170,171]
12-Deoxyphorbol Esters	Phorbol	Irritant, pro-inflammatory, tumor promoter	[172,173,174,175]
Ingenol	Ingenane	Cytotoxicity, HIV reverse transcriptase inhibitor	[102,152]
Euphorbol Hexacosonate	No data reported	No data reported	[148]
12-Deoxy-4β-Hydroxyphorbol-13-Phenyl Acetate-20-Acetate	No data reported	No data reported	[148]
12,20-Dideoxyphorbol-13-isobutyrate	Phorbol	No data reported	[148]
Glut-5-en-3-β-ol	Pentacyclic Triterpene	Antibacterial	[176]
Tirucalicine	Tirucallane	No data reported	[81,148,177]
Tri-methyl Ellagic Acid	Ellagic Acid Derivatives	Anticancer	[148,150,151]
Terpenic Alcohol	Steroidal Triterpenoids	Antibacterial, irritant	[148,149]
Isoeuphorol	Ingenane-type Triterpenoids	No data reported	[148]
Taraxasterol	Sterol	Tumor inhibition, antiproliferation	[178]
Tirucallol	Tirucallane	Anti-inflammatory, HIV inhibition reverse transcriptase	[146,147]
Ketone Euphorone	Euphane-type Triterpenoids	No data reported	[179]
Resin	No data reported	Digestive enzyme, antioxidant, antispasmodic, hypotensive, hepatoprotective, antiviral, antifungal, anticancer, anxiolytics, anthelmintic	[180]
Ellagic Acid	Ellagic Acid Derivatives	Hepatoprotective activity, antiproliferative activity, antioxidant	[181]
3,3′-Di-O-Methylellagic Acid	Ellagic Acid Derivatives	Antioxidant, moderate antibacterial activity, antimicrobial activity, anticancer	[182,183,184,185]
Euphorbin A	No data reported	No data reported	[79]
Euphorbin B	No data reported	No data reported	[79]
24-Methylenecycloartenol	Cycloartane	Antioxidant, anti-inflammatory	[125,186]
Tirucallin A	Tirucallane	No data reported	[79]
Tirucallin B	Tirucallane	No data reported	[79]
Euphorbol	Euphane-type Triterpenoids	Antibacterial, anti-inflammatory	[94,187]
Fatty Acids	No data reported	No data reported	[66]
Ingenol Triacetate	Ingenane-type Triterpenoids	Antimicrobial, antitumor	[188,189]
Angelate Acetate Isobutyrate	Ingenane-type Triterpenoids	No data reported	[66]
Acetate Laurate	No data reported	No data reported	[66]
α-Methyl Butyrate	No data reported	No data reported	[66]
Heptanoate	No data reported	No data reported	[66]
Laurate	Medium-chain Fatty Acids	Antibacterial	[66,190]
α-Glutinol	Pentacyclic triterpenes	Anti-proliferation, cytotoxicity	[94,191,192]
Stigmasterol	Phytosterol	Cytotoxicity, antioxidant, hypoglycemic, thyroid inhibitor	[94,193]
16-Hydroxy-12-Deoxyphorbol	Phorbol	Antitumor, tumor promoter	[97,194]
12-Deoxyphorbol-13-Isobutyrate-16-Angelate-20-Acetate	Phorbol-type Diterpenoid	Cytotoxicity	[24,28,95]
Obtusifoliol	Lanostane	Cytotoxicity	[24,28,95,195]
Euphorbilactone	Ingenane-type Triterpenoids	No data reported	[95]
Norsesquiterpenoid	No data reported	No data reported	[95]
Arachiside A	Ingenane-type Triterpenoids	No data reported	[95]
Glutinol	Pentacyclic Triterpenes	Antiproliferation	[191]
16-Angeloyloxy-13α-Isobutanoyloxy-4β,9α,20-Trihydroxytiglia-1,5-Diene-3,7-Dione	No data reported	No data reported	[95]
20-Acetoxy-16-Angeloyloxy-13α-Isobutanoyloxy-4β,9α,20-Tetrahydroxytiglia-1,5-Diene-3-One	No data reported	No data reported	[95]
Gallic Acid	Gallic Acid Derivatives	Hepatoprotective activity, antioxidant	[181]
Bervifolin Carboxylic Acid	Gallic Acid Derivatives	Hepatoprotective activity, antioxidant	[181]
Kampferol-3-O-β-D-Rutinoside	Flavonoids	Hepatoprotective activity, antioxidant	[181]
1-O-Galloyl-3,6-Hexahydroxydiphenyl-β-D-Glucopyranoside	Gallic Acid Derivatives	Antitumor, anti-inflammatory, antioxidant, hepatoprotective, antimicrobial, antihypertensive, antidiabetic, anti-HIV, antifungal	[28,196,197,198,199,200,201,202]
3,3′ Dimethoxy ellagic acid	Ellagic Acid Derivatives	Hepatoprotective activity, antioxidant	[181]
3,4,4′-Trimethoxyellagic Acid	Ellagic Acid Derivatives	Hepatoprotective activity, antioxidant	[181]
Kampferol	Flavonoids	Hepatoprotective activity, antiproliferative activity, antioxidant	[28]
17-Hydroxyingenol-17-Benzoate-20-Angelate	Flavonoids	No data reported	[98]

## Data Availability

The data are contained within this article.

## Supplementary Materials

The following supporting information can be downloaded at https://www.mdpi.com/article/10.3390/plants14030469/s1: Figure S1: Illustrations of various medicinal plants belonging to the *Euphorbia* genus. S1A: *Euphorbia* trigona tree and pot plant (source: https://tropical.theferns.info/image.php?id=Euphorbia+trigona; https://laidbackgardener.blog/2018/12/09/when-a-red-euphorbia-turns-green/ (accessed 6 February 2025)); S1B: *Euphorbia* ledienii plant with stem and flowers; S1C: *Euphorbia* horrida plant with stem and flowers; S1D: *Euphorbia* enopla plant with stem and flowers; S1E: *Euphorbia* coerulescences plant with stem and flowers (source: https://serreslavoie.com/en/products/euphorbia-coerulescens; https://www.agaveville.org/viewtopic.php?t=2025 (accessed 6 February 2025)). S1F: *Euphorbia cooperi* tree and fruits (source: https://pza.sanbi.org/euphorbia-cooperi (accessed 6 February 2025)); S1G: *Euphorbia tirucalli* plant and flower (source: https://za.pinterest.com/pin/298785756510953587/; https://za.pinterest.com/pin/474426141998696380/ (accessed 6 February 2025)). S1H *Euphorbia ammak* tree and flower (source: https://luirig.altervista.org/cpm/albums/bot-units07/euphorbia-ammak16454.jpg (accessed 6 February 2025)); S1I: *Euphorbia clavarioides* plant with flowers and stem (source: https://www.inaturalist.org/observations/38217609; https://www.llifle.com/Encyclopedia/SUCCULENTS/Family/Euphorbiaceae/32951/Euphorbia_clavarioides_var._truncata (accessed 6 February 2025)). S1J: *Euphorbia gorgonis* plant with flowers and stem (source: https://www.bihrmann.com/caudiciforms/SUBS/eup-gor-sub.asp (accessed 6 February 2025)). S1K: *Euphorbia bupleurifolia* plant with stem and flowers (source: https://www.cactus-art.biz/schede/EUPHORBIA/Euphorbia_bupleurifolia/Euphorbia_bupleurifolia/Euphorbia_bupleurifolia.htm; https://en.wikipedia.org/wiki/Euphorbia_bupleurifolia  (accessed 6 February 2025)). S1L: *Euphorbia polygona* plant with stem and flowers (source: https://www.biodiversityexplorer.info/plants/euphorbiaceae/euphorbia_polygona.htm (accessed 6 February 2025)). S1M: *Euphorbia Arabica* plant with stem and flowers (source: https://www.zimbabweflora.co.zw/speciesdata/species.php?species_id=136210; https://www.biodiversityexplorer.info//plants/euphorbiaceae/images/136210-2_658w.jpg (accessed 6 February 2025); S1N: *Euphorbia ferox* plant with stem and flowers (source https://planetdesert.com/products/euphorbia-ferox-cactus-cacti-real-succulent-plant; https://www.cactofili.org/specie.asp?gen=euphorbia&sp=ferox (accessed 6 February 2025)). S1O: *Euphorbia stellate* plant with stem and flowers (source: https://worldofsucculents.com/euphorbia-stellata/#google_vignette (accessed 4 April 2024); https://www.cactofili.org/specie.asp?gen=euphorbia&sp=stellata (7 February 2025). Table S1. Compounds with high cytotoxic probability against 3 or more cancer cell lines. Table S2. Physicochemical parameters and solubility tendency.

## References

[1] Bijekar S.R., Gayatri M.C. (2014). Ethanomedicinal Properties of Euphorbiaceae Family—A Comprehensive Review. Int. J. Phytomedicine.

[2] Aleksandrov M., Maksimova V., Koleva Gudeva L. (2019). Review of the anticancer and cytotoxic activity of some species from genus Euphorbia. Agric. Conspec. Sci..

[3] Adedapo A.A., Saba A.B., Dina O.A., Oladejo G.M.A. (2004). Effects of dexamethasone on the infectivity of *Trypanosoma vivax* Y486 and the haematological changes in Nigerian domestic chickens (*Gallus gallus domesticus*). Vet. Arhiv.

[4] Villanueva J., Quirós L.M., Castañón S. (2015). Purification and Partial Characterization of a Ribosome-Inactivating Protein from the Latex of *Euphorbia trigona* Miller with Cytotoxic Activity toward Human Cancer Cell Lines. Phytomedicine.

[5] Betancur-Galvis L.A., Morales G.E., Forero J.E., Roldan J. (2002). Cytotoxic and antiviral activities of Colombian medicinal plant extracts of the Euphorbia genus. Mem. Inst. Oswaldo Cruz.

[6] Mwine T.J., Damme V.P. (2011). Why Do Euphorbiaceae Tick as Medicinal Plants? A Review of Euphorbiaceae Family and Its Medicinal Features. J. Med. Plants Res..

[7] Fürstenberger G., Hecker E. (1985). On the Active Principles of the Spurge Family (Euphorbiaceae) XI. The Skin Irritant and Tumor Promoting Diterpene Esters of *Euphorbia tirucalli* L. Originating from South Africa. Z. Naturforsch. C.

[8] Cataluna P., Rates S.M.K. (1997). The traditional use of the latex from *Euphorbia tirucalli* Linnaeus (Euphorbiaceae) in the treatment of cancer in South Brazil. II WOCMAP Congress Medicinal and Aromatic Plants, Part 2: Pharmacognosy, Pharmacology, Phytomedicine, Toxicology.

[9] Mavundza E.J., Street R., Baijnath H. (2022). A Review of the Ethnomedicinal, Pharmacology, Cytotoxicity, and Phytochemistry of the Genus Euphorbia in Southern Africa. S. Afr. J. Bot..

[10] Lu L., Zhang J., Xie Y., Gao F., Xu S., Wu X., Ye Z. (2020). Wearable health devices in health care: Narrative systematic review. J. Med. Internet Res..

[11] Lagunin A.A., Dubovskaja V.I., Rudik A.V., Pogodin P.V., Druzhilovskiy D.S., Gloriozova T.A., Poroikov V.V. (2018). CLC-Pred: A Freely Available Web-Service for In Silico Prediction of Human Cell Line Cytotoxicity for Drug-Like Compounds. PLoS ONE.

[12] Daina A., Michielin O., Zoete V. (2017). SwissADME: A Free Web Tool to Evaluate Pharmacokinetics, Drug-Likeness, and Medicinal Chemistry Friendliness of Small Molecules. Sci. Rep..

[13] Pires D.E.V., Blundell T.L., Ascher D.B. (2015). pkCSM: Predicting Small-Molecule Pharmacokinetic and Toxicity Properties Using Graph-Based Signatures. J. Med. Chem..

[14] Adetunji J.A., Ogunyemi O.M., Gyebi G.A., Adewumi A.E., Olaiya C.O. (2023). Atomistic Simulations Suggest Dietary Flavonoids from Beta Vulgaris (Beet) as Promising Inhibitors of Human Angiotensin-Converting Enzyme and 2-Alpha-Adrenergic Receptors in Hypertension. Bioinform. Adv..

[15] Ogunyemi O.M., Gyebi G.A., Ibrahim I.M., Esan A.M., Olaiya C.O., Soliman M.M., Batiha G.E.-S. (2022). Identification of Promising Multi-Targeting Inhibitors of Obesity from Vernonia Amygdalina through Computational Analysis. Mol. Divers..

[16] Tada M., Seki H. (1989). Toxic Diterpenes from *Euphorbia trigona* (Saiunkaku: An Indoor Foliage Plant in Japan). Agric. Biol. Chem..

[17] Nashikkar N., Begde D., Bundale S., Mashitha P., Rudra J., Upadhyay A. (2012). Evaluation of the Immunomodulatory Properties of *Euphorbia trigona*—An In Vitro Study. Int. J. Inst. Pharm. Life Sci..

[18] Marathe K., Nashikkar N., Bundale S., Upadhyay A. (2019). Analysis of Quorum Quenching Potential of *Euphorbia trigona* Mill. Int. J. Pharm. Sci. Res..

[19] Siddique N.A., da Silva J.A.T., Bari M.A. (2010). Preservation of Indigenous Knowledge Regarding Important and Endangered Medicinal Plants in Rajshahi District of Bangladesh. J. Plant Sci..

[20] Bouquet A.J. (1969). Natural Products as an Alternative Remedy.

[21] Lynn K.R., Clevette-Radford N.A. (1985). Four serine proteases from the latex of *Euphorbia tirucalli*. Can. J. Biochem. Cell Biol..

[22] Luseba D., Van der Merwe D. (2006). Ethnoveterinary medicine practices among Tsonga speaking people of South Africa. Onderstepoort J. Vet. Res..

[23] Gildenhuys S. (2006). The three most abundant tree Euphorbia species of the Transvaal (South Africa). Euphorbia World.

[24] El-Sherei M.M., Islam W.T., El-Dine R.S., El-Toumy S.A., Ahmed S.R. (2015). Phytochemical investigation of the cytotoxic latex of *Euphorbia cooperi* NE Br. Aust. J. Basic Appl. Sci..

[25] Hedberg I., Staugård F. (1989). Traditional Medicinal Plants.

[26] Gundidza M., Sorg B., Hecker E. (1992). A skin irritant phorbol ester from *Euphorbia cooperi* NE Br. Cent. Afr. J. Med..

[27] Engi H., Vasas A., Redei D., Molnár J., Hohmann J. (2007). New MDR Modulators and Apoptosis Inducers from Euphorbia Species. Anticancer. Res..

[28] Morsi S.R.A. (2015). Phytochemical and Biological Study of Certain Euphorbia Species Cultivated in Egypt. Ph.D. Dissertation.

[29] Carter S. (1994). A preliminary classification of Euphorbia subgenus Euphorbia. Ann. Mo. Bot. Gard..

[30] Almehdar H., Abdallah H.M., Osman A.M.M., Abdel-Sattar E.A. (2012). In vitro cytotoxic screening of selected Saudi medicinal plants. J. Nat. Med..

[31] Al-Hajj M.M.A., Al-Shamahy H.A., Alkhatib B.Y., Moharram B.A. (2018). In vitro anti-leishmanial activity against cutaneous Leishmania parasites and preliminary phytochemical analysis of four Yemeni medicinal plants. Univ. J. Pharm. Res..

[32] Kiyohara H., Ichino C., Kawamura Y., Nagai T., Sato N., Yamada H., Salama M.M., Abdel-Sattar E. (2012). In vitro anti-influenza virus activity of a cardiotonic glycoside from *Adenium obesum* (Forssk.). Phytomedicine.

[33] Abdel-Sattar E., Maes L., Salama M.M. (2010). In Vitro Activities of Plant Extracts from Saudi Arabia Against Malaria, Leishmaniasis, Sleeping Sickness, and Chagas Disease. Phytother. Res..

[34] Watt J.M., Breyer-Brandwijk M.G. (1962). The Medicinal and Poisonous Plants of Southern and Eastern Africa Being an Account of Their Medicinal and Other Uses, Chemical Composition, Pharmacological Effects and Toxicology in Man and Animal.

[35] Baslas R.K., Gupta N.C. (1982). Chemical constituents of the bark of *Euphorbia tirucalli*. Indian J. Pharm. Sci..

[36] Kajikawa M., Yamato K.T., Fukuzawa H., Sakai Y., Uchida H., Ohyama K. (2005). Cloning and characterization of a cDNA encoding β-amyrin synthase from petroleum plant *Euphorbia tirucalli* L. Phytochemistry.

[37] Shivkumar S.P. (2015). Ethnopharmacological Validation of Medicinal Plants Treating Skin Diseases in Hyderabad Karnataka Region. http://hdl.handle.net/10603/37479.

[38] Hargreaves B.J. (1991). The spurges of Botswana. Botswana Notes Rec..

[39] Van Damme P. (1989). Studie van *Euphorbia tirucalli* L.: Morfologie, Fysiologie, Teeltvoorwaarden. Ph.D. Dissertation.

[40] Gupta N., Vishnoi G., Wal A., Wal P. (2013). Medicinal value of *Euphorbia tirucalli*. Syst. Rev. Pharm..

[41] Voigt W.E. *Euphorbia tirucalli* L. (Euphorbiaceae). http://pza.sanbi.org/Euphorbia-tirucalli.

[42] MacNeil A., Sumba O.P., Lutzke M.L., Moormann A., Rochford R. (2003). Activation of the Epstein–Barr virus lytic cycle by the latex of the plant *Euphorbia tirucalli*. Br. J. Cancer.

[43] Van den Bosch C., Griffin B.E., Kazembe P., Dziweni C., Kadzamira L. (1993). Are Plant Factors a Missing Link in the Evolution of Endemic Burkitt’s Lymphoma?. Br. J. Cancer.

[44] Kgosiemang I.K.R., Adegoke A.M., Mashele S.S., Sekhoacha M.P. (2023). Green Synthesis of Iron Oxide and Iron Dioxide Nanoparticles Using *Euphorbia tirucalli*: Characterization and Antiproliferative Evaluation against Three Breast Cancer Cell Lines. J. Exp. Nanoscience.

[45] Lirio L.G., Hermano M.L., Fontanilla M.Q. (1998). Antibacterial activity of medicinal plants from the Philippines. Pharm. Biol..

[46] Tiwari S., Singh P., Singh A. (2003). Toxicity of *Euphorbia tirucalli* Plant against Freshwater Target and Non-Target Organisms. Pak. J. Biol. Sci..

[47] Rezende E.L., Bozinovic F., Garland T. (2004). Climatic Adaptation and the Evolution of Basal and Maximum Rates of Metabolism in Rodents. Evolution.

[48] Parekh J., Chanda S. (2007). In Vitro Antimicrobial Activity and Phytochemical Analysis of Some Indian Medicinal Plants. Turk. J. Biol..

[49] Altamimi M., Jaradat N., Alham S., Al-Masri M., Bsharat A., Alsaleh R., Sabobeh R. (2019). Antioxidant, anti-enzymatic, antimicrobial and cytotoxic properties of *Euphorbia tirucalli* L. bioRxiv.

[50] Valadares M.C., Carrucha S.G., Accorsi W., Queiroz M.L. (2006). *Euphorbia tirucalli* L. Modulates Myelopoiesis and Enhances the Resistance of Tumor-Bearing Mice. Int. Immunopharmacol..

[51] Reis R.M., Silva V.A.O., Rosa M.N., Tansini A., Lima J.P.D.S.N., Jones C., Pianowski L.F. (2013). Cytotoxic Effect of Euphol from *Euphorbia tirucalli* on a Large Panel of Human Cancer Cell Lines. J. Clin. Oncol..

[52] De Souza L.S., Puziol L.C., Tosta C.L., Bittencourt M.L., Santa Ardisson J., Kitagawa R.R., Filgueiras P.R., Kuster R.M. (2019). Analytical methods to access the chemical composition of an *Euphorbia tirucalli* anticancer latex from traditional Brazilian medicine. J. Ethnopharmacol..

[53] Moteetee A., Moffett R.O., Seleteng-Kose L. (2019). A Review of the Ethnobotany of the Basotho of Lesotho and the Free State Province of South Africa (South Sotho). S. Afr. J. Bot..

[54] Shale T.L., Stirk W.A., van Staden J. (1999). Screening of Medicinal Plants Used in Lesotho for Antibacterial and Anti-inflammatory Activity. J. Ethnopharmacol..

[55] Maliehe E.B. (1997). Medicinal plants and herbs of Lesotho. Mafeteng Development Project.

[56] Kose L.S., Moteetee A., Van Vuuren S. (2015). Ethnobotanical survey of medicinal plants used in the Maseru district of Lesotho. J. Ethnopharmacol..

[57] Cock I.E., Ndlovu N., Van Vuuren S.F. (2021). The use of South African botanical species for the control of blood sugar. J. Ethnopharmacol..

[58] Johnson T. (2019). CRC Ethnobotany Desk Reference.

[59] Komoreng L., Thekisoe O., Lehasa S., Tiwani T., Mzizi N., Mokoena N., Khambule N., Ndebele S., Mdletshe N. (2017). An ethnobotanical survey of traditional medicinal plants used against lymphatic filariasis in South Africa. S. Afr. J. Bot..

[60] Tiwani T. (2017). Phytochemical Screening, Cytotoxicity, Antimicrobial and Anthelmintic Activity of Medical Plants Used in the Treatment of Lymphatic Filariasis in the Eastern Cape, South Africa. Ph.D. Dissertation.

[61] Afolayan A.J., Grierson D.S., Mbeng W.O. (2014). Ethnobotanical survey of medicinal plants used in the management of skin disorders among the Xhosa communities of the Amathole District, Eastern Cape, South Africa. J. Ethnopharmacol..

[62] Van Wyk B.E., Oudtshoorn B.V., Gericke N. (1997). Medicinal Plants of South Africa.

[63] Iwalewa E.O., McGaw L.J., Naidoo V., Eloff J.N. (2007). Inflammation: The Foundation of Diseases and Disorders. A Review of Phytomedicines of South African Origin Used to Treat Pain and Inflammatory Conditions. Afr. J. Biotechnol..

[64] Assefa A., Bahiru A. (2018). Ethnoveterinary botanical survey of medicinal plants in Abergelle, Sekota and Lalibela districts of Amhara region, Northern Ethiopia. J. Ethnopharmacol..

[65] Mampa S.T.M., Mashele S.S., Sekhoacha M.P. (2020). Applications of Chromatographic Techniques for Fingerprinting of Toxic and Non-toxic Euphorbia Species. Pak. J. Biol. Sci..

[66] Evans F.J. (1978). The irritant toxins of Blue Euphorbia (*Euphorbia coerulescens* Haw.). Toxicon.

[67] Al-Harbi N.A. (2017). Diversity of medicinal plants used in the treatment of skin diseases in Tabuk region, Saudi Arabia. J. Med. Plants Res..

[68] El-Shanwani M.A.A. Al-Nibatat al-Mustakhdima fi al-Tibb al-Sha’abi al-Saudi [Plants Used in Saudi Folk Medicine]. General Directorate of Research Grants Program, KACST, Riyadh 1996. https://scholar.google.com/scholar_lookup?title=Plants+Used+in+Saudi+Folk+Medicine&author=Al-Shanwani,+M.&.

[69] Abdel-Fattah M.R. (1987). The Chemical Constituents and Economic Plants of the Euphorbiaceae. Bot. J. Linn. Soc..

[70] Anjaneyulu V., Rao G.S., Connolly J.D. (1985). Occurrence of 24-epimers of cycloart-25-ene-3β, 24-diols in the stems of *Euphorbia trigona*. Phytochemistry.

[71] Nielsen P.E., Nishimura H., Otvos J.W., Calvin M. (1977). Plant Crops as a Source of Fuel and Hydrocarbon-Like Materials. Science.

[72] Popplewell W.L., Marais E.A., Brand L., Harvey B.H., Davies-Coleman M.T. (2010). Euphorbias of South Africa: Two New Phorbol Esters from *Euphorbia bothae*. S. Afr. J. Chem..

[73] Rédei D., Forgo P., Hohmann J. (2010). New Tigliane Diterpenes from *Euphorbia grandicornis*. Planta Med..

[74] Evans F.J., Kinghorn A.D. (1977). A comparative phytochemical study of the diterpenes of some species of the genera Euphorbia and Elaeophorbia (Euphorbiaceae). Bot. J. Linn. Soc..

[75] Martins C.G., Appel M.H., Coutinho D.S., Soares I.P., Fischer S., de Oliveira B.C., de Souza L.M. (2020). Consumption of Latex from *Euphorbia tirucalli* L. Promotes a Reduction of Tumor Growth and Cachexia, and Immunomodulation in Walker 256 Tumor-Bearing Rats. J. Ethnopharmacol..

[76] Lin M.W., Lin A.S., Wu D.C., Wang S.S., Chang F.R., Wu Y.C., Huang Y.B. (2012). Euphol from *Euphorbia tirucalli* Selectively Inhibits Human Gastric Cancer Cell Growth through the Induction of ERK1/2-Mediated Apoptosis. Food Chem. Toxicol..

[77] Yu H.-C., Shen C., Yi H.-M., Chen T.-H., Hsueh M.-L., Lin C., Don M. (2012). Euphorbiane: A Novel Triterpenoid with an Unprecedented Skeleton from *Euphorbia tirucalli*. J. Chin. Chem. Soc..

[78] Dutra R.C., da Silva K.A.B.S., Bento A.F., Marcon R., Paszcuk A.F., Meotti F.C., Pianowski L.F., Calixto J.B. (2012). Euphol, a tetracyclic triterpene produces antinociceptive effects in inflammatory and neuropathic pain: The involvement of cannabinoid system. Neuropharmacology.

[79] Yoshida T., Yokoyama K.I., Namba O., Okuda T. (1991). Tannins and Related Polyphenols of Euphorbiaceous Plants. VII. Tirucallins A, B and Euphorbin F, Monomeric and Dimeric Ellagitannins from *Euphorbia tirucalli* L. Chem. Pharm. Bull..

[80] Khan A.Q., Malik A. (1990). A new macrocyclic diterpene ester from the latex of *Euphorbia tirucalli*. J. Nat. Prod..

[81] Khan A.Q., Ahmed Z., Kazmi N.U.H., Malik A., Afza N. (1988). The structure and absolute configuration of cyclotirucanenol, a new triterpene from *Euphorbia tirucalli* Linn. Z. Naturforsch. B.

[82] Khan A.Q., Rasheed T., Kazmi S.N.U.H., Ahmed Z., Malik A. (1988). Cycloeuphordenol, a new triterpene from *Euphorbia tirucalli*. Phytochemistry.

[83] Khan A., Ahmed Z., Kazmi N.-u.-K., Malik A. (1987). Further triterpenes from the stem bark of *Euphorbia tirucalli*. Planta Med..

[84] Khan A., Kazmi S., Ahmed Z., Malik A. (1989). Euphorcinol: A new pentacyclic triterpene from *Euphorbia tirucalli*. Planta Med..

[85] Rasool N., Khan A.Q., Malik A. (1989). A Taraxerane Type Triterpene from *Euphorbia tirucalli*. Phytochemistry.

[86] Baslas R.K., Gupta N.C. (1984). Constituents with potential effective agents from the latex of some Euphorbia species. Herba Hung..

[87] Yamamoto Y., Mizuguchi R., Yamada Y. (1981). Chemical Constituents of Cultured Cells of *Euphorbia tirucalli* and *E. millii*. Plant Cell Rep..

[88] Biesboer D.D., Mahlberg P.G. (1979). The effect of medium modification and selected precursors on sterol production by short-term callus cultures of *Euphorbia tirucalli*. J. Nat. Prod..

[89] Nielsen P.E., Nishimura H., Liang Y., Calvin M. (1979). Steroids from Euphorbia and Other Latex-Bearing Plants. Phytochemistry.

[90] Kinghorn A.D. (1979). Characterization of an irritant 4-deoxyphorbol diester from *Euphorbia tirucalli*. J. Nat. Prod..

[91] Fürstenberger G., Hecker E. (1977). New highly irritant Euphorbia factors from latex of *Euphorbia tirucalli* L. Experientia.

[92] Gupta R.K., Mahadevan V. (1967). Chemical examination of the stems of *Euphorbia tirucalli*. Indian J. Pharm..

[93] Ponsinet G., Ourisson G. (1968). Chemotaxonomic Studies in the Family Euphorbiaceae III: Distribution of Triterpenes in the Latexes of Euphorbia. Phytochemistry.

[94] Abdel-Sattar E., Abou-Hussein D., Petereit F. (2015). Chemical constituents from the leaves of *Euphorbia ammak* growing in Saudi Arabia. Pharmacogn. Res..

[95] Hlengwa S.S. (2018). Isolation and Characterisation of Bioactive Compounds from Antidesma Venosum E. Mey. ex Tul. and *Euphorbia cooperi* NE Br. ex A. Berger. Ph.D. Dissertation.

[96] El-Toumy S.A., Salib J.Y., El-Kashak W.A., Marty C., Bedoux G., Bourgougnon N. (2018). Antiviral effect of polyphenol rich plant extracts on herpes simplex virus type 1. Food Sci. Hum. Wellness.

[97] Gschwendt M., Becker E. (1970). Tumor promoting compounds from *Euphorbia triangularis*: Mono-and diesters of 12-desoxy-phorbol. Tetrahedron Lett..

[98] El-Hawary S.S., Mohammed R., Tawfike A.F., Lithy N.M., AbouZid S.F., Amin M.N., Abdelmohsen U.R., Amin E. (2020). Cytotoxic activity and metabolic profiling of fifteen Euphorbia Species. Metabolites.

[99] Boshara O.A.A. (2014). Phytochemical Screening for Leaves, Cortex and Pith of the Cactus *Euphorbia trigona* L. Ph.D. Dissertation.

[100] Kgosiemang I.K., Lefojane R., Direko P., Madlanga Z., Mashele S., Sekhoacha M. (2020). Green synthesis of magnesium and cobalt oxide nanoparticles using *Euphorbia tirucalli*: Characterization and potential application for breast cancer inhibition. Inorg. Nano-Met. Chem..

[101] Kose L.E.S. (2017). Evaluation of Commonly Used Medicinal Plants of Maseru District in Lesotho for Their Ethnobotanical Uses, antimicrobial Properties and Phytochemical Compositions. Ph.D. Dissertation.

[102] Silva V.A.O., Rosa M.N., Miranda-Gonçalves V., Costa A.M., Tansini A., Evangelista A.F., Martinho O., Carloni A.C., Jones C., Lima J.P. (2019). Euphol, a Tetracyclic Triterpene, from *Euphorbia tirucalli* Induces Autophagy and Sensitizes Temozolomide Cytotoxicity on Glioblastoma Cells. Investig. New Drugs.

[103] Yasukawa K., Akihisa T., Yoshida Z.Y., Takido M. (2000). Inhibitory Effect of Euphol, a Triterpene Alcohol from the Roots of *Euphorbia kansui*, on Tumor Promotion by 12-O-Tetradecanoylphorbol-13-Acetate in Two-Stage Carcinogenesis in Mouse Skin. J. Pharm. Pharmacol..

[104] Heliawati L., Kurnia D., Apriyanti E., Adriansyah P.N.A., Ndruru S.T.C.L. (2023). Natural Cycloartane Triterpenoids from *Corypha utan* Lamk. with Anticancer Activity towards P388 Cell Lines and their Predicted Interaction with FLT3. Comb. Chem. High Throughput Screen.

[105] Salomé-Abarca L.F., Gođevac D., Kim M.S., Hwang G.S., Park S.C., Jang Y.P., van den Hondel Cees A.M.J.J., Verpoorte R., Klinkhamer P.G.L., Choi Y.H. (2020). The Instantaneous Multi-Pronged Defense System of Latex against General Plant Enemies. bioRxiv.

[106] Sawale J.A., Patel J.R., Kori M.L. (2019). Antioxidant Properties of Cycloartenol Isolated from *Euphorbia neriifolia* Leaves. Indian J. Nat. Prod..

[107] Niu H., Li X., Yang A., Jin Z., Wang X., Wang Q., Yu C., Wei Z., Dou C. (2018). Cycloartenol Exerts Antiproliferative Effects on Glioma U87 Cells via Induction of Cell Cycle Arrest and p38 MAPK-Mediated Apoptosis. J. Buon.

[108] Zhang Z.L., Luo Z.L., Shi H.W., Zhang L.X., Ma X.J. (2017). Research Advance of Functional Plant Pharmaceutical Cycloartenol about Pharmacological and Physiological Activity. Zhongguo Zhong Yao Za Zhi.

[109] Zare S., Ghaedi M., Heiling S., Asadollahi M., Baldwin I.T., Jassbi A.R. (2015). Phytochemical Investigation on *Euphorbia macrostegia* (Persian Wood Spurge). Iran. J. Pharm. Res..

[110] Barla A., Birman H., Kültür Ş., Öksüz S. (2006). Secondary metabolites from *Euphorbia helioscopia* and their vasodepressor activity. Turk. J. Chem..

[111] Zhang D., Xu H., Wang L., Li Y., Sun P., Wu X., Wang G., Chen W., Ye W. (2015). Betulinic Acid and Its Derivatives as Potential Antitumor Agents. Med. Res. Rev..

[112] Kumari A., Kakkar P. (2012). Lupeol prevents acetaminophen-induced in vivo hepatotoxicity by altering the Bax/Bcl-2 and oxidative stress-mediated mitochondrial signaling cascade. Life Sci..

[113] Borgati T.F., Pereira G.R., Brandão G.C., Oliveira A.B.D. (2012). Synthesis of triazol derivatives of lupeol with potential antimalarial activity. Orbital: Electron. J. Chem..

[114] Siddique H.R., Saleem M. (2011). Beneficial Health Effects of Lupeol Triterpene: A Review of Preclinical Studies. Life Sci..

[115] Gallo M.B., Sarachine M.J. (2009). Biological Activities of Lupeol. Int. J. Pharm. Biomed. Sci..

[116] You Y.J., Nam N.H., Kim Y., Bae K.H., Ahn B.Z. (2003). Antiangiogenic Activity of Lupeol from Bombax Ceiba. Phytother. Res..

[117] Sudhahar V., Kumar S.A., Mythili Y., Varalakshmi P. (2007). Remedial Effect of Lupeol and Its Ester Derivative on Hypercholesterolemia-Induced Oxidative and Inflammatory Stresses. Nutr. Res..

[118] Wen S., Gu D., Zeng H. (2018). Antitumor Effects of Beta-Amyrin in Hep-G2 Liver Carcinoma Cells Are Mediated via Apoptosis Induction, Cell Cycle Disruption and Activation of JNK and P38 Signaling Pathways. J. BUON.

[119] Lin K.-W., Huang A.-M., Tu H.-Y., Lee L.-Y., Wu C.-C., Hour T.-C., Yang S.-C., Pu Y.-S., Lin C.-N. (2011). Xanthine oxidase inhibitory triterpenoid and phloroglucinol from guttiferaceous plants inhibit growth and induced apoptosis in human NTUB1 cells through a ROS-dependent mechanism. J. Agric. Food Chem..

[120] Jabeen K., Javaid A., Ahmad E., Athar M. (2011). Antifungal compounds from Melia azedarach leaves for management of Ascochyta rabiei, the cause of chickpea blight. Nat. Prod. Res..

[121] Shih M.F., Cheng Y.D., Shen C.R., Cherng J.Y. (2010). A Molecular Pharmacology Study into the Anti-inflammatory Actions of *Euphorbia hirta* L. on the LPS-Induced RAW 264.7 Cells through Selective iNOS Protein Inhibition. J. Nat. Med..

[122] Singh A.B., Yadav D.K., Maurya R., Srivastava A.K. (2009). Antihyperglycaemic Activity of α-Amyrin Acetate in Rats and db/db Mice. Nat. Prod. Res..

[123] Abdel-Monem A.R., Abdel-Sattar E., Harraz F.M., Petereit F. (2008). Chemical Investigation of *Euphorbia schimperi* C. Presl. Rec. Nat. Prod..

[124] Johann S., Soldi C., Lyon J.P., Pizzolatti M.G., Resende M.A. (2007). Antifungal activity of the amyrin derivatives and in vitro inhibition of Candida albicans adhesion to human epithelial cells. Lett. Appl. Microbiol..

[125] Vazquez M.M., Apan T.O.R., Lazcano M.E., Bye R. (1999). Anti-Inflammatory Active Compounds from the n-Hexane Extract of *Euphorbia hirta*. J. Mex. Chem. Soc..

[126] Silva F.S., Oliveira P.J., Duarte M.F. (2016). Oleanolic, Ursolic, and Betulinic Acids as Food Supplements or Pharmaceutical Agents for Type 2 Diabetes: Promise or Illusion?. J. Agric. Food Chem..

[127] Foo J.B., Yazan L.S., Tor Y.S., Wibowo A., Ismail N., How C.W., Armania N., Loh S.P., Ismail I.S., Cheah Y.K. (2015). Induction of cell cycle arrest and apoptosis by betulinic acid-rich fraction from Dillenia suffruticosa root in MCF-7 cells involved p53/p21 and mitochondrial signalling pathway. J. Ethnopharmacol..

[128] Damle A.A., Pawar Y.P., Narkar A.A. (2013). Anticancer Activity of Betulinic Acid on MCF-7 Tumors in Nude Mice. Indian J. Exp. Biol..

[129] Esposito F., Sanna C., Del Vecchio C., Cannas V., Venditti A., Corona A., Bianco A., Serrilli A.M., Guarcini L., Parolin C. (2013). *Hypericum hircinum* L. components as new single-molecule inhibitors of both HIV-1 reverse transcriptase-associated DNA polymerase and ribonuclease H activities. Pathog. Dis..

[130] Mertens-Talcott S.U., Noratto G.D., Li X., Angel-Morales G., Bertoldi M.C., Safe S. (2013). Betulinic Acid Decreases ER-Negative Breast Cancer Cell Growth In Vitro and In Vivo: Role of Sp Transcription Factors and microRNA-27a: ZBTB10. Mol. Carcinog..

[131] Kumar S., Kumar V., Prakash O. (2013). Enzymes inhibition and antidiabetic effect of isolated constituents from *Dillenia indica*. Biomed Res. Int..

[132] Alakurtti S., Mäkelä T., Koskimies S., Yli-Kauhaluoma J. (2006). Pharmacological properties of the ubiquitous natural product betulin. Eur. J. Pharm. Sci..

[133] Aiken C., Chen C.H. (2005). Betulinic acid derivatives as HIV-1 antivirals. Trends Mol. Med..

[134] Bernard P., Scior T., Didier B., Hibert M., Berthon J.Y. (2001). Ethnopharmacology and bioinformatic combination for leads discovery: Application to phospholipase A2 inhibitors. Phytochemistry.

[135] Selzer E., Pimentel E., Wacheck V., Schlegel W., Pehamberger H., Jansen B., Kodym R. (2000). Effects of Betulinic Acid Alone and in Combination with Irradiation in Human Melanoma Cells. J. Investig. Dermatol..

[136] Ur Rahman U., Ali S., Khan I., Khan M.A., Arif S. (2016). Anti-Inflammatory Activity of Taraxerol Acetate. J. Med. Sci..

[137] Rehman U.U., Shah J., Khan M.A., Shah M.R., Khan I. (2013). Molecular Docking of Taraxerol Acetate as a New COX Inhibitor. Bangladesh J. Pharmacol..

[138] Tan B., Shi H.L., Ji G., Xie J.Q. (2011). Effects of Taraxerol and Taraxerol Acetate on Cell Cycle and Apoptosis of Human Gastric Epithelial Cell Line AGS. J. Chin. Integr. Med..

[139] Biswas M., Biswas K., Ghosh A., Haldar P. (2009). A pentacyclic triterpenoid possessing anti-inflammatory activity from the fruits of *Dregea volubilis*. Pharmacogn. Mag..

[140] Singh B., Sahu P.M., Sharma M.K. (2002). Anti-inflammatory and Antimicrobial Activities of Triterpenoids from *Strobilanthes callosus* Nees. Phytomedicine.

[141] Takasaki M., Konoshima T., Tokuda K., Masuda K., Arai Y., Shiojima K., Ageta H. (1999). Anti-Carcinogenic Activity of Taraxacum Plant. II. Biol. Pharm. Bull..

[142] Cheng D., Guo Z., Zhang S. (2015). Effect of β-sitosterol on the expression of HPV E6 and p53 in cervical carcinoma cells. Contemp. Oncol..

[143] Nirmal S.A., Pal S.C., Mandal S.C., Patil A.N. (2012). Analgesic and Anti-Inflammatory Activity of β-Sitosterol Isolated from *Nyctanthes arbortristis* Leaves. Inflammopharmacology.

[144] Loizou S., Lekakis I., Chrousos G.P., Moutsatsou P. (2010). β-Sitosterol exhibits anti-inflammatory activity in human aortic endothelial cells. Mol. Nutr. Food Res..

[145] El-Fiky F., Asres K., Gibbons S., Hammoda H., Badr J., Umer S. (2008). Phytochemical and antimicrobial investigation of latex from *Euphorbia abyssinica* Gmel. Nat. Prod. Commun..

[146] Fernandez-Arche A., Saenz M.T., Arroyo M., De la Puerta R., Garcia M.D. (2010). Topical anti-inflammatory effect of tirucallol, a triterpene isolated from *Euphorbia lactea* latex. Phytomedicine.

[147] Akihisa T., Ogihara J., Kato J., Yasukawa K., Ukiya M., Yamanouchi S., Oishi K. (2001). Inhibitory effects of triterpenoids and sterols on human immunodeficiency virus-1 reverse transcriptase. Lipids.

[148] Mali P.Y., Panchal S.S. (2017). *Euphorbia tirucalli* L.: Review on morphology, medicinal uses, phytochemistry and pharmacological activities. Asian Pac. J. Trop. Biomed..

[149] Inoue Y., Shiraishi A., Hada T., Hirose K., Hamashima H., Shimada J. (2004). The antibacterial effects of terpene alcohols on Staphylococcus aureus and their mode of action. FEMS Microbiol. Lett..

[150] Wardana A.P., Abdjan M.I., Aminah N.S., Fahmi M.Z., Siswanto I., Kristanti A.N., Takaya Y. (2022). 3,4,3′-Tri-O-Methylellagic Acid as an Anticancer Agent: In Vitro and In Silico Studies. RSC Adv..

[151] Zhang W.K., Xu J.K., Zhang X.Q., Yao X.S., Ye W.C. (2008). Chemical Constituents with Antibacterial Activity from *Euphorbia sororia*. Nat. Prod. Res..

[152] Abreu C.M., Price S.L., Shirk E.N., Cunha R.D., Pianowski L.F., Clements J.E., Tanuri A., Gama L. (2014). Dual role of novel ingenol derivatives from *Euphorbia tirucalli* in HIV replication: Inhibition of de novo infection and activation of viral LTR. PLoS ONE.

[153] Chen J., Wei S.L., Gao K. (2015). Chemical constituents and antibacterial activities of compounds from Lentinus edodes. Chem. Nat. Compd..

[154] Abdul-Hammed M., Bello I.A., Olajide M., Adedotun I.O., Afolabi T.I., Ibironke A.A., Adebayo B.D. (2023). Exploration of bioactive compounds from Mangifera indica (Mango) as probable inhibitors of thymidylate synthase and nuclear factor kappa-B (NF-Κb) in colorectal cancer management. Phys. Sci. Rev..

[155] Krstić G., Anđelković B., Choi Y.H., Vajs V., Stević T., Tešević V., Gođevac D. (2016). Metabolic changes in *Euphorbia palustris* latex after fungal infection. Phytochemistry.

[156] Anju V., Rameshkumar K.B. (2022). Phytochemical investigation of *Euphorbia trigona*. J. Indian Chem. Soc..

[157] Ogunnusi T.A., Oso B.A., Dosumu O.O. (2010). Isolation and Antibacterial Activity of Triterpenes from *Euphorbia kamerunica* Pax. Int. J. Biol. Chem. Sci..

[158] Sousa G.F.D., Soares D.C.F., Mussel W.D.N., Pompeu N.F.E., Silva G.D.D.F., Vieira Filho S.A., Duarte L.P. (2014). Pentacyclic Triterpenes from Branches of *Maytenus robusta* and In Vitro Cytotoxic Property against 4T1 Cancer Cells. J. Braz. Chem. Soc..

[159] Yang J., Fa J., Li B. (2017). Apoptosis Induction of Epifriedelinol on Human Cervical Cancer Cell Line. Afr. J. Tradit. Complement. Altern. Med..

[160] Sosath S., Ott H.H., Hecker E. (1988). Irritant Principles of the Spurge Family (Euphorbiaceae) XIII. Oligocyclic and Macrocyclic Diterpene Esters from Latices of Some Euphorbia Species Utilized as Source Plants of Honey. J. Nat. Prod..

[161] Ourhzif E.M., Ricelli A., Stagni V., Cirigliano A., Rinaldi T., Bouissane L., Saso L., Chalard P., Troin Y., Khouili M. (2022). Antifungal and Cytotoxic Activity of Diterpenes and Bisnorsesquiterpenoides from the Latex of *Euphorbia resinifera* Berg. Molecules.

[162] Brodie C., Blumberg P.M. (2003). Regulation of cell apoptosis by protein kinase C δ. Apoptosis.

[163] Zayed S., Sorg B., Hecker E. (1984). Structure Activity Relations of Polyfunctional Diterpenes of the Tigliane Type, VI1. Planta Med..

[164] Song J., Ge Z., Yang X., Luo Q., Wang C., You H., Ge T., Deng Y., Lin H., Cui Y. (2015). The Protein Kinase C Agonist Prostratin Induces Differentiation of Human Myeloid Leukemia Cells and Enhances Cellular Differentiation by Chemotherapeutic Agents. Cancer Lett..

[165] Alotaibi D., Amara S., Johnson T.L., Tiriveedhi V. (2018). Potential anticancer effect of prostratin through SIK3 inhibition. Oncol. Lett..

[166] Tsai J.Y., Rédei D., Hohmann J., Wu C.C. (2020). 12-Deoxyphorbol Esters Induce Growth Arrest and Apoptosis in Human Lung Cancer A549 Cells via Activation of PKC-δ/PKD/ERK Signaling Pathway. Int. J. Mol. Sci..

[167] Hirota M., Suttajit M., Suguri H., Endo Y., Shudo K., Wongchai V., Hecker E., Fujiki H. (1988). A new tumor promoter from the seed oil of *Jatropha curcas* L., an intramolecular diester of 12-deoxy-16-hydroxyphorbol. Cancer Res..

[168] Dutra R.C., Claudino R.F., Bento A.F., Marcon R., Schmidt C., Bouzon Z.L., Pianowski L.F., Calixto J.B. (2011). Preventive and therapeutic euphol treatment attenuates experimental colitis in mice. PLoS ONE.

[169] Wu T.S., Lin Y.M., Haruna M., Pan D.J., Shingu T., Chen Y.P., Hsu H.-Y., Nakano T., Lee K.-H. (1991). Antitumor Agents, 119. Kansuiphorins A and B, Two Novel Antileukemic Diterpene Esters from *Euphorbia kansui*. J. Nat. Prod..

[170] Fatope M.O., Zeng L., Ohayaga J.E., Shi G., McLaughlin J.L. (1996). Selectively cytotoxic diterpenes from *Euphorbia poisonii*. J. Med. Chem..

[171] Rizk A.M., Hammouda F.M., El-Missiry M.M., Radwan H.M., Evans F.J. (1985). Biologically Active Diterpene Esters from *Euphorbia peplus*. Phytochemistry.

[172] Kinghorn A.D., Kinghorn A. (1979). Cocarcinogenic irritant Euphorbiaceae. Toxic Plants.

[173] Driedger P.E., Blumberg P.M. (1980). Structure–Activity Relationships in Chick Embryo Fibroblasts for Phorbol-Related Diterpene Esters Showing Anomalous Activities In Vivo. Cancer Res..

[174] Priya C.L., Rao K.V.B. (2011). A Review of Phytochemical and Pharmacological Profile of *Euphorbia tirucalli*. Pharmacologyonline.

[175] Adolf W., Hecker E. (1984). On the Active Principles of the Spurge Family, X. Skin Irritants, Cocarcinogens, and Cryptic Cocarcinogens from the Latex of the Manchineel Tree. J. Nat. Prod..

[176] Abdel-Monem A.R., Abdelrahman E.H. (2016). New abietane diterpenes from *Euphorbia pseudocactus* berger (Euphorbiaceae) and their antimicrobial activity. Pharmacogn. Mag..

[177] Wal A., Wal P., Gupta N., Vishnoi G., Srivastava R.S. (2013). Medicinal Value of *Euphorbia tirucalli*. Int. J. Pharm. Biol..

[178] Ovesná Z., Vachálková A., Horváthová K. (2004). Taraxasterol and β-Sitosterol: New Natural Compounds with Chemoprotective/Chemopreventive Effects. Neoplasma.

[179] Upadhyay B., Singh K.P., Kumar A. (2010). Ethno-Medicinal, Phytochemical, and Antimicrobial Studies of *Euphorbia tirucalli* L. J. Phytol..

[180] Upadhyaya C., Sathish S. (2017). A Review on *Euphorbia neriifolia* Plant. Int. J. Pharm. Chem. Res..

[181] Ahmed S.R., Elsherei M.M., Salah El Dine R., Eltomy S. (2019). Phytoconstituents, Hepatoprotective, and Antioxidant Activities of *Euphorbia cooperi* NE Br. Egypt. J. Chem..

[182] Herawati N., Firdaus F. (2013). 3,3′-Di-O-Methylellagic Acid, an Antioxidant Phenolic Compound from Sonneratia alba Bark. J. Nat. Indones..

[183] Vigbedor B.Y., Akoto C.O., Neglo D. (2022). Isolation and Characterization of 3,3′-Di-O-Methyl Ellagic Acid from the Root Bark of Afzelia africana and Its Antimicrobial and Antioxidant Activities. Sci. Afr..

[184] Aljubiri S.M., Mahmoud K., Mahgoub S.A., Almansour A.I., Shaker K.H. (2021). Bioactive Compounds from *Euphorbia schimperiana* with Cytotoxic and Antibacterial Activities. S. Afr. J. Bot..

[185] Guo Z., Xu Y., Han L., Bo X., Huang C., Ni L. (2011). Antioxidant and cytotoxic activity of the acetone extracts of root of *Euphorbia hylonoma* and its ellagic acid derivatives. J. Med. Plants Res..

[186] Mazoir N., Benharref A., Bailén M., Reina M., González-Coloma A., Martínez-Díaz R.A. (2011). Antileishmanial and Antitrypanosomal Activity of Triterpene Derivatives from Latex of Two Euphorbia Species. Z. Naturforsch. C..

[187] Ekpo O.E., Pretorius E. (2007). Asthma, *Euphorbia hirta* and its anti-inflammatory properties: News and views. S. Afr. J. Sci..

[188] Sultana A., Hossain J., Kuddus R., Rashid M.A., Zahan S., Mitra S., Roy A., Alam S., Sarker M.R., Mohamed I.N. (2022). Ethnobotanical Uses, Phytochemistry, Toxicology, and Pharmacological Properties of *Euphorbia neriifolia* Linn. Against Infectious Diseases: A Comprehensive Review. Molecules.

[189] Tilabi J., Upadhyay R.R. (1983). Adenoma Formation by Ingenol 3, 5, 20-Triacetate. Cancer Lett..

[190] Matsue M., Mori Y., Nagase S., Sugiyama Y., Hirano R., Ogai K., Ogura K., Kurihara S., Okamoto S. (2019). Measuring the Antimicrobial Activity of Lauric Acid against Various Bacteria in Human Gut Microbiota Using a New Method. Cell Transplant..

[191] Chen Y., Li J. (2021). Glutinol inhibits the proliferation of human ovarian cancer cells via PI3K/AKT signaling pathway. Trop. J. Pharm. Res..

[192] Ding Y., Liang C., Kim J.H., Lee Y.M., Hyun J.H., Kang H.K., Kim J.A., Min B.S., Kim Y.H. (2010). Triterpene Compounds Isolated from Acer mandshuricum and Their Anti-Inflammatory Activity. Bioorg. Med. Chem. Lett..

[193] Panda S., Jafri M., Kar A., Meheta B.K. (2009). Thyroid Inhibitory, Antiperoxidative and Hypoglycemic Effects of Stigmasterol Isolated from Butea monosperma. Fitoterapia.

[194] Dinala M.M., Siwe-Noundoub X., Augustyna W., Tembu V.J. (2022). Metabolomics and Phytochemicals of *Euphorbia rowlandii* with Anticancer Properties. J. Ethnopharmacol..

[195] Aghaei M., Yazdiniapour Z., Ghanadian M., Zolfaghari B., Lanzotti V., Mirsafaee V. (2016). Obtusifoliol related steroids from *Euphorbia sogdiana* with cell growth inhibitory activity and apoptotic effects on breast cancer cells (MCF-7 and MDA-MB231). Steroids.

[196] Tong Y., Zhang G., Li Y., Xu J., Yuan J., Zhang B., Hu T., Song G. (2018). Corilagin Inhibits Breast Cancer Growth via Reactive Oxygen Species-Dependent Apoptosis and Autophagy. J. Cell. Mol. Med..

[197] Zheng Z.Z., Chen L.H., Liu S.S., Deng Y., Zheng G.H., Gu Y., Ming Y.L. (2016). Bioguided Fraction and Isolation of the Antitumor Components from *Phyllanthus niruri* L. Biomed Res. Int..

[198] Ming Y., Zheng Z., Chen L., Zheng G., Liu S., Yu Y., Tong Q. (2013). Corilagin Inhibits Hepatocellular Carcinoma Cell Proliferation by Inducing G2/M Phase Arrest. Cell Biol. Int..

[199] Bai X., Pan R., Li M., Li X., Zhang H. (2019). HPLC profile of longan (cv. Shixia) pericarp-sourced phenolics and their antioxidant and cytotoxic effects. Molecules.

[200] Kolodziej H., Burmeister A., Trun W., Radtke O.A., Kiderlen A.F., Ito H. (2005). Tannins and related compounds induce nitric oxide synthase and cytokines gene expressions in Leishmania major-infected macrophage-like RAW 264.7 cells. Bioorg. Med. Chem..

[201] Jin F., Cheng D., Tao J.Y., Zhang S.L., Pang R., Guo Y.J., Zhao L. (2013). Anti-inflammatory and anti-oxidative effects of corilagin in a rat model of acute cholestasis. BMC Gastroenterol..

[202] Cheng J.T., Lin T.C., Hsu F.L. (1995). Antihypertensive effect of corilagin in the rat. Can. J. Physiol. Pharmacol..

[203] Al-Sultan S.I., Hussein Y.A. (2006). Acute toxicity of *Euphorbia heliscopia* in rats. Pak. J. Nutr..

[204] Van Damme P.L. (2001). *Euphorbia tirucalli* for High Biomass Production. Combating Desertification with Plants.

[205] Vogg G., Mattes E., Rothenburger J., Hertkorn N., Achatz S., Sandermann H. (1999). Tumor Promoting Diterpenes from *Euphorbia leuconeura* L. Phytochemistry.

[206] Shlamovitz G.Z., Gupta M., Diaz J.A. (2009). A Case of Acute Keratoconjunctivitis from Exposure to Latex of *Euphorbia tirucalli* (Pencil Cactus). J. Emerg. Med..

[207] Sytwala S., Günther F., Melzig M.F. (2015). Lysozyme- and Chitinase Activity in Latex Bearing Plants of Genus Euphorbia–A Contribution to Plant Defense Mechanism. Plant Physiol. Biochem..

[208] Domsalla A., Görick C., Melzig M.F. (2010). Proteolytic Activity in Latex of the Genus Euphorbia—A Chemotaxonomic Marker?. Pharmazie.

[209] Lynn K.R., Clevette-Radford N.A. (1986). Lectins from latices of Euphorbia and Elaeophorbia species. Phytochemistry.

[210] Arnold H.J., Gulumian M. (1984). Pharmacopoeia of traditional medicine in Venda. J. Ethnopharmacol..

[211] Waczuk E.P., Kamdem J.P., Abolaji A.O., Meinerz D.F., Caeran Bueno D., do Nascimento Gonzaga T.K.S., do Canto Dorow T.S., Boligon A.A., Athayde M.L., da Rocha J.B.T. (2015). *Euphorbia tirucalli* Aqueous Extract Induces Cytotoxicity, Genotoxicity and Changes in Antioxidant Gene Expression in Human Leukocytes. Toxicol. Res..

[212] Silva V.A.O., Rosa M.N., Tansini A., Oliveira R.J., Martinho O., Pianowski L.F., Reis R.M., Lima J.P. (2018). In Vitro Screening of Cytotoxic Activity of Euphol from *Euphorbia tirucalli* on a Large Panel of Human Cancer-Derived Cell Lines. Exp. Ther. Med..

[213] Letícia M., Victório C.P., Costa H.B., Romão W., Kuster R.M., Gattass C.R. (2017). Antiproliferative activity of extracts of *Euphorbia tirucalli* L. (Euphorbiaceae) from three regions of Brazil. Trop. J. Pharm. Res..

[214] Macedo R., Furtado Teixeira L.D., Cristina T. (2021). Analysis of in vitro activity of high dilutions of *Euphorbia tirucalli* L. in human melanoma cells. Int. J. High Dilution Res..

[215] Abdel-Aty A.M., Hamed M.B., Salama W.H., Ali M.M., Fahmy A.S., Mohamed S.A. (2019). Ficus carica, Ficus sycomorus and *Euphorbia tirucalli* latex extracts: Phytochemical screening, antioxidant and cytotoxic properties. Biocatal. Agric. Biotechnol..

[216] Munro B., Vuong Q.V., Chalmers A.C., Goldsmith C.D., Bowyer M.C., Scarlett C.J. (2015). Phytochemical, Antioxidant and Anticancer Properties of *Euphorbia tirucalli* Methanolic and Aqueous Extracts. Antioxidants.

[217] Guillarmod A.J. (1971). Flora of Lesotho (Basutoland).

[218] Mbhele N. (2021). Ethnobotanical Survey, Pharmacological Evaluation and Chemical Characterization of Selected Medicinal Plants Used in South Africa in the Management of Wounds. Ph.D. Dissertation.

[219] Shi Q., Li L., Huo C., Zhang M., Wang Y. (2010). Study on Natural Medicinal Chemistry and New Drug Development. Zhongcaoyao.

[220] Shi Q.W., Su X.H., Kiyota H. (2008). Chemical and Pharmacological Research of the Plants in Genus Euphorbia. Chem. Rev..

[221] Hua J., Liu Y., Xiao C.J., Jing S.X., Luo S.H., Li S.H. (2017). Chemical profile and defensive function of the latex of *Euphorbia peplus*. Phytochemistry.

[222] Goutam M., Sadhan K.R., Jnanojjal C. (2017). *Euphorbia tirucalli* L.: A review on its potential pharmacological use in chronic diseases. Int. J. Sci. Res..

[223] Cowan M.M. (1999). Plant products as antimicrobial agents. Clin. Microbiol. Rev..

[224] Tapas A.R., Sakarkar D.M., Kakde R.B. (2008). Flavonoids as Nutraceuticals: A Review. Trop. J. Pharm. Res..

[225] Abdel-Hameed E.S.S., Bazaid S.A., Salman M.S. (2013). Characterization of the phytochemical constituents of Taif rose and its antioxidant and anticancer activities. Biomed Res. Int..

[226] Mai Z.P., Ni G., Liu Y.F., Li L., Shi G.R., Wang X., Li J.Y., Yu D.Q. (2017). Heliosterpenoids A and B, Two Novel Jatrophane-Derived Diterpenoids with a 5/6/4/6 Ring System from *Euphorbia helioscopia*. Sci. Rep..

[227] Yang Z.G., Jia L.N., Shen Y., Ohmura A., Kitanaka S. (2011). Inhibitory Effects of Constituents from *Euphorbia lunulata* on Differentiation of 3T3-L1 Cells and Nitric Oxide Production in RAW264.7 Cells. Molecules.

[228] Galleggiante V., De Santis S., Cavalcanti E., Scarano A., De Benedictis M., Serino G., Caruso M.L., Mastronardi M., Pinto A., Campiglia P. (2017). Dendritic cells modulate iron homeostasis and inflammatory abilities following quercetin exposure. Curr. Pharm. Des..

[229] Choene M., Motadi L. (2016). Validation of the antiproliferative effects of *Euphorbia tirucalli* extracts in breast cancer cell lines. Mol. Biol..

[230] Seigler D.S. (1998). Alkaloids Derived from Anthranilic Acid. Plant Second. Metab..

[231] Kapingu M.C., Mbwambo Z.H., Moshi M.J., Magadula J.J. (2005). Brine shrimp lethality of a glutarimide alkaloid from Croton sylvaticus Hochst. East Cent. Afr. J. Pharm. Sci..

[232] Boudiar T., Hichem L., Khalfallah A., Kabouche A., Kabouche Z., Brouard I., Bruneau C. (2010). A new alkaloid and flavonoids from the aerial parts of *Euphorbia guyoniana*. Nat. Prod. Commun..

[233] Ndam L.M., Mih A.M., Tening A.S., Fongod A.G.N., Temenu N.A., Fujii Y. (2016). Phytochemical Analysis, Antimicrobial and Antioxidant Activities of *Euphorbia golondrina* L.C. Wheeler (*Euphorbiaceae* Juss.): An Unexplored Medicinal Herb Reported from Cameroon. SpringerPlus.

[234] Demirkapu M.J., Yananli H.R. (2020). Opium Alkaloids. Bioactive Compounds in Nutraceutical and Functional Food for Good Human Health.

[235] Molyneux R.J., Nash R.J., Asano N., Pelletier S.W. (1996). Alkaloids: Chemical and Biological Perspectives.

[236] Bigoniya P., Rana A.C. (2009). Radioprotective and in-vitro cytotoxic sapogenin from *Euphorbia neriifolia* (Euphorbiaceae) leaf. Trop. J. Pharm. Res..

[237] Jannet S.B., Hymery N., Bourgou S., Jdey A., Lachaal M., Magné C., Ksouri R. (2017). Antioxidant and selective anticancer activities of two Euphorbia species in human acute myeloid leukemia. Biomed. Pharmacother..

[238] Glauert A.M., Dingle J.T., Lucy J.A. (1962). Action of saponin on biological cell membranes. Nature.

[239] El Izzi A., Benie T., Thieulant M.L., Le Men-Olivier L., Duval J. (1992). Stimulation of LH release from cultured pituitary cells by saponins of *Petersianthus macrocarpus*: A permeabilizing effect. Planta Med..

[240] Francis G., Kerem Z., Makkar H.P., Becker K. (2002). The biological action of saponins in animal systems: A review. Br. J. Nutr..

[241] Fuchs H., Bachran D., Panjideh H., Schellmann N., Weng A., Melzig M.F., Sutherland M., Bachran C. (2009). Saponins as tool for improved targeted tumor therapies. Curr. Drug Targets.

[242] Gaidi G., Correia M., Chauffert B., Beltramo J.L., Wagner H., Lacaille-Dubois M.A. (2002). Saponins-mediated potentiation of cisplatin accumulation and cytotoxicity in human colon cancer cells. Planta Med..

[243] Jia W.W.G., Bu X., Philips D., Yan H., Liu G., Chen X., Bush J.A., Li G. (2004). Rh2, a compound extracted from ginseng, hypersensitizes multidrug-resistant tumor cells to chemotherapy. Can. J. Physiol. Pharmacol..

[244] Xiao K., Yi Y.H., Wang Z.Z., Tang H.F., Li Y.Q., Lin H.W. (1999). A Cytotoxic Triterpene Saponin from the Root Bark of Aralia Dasyphylla. J. Nat. Prod..

[245] Fattorusso E., Lanzotti V., Taglialatela-Scafati O., Di Rosa M., Ianaro A. (2000). Cytotoxic saponins from bulbs of Allium porrum L. J. Agric. Food Chem..

[246] Tran Q.L., Tezuka Y., Banskota A.H., Tran Q.K., Saiki I., Kadota S. (2001). New Spirostanol Steroids and Steroidal Saponins from Roots and Rhizomes of Dracaena Angustifolia and Their Antiproliferative Activity. J. Nat. Prod..

[247] Yokosuka A., Mimaki Y., Sashida Y. (2002). Spirostanol Saponins from the Rhizomes of Tacca Chantrieri and Their Cytotoxic Activity. Phytochemistry.

[248] Traore F., Faure R., Ollivier E., Gasquet M., Azas N., Debrauwer L., Keita A., Timon-David P., Balansard G. (2000). Structure and Antiprotozoal Activity of Triterpenoid Saponins from Glinus Oppositifolius. Planta Med..

[249] Iorizzi M., Lanzotti V., Ranalli G., De Marino S., Zollo F. (2002). Antimicrobial furostanol saponins from the seeds of Capsicum annuum L. var. acuminatum. J. Agric. Food Chem..

[250] Fraga-Corral M., Otero P., Cassani L., Echave J., Garcia-Oliveira P., Carpena M., Chamorro F., Lourenço-Lopes C., Prieto M.A., Simal-Gandara J. (2021). Traditional applications of tannin rich extracts supported by scientific data: Chemical composition, bioavailability and bioaccessibility. Foods.

[251] Chung K.T., Wong T.Y., Wei C.I., Huang Y.W., Lin Y. (1998). Tannins and human health: A review. Crit. Rev. Food Sci. Nutr..

[252] Amarowicz R., Troszynska A., Barylko-Pikielna N., Shahidi F. (2004). Polyphenolics extracts from legume seeds: Correlations between total antioxidant activity, total phenolics content, tannins content and astringency. J. Food Lipids.

[253] Ho P.L., Yung R.W.H., Tsang D.N.C., Que T.L., Ho M., Seto W.H., Ng T.K., Yam W.C., Ng W.W. (2001). Increasing resistance of Streptococcus pneumoniae to fluoroquinolones: Results of a Hong Kong multicentre study in 2000. J. Antimicrob. Chemother..

[254] Buzzini P., Arapitsas P., Goretti M., Branda E., Turchetti B., Pinelli P., Ieri F., Romani A. (2008). Antimicrobial and antiviral activity of hydrolysable tannins. Mini-Rev. Med. Chem..

[255] Koleckar V., Kubikova K., Rehakova Z., Kuca K., Jun D., Jahodar L., Opletal L. (2008). Condensed and hydrolysable tannins as antioxidants influencing the health. Mini-Rev. Med. Chem..

[256] Chinsembu K.C. (2020). Coronaviruses and nature’s pharmacy for the relief of coronavirus disease 2019. Rev. Bras. Farmacogn..

[257] Mshvildadze V., Legault J., Lavoie S., Gauthier C., Pichette A. (2007). Anticancer Diarylheptanoid Glycosides from the Inner Bark of Betula papyrifera. Phytochemistry.

[258] Liu Q., Tang J.S., Hu M.J., Liu J., Chen H.F., Gao H., Wang G.H., Li S.L., Hao X.J., Zhang X.K. (2013). Antiproliferative cardiac glycosides from the latex of Antiaris toxicaria. J. Nat. Prod..

[259] Yadav A.N., Kour D., Rana K.L., Yadav N., Singh B., Chauhan V.S., Rastegari A.A., Hesham A.E., Gupta V.K. (2019). Metabolic Engineering to Synthetic Biology of Secondary Metabolites Production. New and Future Developments in Microbial Biotechnology and Bioengineering.

[260] Hanson J.R. (2003). Natural Products: The Secondary Metabolites.

[261] Berdy J. (2005). Bioactive microbial metabolites. J. Antibiot..

[262] Kemboi D., Peter X., Langat M., Tembu J. (2020). A review of the ethnomedicinal uses, biological activities, and triterpenoids of Euphorbia species. Molecules.

[263] Langenheim J.H. (1994). Higher plant terpenoids: A phytocentric overview of their ecological roles. J. Chem. Ecol..

[264] Dudareva N., Pichersky E., Gershenzon J. (2004). Biochemistry of Plant Volatiles. Plant Physiol..

[265] Si L., Meng K., Tian Z., Sun J., Li H., Zhang Z., Soloveva V., Li H., Fu G., Xia Q. (2018). Triterpenoids Manipulate a Broad Range of Virus-Host Fusion via Wrapping the HR2 Domain Prevalent in Viral Envelopes. Sci. Adv..

[266] Saleem M. (2009). Lupeol, a Novel Anti-inflammatory and Anti-cancer Dietary Triterpene. Cancer Lett..

[267] Erazo S., Rocco G., Zaldivar M., Delporte C., Backhouse N., Castro C., Belmonte E., Monache F.D., García R. (2008). Active Metabolites from *Dunalia spinosa* Resinous Exudates. Zeitschrift für Naturforschung C.

[268] Imam S., Iqbal Azhar M. (2007). Two triterpenes lupanone and lupeol, isolated and identified from Tamarindus indica. Linn. Pak. J. Pharm. Sci..

[269] De Miranda A., Silva J., Rezende C., Neves J., Parrini S., Pinheiro M., Cordeiro M., Tamborini E., Pinto A. (2000). Antiinflammatory and analgesic activities of the latex containing triterpenes from *Himatanthus sucuuba*. Planta Med..

[270] Nguemfo E.L., Dimo T., Dongmo A.B., Azebaze A.G.B., Alaoui K., Asongalem A.E., Cherrah Y., Kamtchouing P. (2009). Anti-Oxidative and Anti-Inflammatory Activities of Some Isolated Constituents from the Stem Bark of Allanblackia monticola Staner L. *C. (Guttiferae)*. Inflammopharmacology.

[271] Gupta R., Sharma A.K., Sharma M.C., Dobhal M.P., Gupta R.S. (2012). Evaluation of antidiabetic and antioxidant potential of lupeol in experimental hyperglycaemia. Nat. Prod. Res..

[272] Ragasa C.Y., Cornelio K.B. (2013). Triterpenes from *Euphorbia hirta* and Their Cytotoxicity. Chin. J. Nat. Med..

[273] Oriakhi K., Uadia P.O., Shaheen F., Jahan H., Ibeji C.U., Iqbal C.M. (2014). Isolation, Characterization, and Hepatoprotective Properties of Betulinic Acid and Ricinine from Tetracarpidium conophorum Seeds (Euphorbiaceae). J. Food Biochem..

[274] Mbeunkeu A.B.D., Azebaze A.G.B., Tala M.F., Teinkela J.E.M., Noundou X.S., Krause R.W.M., Vardamides J.C., Laatsch H. (2018). Three New Pentacyclic Triterpenoids from Twigs of *Manniophyton fulvum* (Euphorbiaceae). Phytochem. Lett..

[275] Banzouzi J.T., Soh P.N., Ramos S., Toto P., Cavé A., Hemez J., Benoit-Vical F. (2015). Samvisterin, a new natural antiplasmodial betulin derivative from Uapaca paludosa (Euphorbiaceae). J. Ethnopharmacol..

[276] Sekhoacha M., Campbell W., Smith P. (2009). In vitro and in vivo antimalarial activity of DCM extract of Agathosma betulina. Afr. J. Tradit. Complement. Altern. Med..

[277] Tamamura H., Kobayakawa T., Ohashi N. (2018). Springer Briefs in Pharmaceutical Science and Drug Development.

[278] Mukherjee P.K., Saha K., Das J., Pal M., Saha B.P. (1997). Studies on the Anti-Inflammatory Activity of Rhizomes of Nelumbo nucifera. Planta Med..

[279] Bigoniya P., Rana A. (2008). A comprehensive phyto-pharmacological review of *Euphorbia neriifolia* Linn. Pharmacogn. Rev..

[280] Chaturvedula V.P., Schilling J.K., Miller J.S., Andriantsiferana R., Rasamison V.E., Kingston D.G. (2004). New cytotoxic terpenoids from the wood of Vepris punctata from the Madagascar Rainforest. J. Nat. Prod..

[281] Cao S., Brodie P., Miller J.S., Birkinshaw C., Rakotondrafara A., Andriantsiferana R., Rasamison V.E., Kingston D.G.I. (2009). Antiproliferative compounds of Helmiopsis sphaerocarpa from the Madagascar rainforest. Nat. Prod. Res..

[282] Csupor-Löffler B., Hajdú Z., Zupkó I., Molnár J., Forgo P., Vasas A. (2011). Antiproliferative constituents of the roots of Conyza canadensis. Planta Med..

[283] Min B.-S., Na M.-K., Oh S.-R., Ahn K.-S., Jeong G.-S., Li G., Lee S.-K., Joung H., Lee H.-K. (2004). New Furofuran and Butyrolactone Lignans with Antioxidant Activity from the Stem Bark of *Styrax japonica*. J. Nat. Prod..

[284] Sangeetha K.N., Sujatha S., Muthusamy V.S., Anand S., Nithya N., Velmurugan D., Balakrishnan A., Lakshmi B.S. (2010). 3β-Taraxerol of Mangifera indica, a PI3K-Dependent Dual Activator of Glucose Transport and Glycogen Synthesis in 3T3-L1 Adipocytes. Biochim. Biophys. Acta Gen. Subj..

[285] Carréu J.P.M. (2020). Bioactive Terpenoids from *Euphorbia pubescens*: Isolation and Derivatization. Master’s Dissertation.

[286] Anju V., Singh A., Shilpa G., Kumar B., Priya S., Sabulal B., Rameshkumar K.B. (2018). Terpenes and biological activities of *Euphorbia tortilis*. Lett. Org. Chem..

[287] Anjaneyulu V., Satyanarayana P., Viswanadham K.N., Jyothi V.G., Rao K.N., Radhika P. (1999). Triterpenoids from Mangifera indica. Phytochemistry.

[288] Vilahur G., Ben-Aicha S., Diaz-Riera E., Badimon L., Padró T. (2019). Phytosterols and Inflammation. Curr. Med. Chem..

[289] Vezza T., Canet F., de Marañón A.M., Bañuls C., Rocha M., Víctor V.M. (2020). Phytosterols: Nutritional Health Players in the Management of Obesity and Its Related Disorders. Antioxidants.

[290] Kazłowska K., Lin H.T.V., Chang S.H., Tsai G.J. (2013). In vitro and in vivo anticancer effects of sterol fraction from red algae *Porphyra dentata*. Evid. Based Complement. Altern. Med..

[291] Kangsamaksin T., Chaithongyot S., Wootthichairangsan C., Hanchaina R., Tangshewinsirikul C., Svasti J. (2017). Lupeol and stigmasterol suppress tumor angiogenesis and inhibit cholangiocarcinoma growth in mice via downregulation of tumor necrosis factor-α. PLoS ONE.

[292] De P.T., Urones J.G., Marcos I.S., Basabe P., Cuadrado M.S., Moro R.F. (1987). Triterpenes from *Euphorbia broteri*. Phytochemistry.

[293] Öksüz S., Gürek F., Lin L.Z., Gil R.R., Pezzuto J.M., Cordell G.A. (1996). Aleppicatines A and B from *Euphorbia aleppica*. Phytochemistry.

[294] Öksüz S., Ulubelen A., Barla A., Voelter W. (2002). Terpenoids and Aromatic Compounds from *Euphorbia heteradena*. Turk. J. Chem..

[295] Miranda R.D.S., de Jesus B.D.S.M., da Silva Luiz S.R., Viana C.B., Adao Malafaia C.R., Figueiredo F.D.S., Martins R.C.C. (2022). Anti-Inflammatory Activity of Natural Triterpenes—An Overview from 2006 to 2021. Phytother. Res..

[296] Tanaka R., Kasubuchi K., Kita S., Matsunaga S. (1999). Obtusifoliol and Related Steroids from the Whole Herb of *Euphorbia chamaesyce*. Phytochemistry.

[297] National Center for Biotechnology Information “PubChem Compound Summary for CID 9932254, Glutinol”. PubChem. https://pubchem.ncbi.nlm.nih.gov/compound/Glutinol.

[298] Latansio de Oliveira T., Reder Custodio de Souza A., Dias Fontana P., Carvalho Carneiro M., Beltrame F.L., de Messias Reason I.J., Bavia L. (2022). Bioactive secondary plant metabolites from *Euphorbia umbellata* (PAX) Bruyns (Euphorbiaceae). Chem. Biodivers..

[299] De Oliveira T.L., Munhoz A.C.M., Lemes B.M., Minozzo B.R., Nepel A., Barison A., Fávero G.M., Campagnoli E.B., Beltrame F.L. (2013). Antitumoral effect of Synadenium grantii Hook f. (Euphorbiaceae) latex. J. Ethnopharmacol..

[300] Duong T.-H., Beniddir M.A., Genta-Jouve G., Nguyen H.-H., Nguyen D.-P., Nguyen T.-A., Mac D.-H., Boustie J., Nguyen K.-P., Chavasiri W. (2019). Further terpenoids from *Euphorbia tirucalli*. Fitoterapia.

[301] Akihisa T., Yasukawa K., Oinuma H., Kasahara Y., Yamanouchi S., Takido M., Tamura T. (1996). Triterpene alcohols from the flowers of compositae and their anti-inflammatory effects. Phytochemistry.

[302] Schmidt R.J., Evans F.J. (1977). Candletoxins A and B, 2 new aromatic esters of 12-deoxy-16-hydroxy-phorbol, from the irritant latex of *Euphorbia poisonii* Pax. Experientia.

[303] Hecker E. (1977). New toxic, irritant and cocarcinogenic diterpene esters from Euphorbiaceae and from Thymelaeaceae. Pure Appl. Chem..

[304] Sakata K., Kawazu K., Mitsui T. (1971). Studies on a Piscicidal Constituent of *Hura crepitans*: Part II. Chemical Structure of Huratoxin. Agric. Biol. Chem..

[305] Karalai C., Wiriyachitra P., Sorg B., Hecker E. (1995). Medicinal plants of Euphorbiaceae occurring and utilized in Thailand. V. Skin irritants of the daphnane and tigliane type in latex of Excoecaria bicolor and the uterotonic activity of the leaves of the tree. Phytother. Res..

[306] Yang M.H., Baek S.H., Hwang S.T., Um J.Y., Ahn K.S. (2022). Corilagin Exhibits Differential Anticancer Effects through the Modulation of STAT3/5 and MAPKs in Human Gastric Cancer Cells. Phytother. Res..

[307] Chen L., Chen R., Wei K. (1992). Constituents of tannins from *Euphorbia prostrata* Ait. Zhongguo Zhong Yao Za Zhi = Zhongguo Zhongyao Zazhi = China J. Chin. Mater. Med..

[308] Yang M.H., Vasquez Y., Ali Z., Khan I.A., Khan S.I. (2013). Constituents from Terminalia Species Increase PPARα and PPARγ Levels and Stimulate Glucose Uptake without Enhancing Adipocyte Differentiation. J. Ethnopharmacol..

[309] Latté K.P., Kolodziej H. (2000). Antifungal effects of hydrolysable tannins and related compounds on dermatophytes, mould fungi and yeasts. Z. Naturforsch. C.

[310] Notka F., Meier G.R., Wagner R. (2003). Inhibition of Wild-Type Human Immunodeficiency Virus and Reverse Transcriptase Inhibitor-Resistant Variants by *Phyllanthus amarus*. Antiviral Res..

[311] Aljubiri S.M., Elsalam E.A., Abd El Hady F.K., Radwan M.O., Almansour A.I., Shaker K.H. (2023). In Vitro Acetylcholinesterase, Tyrosinase Inhibitory Potentials of Secondary Metabolites from *Euphorbia schimperiana* and *Euphorbia balsamifera*. Zeitschrift für Naturforschung C.

[312] Lan Y.H., Yen C.H., Leu Y.L. (2020). Chemical constituents from the aerial parts of *Euphorbia formosana* Hayata and their chemotaxonomic significance. Biochem. Syst. Ecol..

[313] Wu Y., Zhang H., Zhang R., Cao G., Li Q., Zhang B., Wang Y., Yang C. (2021). Serum Metabolome and Gut Microbiome Alterations in Broiler Chickens Supplemented with Lauric Acid. Poult. Sci..

[314] Baloch I.B., Baloch M.K. (2010). Irritant and co-carcinogenic diterpene esters from the latex of *Euphorbia cauducifolia* L. J. Asian Nat. Prod. Res..

[315] Kedei N., Lundberg D.J., Toth A., Welburn P., Garfield S.H., Blumberg P.M. (2004). Characterization of the interaction of ingenol 3-angelate with protein kinase C. Cancer Res..

[316] Ogbourne S.M., Suhrbier A., Jones B., Cozzi S.-J., Boyle G.M., Morris M., McAlpine D., Johns J., Scott T.M., Sutherland K.P. (2004). Antitumor Activity of 3-Ingenyl Angelate: Plasma Membrane and Mitochondrial Disruption and Necrotic Cell Death. Cancer Res..

[317] Hampson P., Chahal H., Khanim F., Hayden R., Mulder A., Assi L.K., Bunce C.M., Lord J.M. (2005). PEP005, a selective small-molecule activator of protein kinase C, has potent antileukemic activity mediated via the delta isoform of PKC. Blood.

[318] Nothias-Scaglia L.F., Litaudon M., Sauvain M., Costa J. (2015). Diterpene Esters as Promising Therapeutic Agents to Inhibit HIV Replication. J. Nat. Prod..

[319] Gyebi G., Ogunyemi O., Ibrahim I., Afolabi S., Ojo R., Ejike U., Adebayo J. (2022). Inhibitory Potentials of Phytocompounds from Ocimum Gratissimum against Anti-Apoptotic BCL-2 Proteins Associated with Cancer: An Integrated Computational Study. Egypt. J. Basic Appl. Sci..

[320] Lipinski C.A. (2004). Lead- and Drug-Like Compounds: The Rule-of-Five Revolution. Drug Discov. Today Technol..

[321] Amin M.L. (2013). P-Glycoprotein Inhibition for Optimal Drug Delivery. Drug Target Insights.

[322] White R.E., Chackalamannil S., Rotella D., Ward S.E. (2017). Role of ADME/PK in Drug Discovery, Safety Assessment, and Clinical Development. Comprehensive Medicinal Chemistry III.

[323] Kratz J.M., Grienke U., Scheel O., Mann S.A., Rollinger J.M. (2017). Natural Products Modulating the hERG Channel: Heartaches and Hope. Nat. Prod. Rep..

