# Supplementary material for: Pharmacological Significance, Medicinal Use, and Toxicity of Extracted and Isolated Compounds from Euphorbia Species Found in Southern Africa: A Review
Source: Plants (Basel). 2025 Feb 5;14(3):469. doi: 10.3390/plants14030469 (PMC11821031; doi:10.3390/plants14030469)
Supplement: Supplementary file 1 [file plants-14-00469-s001.zip › plants-3355477-supplementary.pdf]

Supplementary Material:

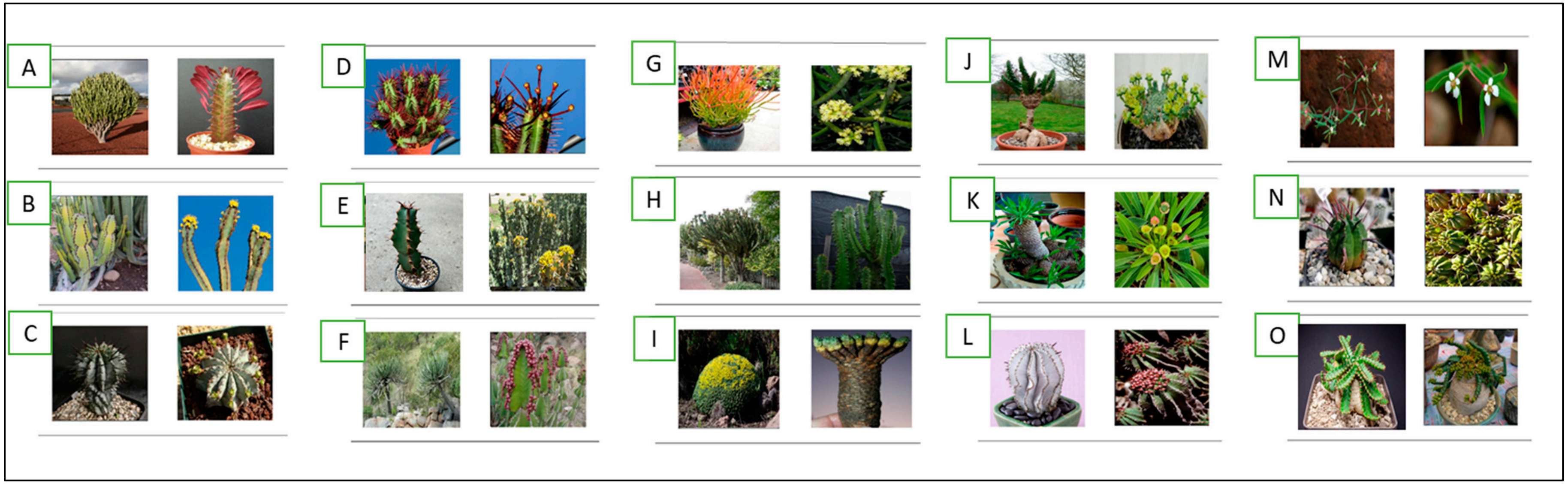

Figure S1. Illustrations of various medicinal plants belonging to the *Euphorbia* genus. S1A: *Euphorbia* trigona tree and pot plant (source: https://tropical.theferns.info/image.php?id=Euphorbia+trigona; https://laidbackgardener.blog/2018/12/09/when-a-red-euphorbia-turns-green/ (accessed 6 February 2025)); S1B: *Euphorbia* ledienii plant with stem and flowers; S1C: *Euphorbia* horrida plant with stem and flowers; S1D: *Euphorbia* enopla plant with stem and flowers; S1E: *Euphorbia* coerulescences plant with stem and flowers (source: https://serreslavoie.com/en/products/euphorbia-coerulescens; https://www.agaveville.org/viewtopic.php?t=2025 (accessed 6 February 2025)). S1F: *Euphorbia cooperi* tree and fruits (source: https://pza.sanbi.org/euphorbia-cooperi (accessed 6 February 2025)); S1G: *Euphorbia tirucalli* plant and flower (source: https://za.pinterest.com/pin/298785756510953587/; https://za.pinterest.com/pin/474426141998696380/ (accessed 6 February 2025)). S1H *Euphorbia ammak* tree and flower (source: https://luirig.altervista.org/cpm/albums/bot-units07/euphorbia-ammak16454.jpg (accessed 6 February 2025)); S1I: *Euphorbia clavarioides* plant with flowers and stem (source: https://www.inaturalist.org/observations/38217609; https://www.llifle.com/Encyclopedia/SUCCULENTS/Family/Euphorbiaceae/32951/Euphorbia_clavarioides_var._truncata (accessed 6 February 2025)). S1J: *Euphorbia gorgonis* plant with flowers and stem (source: https://www.bihrmann.com/caudiciforms/SUBS/eup-gor-sub.asp (accessed 6 February 2025)). S1K: *Euphorbia bupleurifolia* plant with stem and flowers (source: https://www.cactus-art.biz/schede/EUPHORBIA/Euphorbia_bupleurifolia/Euphorbia_bupleurifolia/Euphorbia_bupleurifolia.htm; https://en.wikipedia.org/wiki/Euphorbia_bupleurifolia (accessed 6 February 2025)). S1L: *Euphorbia polygona* plant with stem and flowers (source: https://www.biodiversityexplorer.info/plants/euphorbiaceae/euphorbia_polygona.htm (accessed 6 February 2025)). S1M: *Euphorbia Arabica* plant with stem and flowers (source: https://www.zimbabweflora.co.zw/speciesdata/species.php?species_id=136210; https://www.biodiversityexplorer.info//plants/euphorbiaceae/images/136210-2_658w.jpg

(accessed 6 February 2025); S1N: *Euphorbia ferox* plant with stem and flowers (source https://planetdesert.com/products/euphorbia-ferox-cactus-cacti-real-succulent-plant; https://www.cactofili.org/specie.asp?gen=euphorbia&sp=ferox (accessed 6 February 2025)). S1O: *Euphorbia stellate* plant with stem and flowers (source: https://worldofsucculents.com/euphorbia-stellata/#google_vignette (accessed 4 April 2024); https://www.cactofili.org/specie.asp?gen=euphorbia&sp=stellata (7 February 2025).

.

Table S1. Compounds with high cytotoxic probability against 3 or more cancer cell lines.

**Compounds**	**Pa**	**Pi**	**Cancer cell line**	**Cell line full name**	**Tissue**	**Tumor type**
Euphol (C1)	0.591	0.002	MKN- 7	Gastric carcinoma	Stomach	Carcinoma
	0.555	0.014	UO-31	Renal carcinoma	Kidney	Carcinoma
	0.554	0.014	SK-MEL-2	Melanoma	Skin	Melanoma
Cycloartenol (C2)	0.783	0.001	MKN-7	Gastric carcinoma	Stomach	Carcinoma
	0.577	0.015	HepG2	Hepatoblastoma	Liver	Hepatoblastoma
	0.523	0.029	DMS-114	Lung carcinoma	Lung	Carcinoma
	0.501	0.017	U-251	Glioma	Brain	Glioma
Lupeol (C3)	0.785	0.003	8505C	Thyroid-gland-undifferentiated carcinoma	Thyroid	Carcinoma
	0.62	0.001	FaDu	Hypopharyngeal squamous carcinoma	Upper aerodigestive tract	Carcinoma
	0.563	0.013	SK-MEL- 2	Melanoma	Skin	Melanoma
	0.539	0.004	DLD-1	Colon adenocarcinoma	Colon	Adenocarcinoma
	0.526	0.005	SW480	Colon adenocarcinoma	Colon	Adenocarcinoma

Alpha-amyrin (C4)	0.52	0.005	PANC-1	Pancreatic carcinoma	Pancreas	Carcinoma
	0.632	0.004	8505C	Thyroid-gland-undifferentiated (anaplastic) carcinoma	Thyroid	Carcinoma
	0.523	0.003	MKN-7	Gastric carcinoma	Stomach	Carcinoma
Betulinic acid (C5)	0.695	0.003	8505C	Thyroid-gland-undifferentiated (anaplastic) carcinoma	Thyroid	Carcinoma
	0.667	0.001	FaDu	Hypopharyngeal-squamous carcinoma	Upper aerodigestive tract	Carcinoma
	0.562	0.013	SK-MEL-2	Melanoma	Skin	Melanoma
24-Methylene-cycloartanol (C6)	0.555	0.003	MKN-7	Gastric carcinoma	Stomach	Carcinoma
	0.539	0.023	HL-60	Promyeloblast leukemia	Haematopoietic and lymphoid tissue	Leukemia
	0.52	0.031	DMS-114	Lung carcinoma	Lung	Carcinoma
	0.505	0.026	HL-60	Promyeloblast leukemia	Haematopoietic and lymphoid tissue	Leukemia

12-Deoxyphorbol-13-tiglate-20-acetate (C7)	0.638	0.033	A549	Lung carcinoma	Lung	Carcinoma
	0.568	0.024	NCI-H838	Non-small cell lung cancer. 3 stage	Lung	Carcinoma
	0.556	0.021	HL-60	Promyeloblast leukemia	Haematopoietic and lymphoid tissue	Leukemia
Ingol-7,8,12-acetate,ditiglate (C8)	0.663	0.012	NCI-H838	Non-small cell lung cancer. 3 stage	Lung	Carcinoma
	0.592	0.005	SK-MEL-1	Metastatic melanoma	Skin	Melanoma
	0.513	0.033	DMS-114	Lung carcinoma	Lung	Carcinoma
Diterpene glycoside (C9)	0.682	0.008	SK-MEL-2	Melanoma	Skin	Melanoma
	0.614	0.015	HL-60	Promyeloblast leukemia	Haematopoietic and lymphoid tissue	Leukemia
	0.534	0.034	NCI-H838	Non-small cell lung cancer. 3 stage	Lung	Carcinoma
Tirucallol (C10)	0.591	0.002	MKN-7	Gastric carcinoma	Stomach	Carcinoma
	0.555	0.014	UO-31	Renal carcinoma	Kidney	Carcinoma
	0.554	0.014	SK-MEL-2	Melanoma	Skin	Melanoma

Isobutyl angelate (C11)	0.616	0.012	HepG2	Hepatoblastoma	Liver	Hepatoblastoma
	0.545	0.03	NCI-H838	Non-small cell lung cancer. 3 stage	Lung	Carcinoma
	0.544	0.052	A549	Lung carcinoma	Lung	Carcinoma
Kaempferol-3-O-rutinoside (C12)	0.546	0.004	Caco-2	Colon adenocarcinoma	Colon	Adenocarcinoma
	0.548	0.016	SK-MEL-1	Metastatic melanoma	Skin	Melanoma
	0.55	0.022	HL-60	Promyeloblast leukemia	Haematopoietic and lymphoid tissue	Leukemia
	0.542	0.031	NCI-H838	Non-small cell lung cancer. 3 stage	Lung	Carcinoma

*Pa - Probability to be Active, Pi - Probability to be Inactive.

Table S2. Physicochemical parameters and solubility tendency.

**Descriptors**	**Euphol**	**Cycloartenol**	**Lupeol**	**α-amyrin**	**Betulinic acid**	**24-Methylene-cycloartanol**
**Physiochemical characteristic**						
Molecular weight (g/mol)	426.72	426.72	426.7 2	426.72	456.70	440.74
Num.rotatable bonds	4	4	1	0	2	5
H-bond acceptors	1	1	1	1	1	1

H-bond donors	1	1	1	1	2	1
Molar Refractivity	137.04	135.14	135.1 4	135.14	136.91	139.95
TPS	20.23	20.23	20.23	20.23	57.53	20.23
Solubility (Log Po/w)	5.09	5.17	4.68	4.77	3.79	5.20
Solubility class	Poorly soluble	Poorly soluble	Poorly soluble	Poorly soluble	Poorly soluble	Poorly soluble
**Drug-likeness**						
Lipinski	Yes	Yes	Yes	Yes	Yes	Yes
Bioavailabilitv	0.55	0.55	0.55	0.55	0.85	0.55
PAINS	0 alert	0 alert	0 alert	0 alert	0 alert	0 alert
Brenk	1 alert	1 alert	1 alert	1 alert	1 alert	1 alert
Synthetic accessibility	Yes	Yes	Yes	Yes	Yes	Yes
**Absorption**						
Caco2 permeability(log Papp in 10-6 cm/s)	1.203	1.194	1.226	1.227-J	1.175	1.221
Human intestinal absorption (% Absorbed)	93.119	95.248	95.782	94.062	99.763	95.309
P-glycoprotein SUbstrate	Neg.	Neg.	Neg.	Neg.	Neg.	Neg.
P-glycoprotein Iinhibitor	Pos.	Pos.	Pos.	Pos.	Neg.	Neg.
P-glycoprotein II inhibitor	Pos.	Pos.	Pos.	Pos.	Neg.	Pos.
**Distribution**						

VDss (human) (log L/kg)	0.661	-0.075	0	*0.266*	-1.18	-0.072
BBB permeability (Log BB)	0.683	0.794	0.726	0.674,	-0.322	0.845
CNS permeability (Log PS)	-2.254	-1.714	-1.714	-1.773	-1.343	-1.462
**Metabolism**						
CYP2D6 substrate	Neg.	Neg.	Neg.	Neg.	Neg.	Neg.
CYP3A4 substrate	Pos.	Pos.	Pos.	Pos.	Pos.	Pos.
CYP1A2 inhibition	Neg.	Neg.	Neg.	Neg.	Neg.	Neg.
CYP2C19 inhibitior	Neg.	Neg.	Neg.	Neg.	Neg.	Neg.
CYP2C9 inhibitior	Neg.	Neg.	Neg.	Neg.	Neg.	Neg.
CYP2D6 inhibitior	Neg.	Neg.	Neg.	Neg.	Neg.	Neg.
CYP3A4 inhibitior	Neg.	Neg.	Neg.	Neg.	Neg.	Neg.
**Excretion**						
Total Clearance (log ml/min/kg)	0.403	0.262	0.153	0.119	0.116	0.255
Renal OCT2 substrate	Neg.	Neg.	Neg.	Neg.	Neg.	Neg.
**Toxicity**						
Max. tolerated dose (human) (log mg/kg/day)	-0.568	-0.46	0.502	-0.571	0.144	0.303
hERG Iinhibitor	Neg.	Neg.	Neg.	Neg.	Neg.	Neg.
Oral Rat Acute _ Toxicity (LOSO)(mol/ka)	1.906	2.627	2.563	2.4	2.256	2.542

Oral Rat Chronic Toxicity (LOAEL)((log mg/ka bw/day)	0.788	0.806	0.8	0.856	2.206	0.802
Hepatotoxicity	Neg.	Neg.	Neg.	Neg.	Pos.	Neg.

[Neg. – Negative; Pos. – Positive].
